# Supplementary material for: Oxidative Stress in Cells with Extra Centrosomes Drives Non-Cell-Autonomous Invasion
Source: Dev Cell. 2018 Nov 19;47(4):409–424.e9. doi: 10.1016/j.devcel.2018.10.026 (PMC6251975; doi:10.1016/j.devcel.2018.10.026)
Supplement: Document S1. Figures S1–S7 and Tables S1, S6, and S7 [file mmc1.pdf]

Developmental Cell, Volume *47*

Supplemental Information

Oxidative Stress in Cells with Extra Centrosomes

Drives Non-Cell-Autonomous Invasion

Teresa Arnandis, Pedro Monteiro, Sophie D. Adams, Victoria Louise Bridgeman, Vinothini Rajeeve, Emanuela Gadaleta, Jacek Marzec, Claude Chelala, Ilaria Malanchi, Pedro R. Cutillas, and Susana A. Godinho

Figure S1

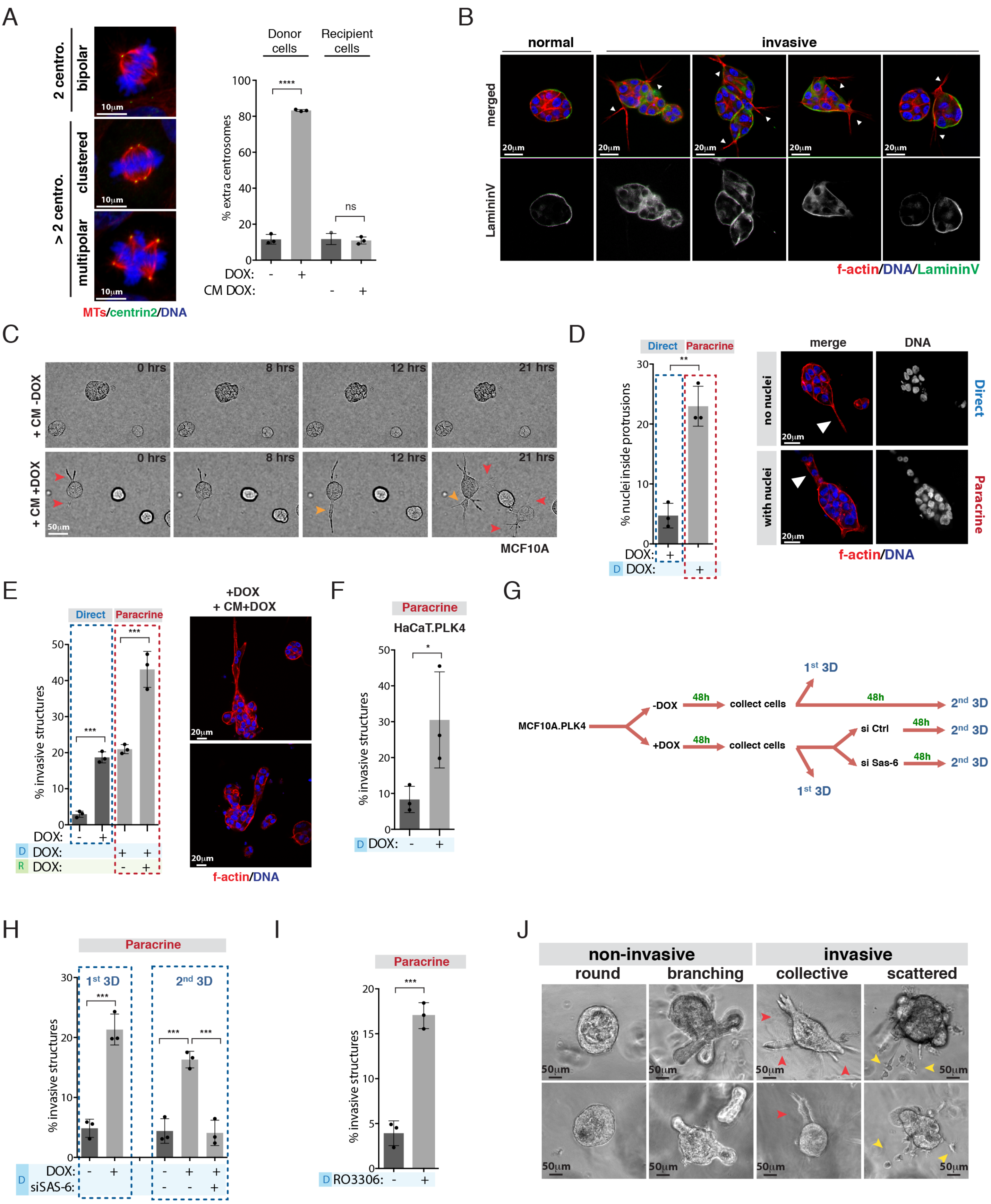

A
2 centro.
bipolar
> 2 centro.
clustered
multipolar
10μm
MTs/centrin2/DNA
Donor cells
Recipient cells
% extra centrosomes
****
ns
DOX: - +
CM DOX: - +
B
normal
invasive
merged
LamininV
20μm
f-actin/DNA/LamininV
C
+ CM -DOX
+ CM +DOX
0 hrs
8 hrs
12 hrs
21 hrs
50μm
MCF10A
D
Direct
Paracrine
% nuclei inside protrusions
**
DOX: +
D DOX: +
merge
DNA
no nuclei
with nuclei
Direct
Paracrine
20μm
f-actin/DNA
E
Direct
Paracrine
% invasive structures
***
DOX: - +
D DOX: + +
R DOX: - +
+DOX
+ CM+DOX
20μm
f-actin/DNA
F
Paracrine
HaCaT.PLK4
% invasive structures
*
D DOX: - +
G
MCF10A.PLK4
-DOX
+DOX
48h
collect cells
1st 3D
2nd 3D
si Ctrl
si Sas-6
H
Paracrine
1st 3D
2nd 3D
% invasive structures
***
D DOX: - + - + +
siSAS-6: - - +
I
Paracrine
% invasive structures
***
D RO3306: - +
J
non-invasive
invasive
round
branching
collective
scattered
50μm

Figure S1. Centrosome amplification induces paracrine invasion. Related to Figure 1, Table S1, Videos S3 and S4. (A) Left, Cells in mitosis with normal and amplified centrosomes (>2 centro.; clustered + multipolar) were stained for microtubules (MTs) (α-Tubulin, red), centrioles (centrin2, green) and DNA (blue). Scale bar 10μm. Right, Centrosome amplification in cells with extra centrosomes (+DOX) or incubated with CM collected from cells with extra centrosomes (CM+DOX). (B) Normal and invasive 3D acini stained for f-actin (phalloidin, red), laminin V (green) and DNA (blue). White arrowheads indicate invasive protrusions. Scale bar 20μm. (C) Still images from live cell imaging of acini incubated with CM (CM-/+DOX). Red arrowheads indicate invasive acini and orange arrowhead indicates a cell/nuclei moving through the invasive protrusion. Scale bar 20μm (D) Left, Quantification of acini with invasive protrusions containing nuclei in cells with extra centrosomes (+DOX) or incubated with CM (CM-/+DOX). Right, Invasive acini stained for f-actin (phalloidin, red) and DNA (blue). Arrowheads indicate invasive protrusions. Scale bar 20μm. (E) Left, Quantification of invasive structures in acini with extra centrosomes (+DOX) and incubated with CM+DOX. Right, Highly invasive/disrupted acini observed in cells with extra centrosomes treated with CM+DOX. Cells were stained for f-actin (phalloidin, red) and DNA (blue). Scale bar 20μm. (F) Quantification of invasive acini incubated with CM from HaCaT.PLK4 cells. (G) Schematic representation of SAS-6 depletion to revert centrosome amplification. (H) Quantification of invasive acini incubated with CM from cells where centrosome amplification was lost (SAS-6 siRNA). (I) Invasive acini in cells incubated with CM collected from cells treated with the CDK1 inhibitor RO-3306 (5μM, 40 hrs). (J) Bright field images of non-invasive and invasive mouse mammary organoids. Scale bar 50μm. For all graphics error bars represent mean +/- SD from three independent experiments. *$P < 0.05$, **$P < 0.01$, ***$P < 0.001$, ****$P < 0.0001$, ns not significant.

Figure S2

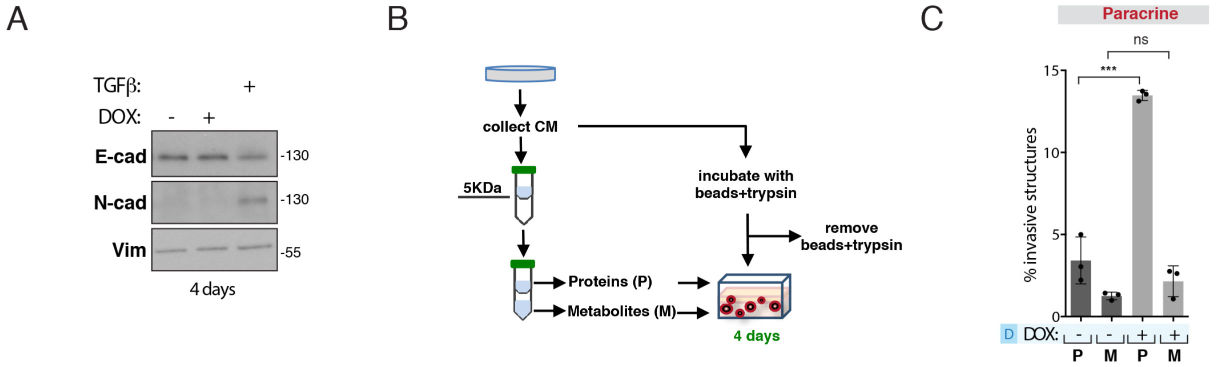

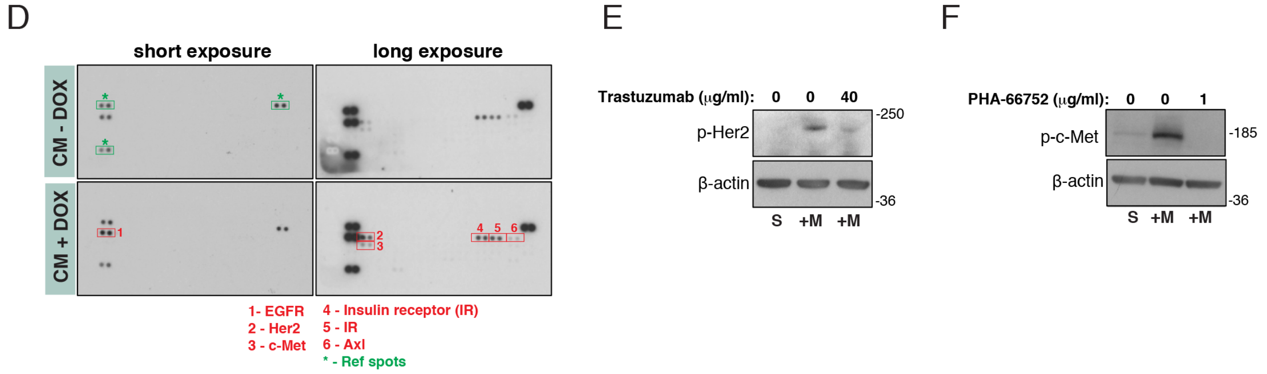

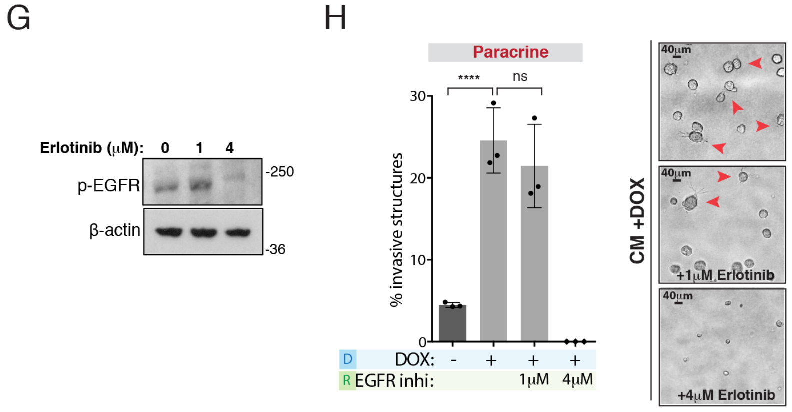

I

MCF10A.PLK4 - DOX					
sample	Initial cell #	Final cell #	Protein (mg/ml)	LDH (fold)	% invasion
1	$33.5e10^5$	$26e10^6$	16.58	1.0	3.36
2	$33.5e10^5$	$26e10^6$	16.01	1.0	3.24
3	$33.5e10^5$	$26e10^6$	17.86	0.9	4.79

MCF10A.PLK4 + DOX					
sample	Initial cell #	Final cell #	Protein (mg/ml)]	LDH (fold)	% invasion
1	$45.2e10^5$	$20e10^6$	19.11	1.1	15.91
2	$45.2e10^5$	$20e10^6$	19.24	1.2	17.45
3	$45.2e10^5$	$20e10^6$	20.63	1.3	17.67

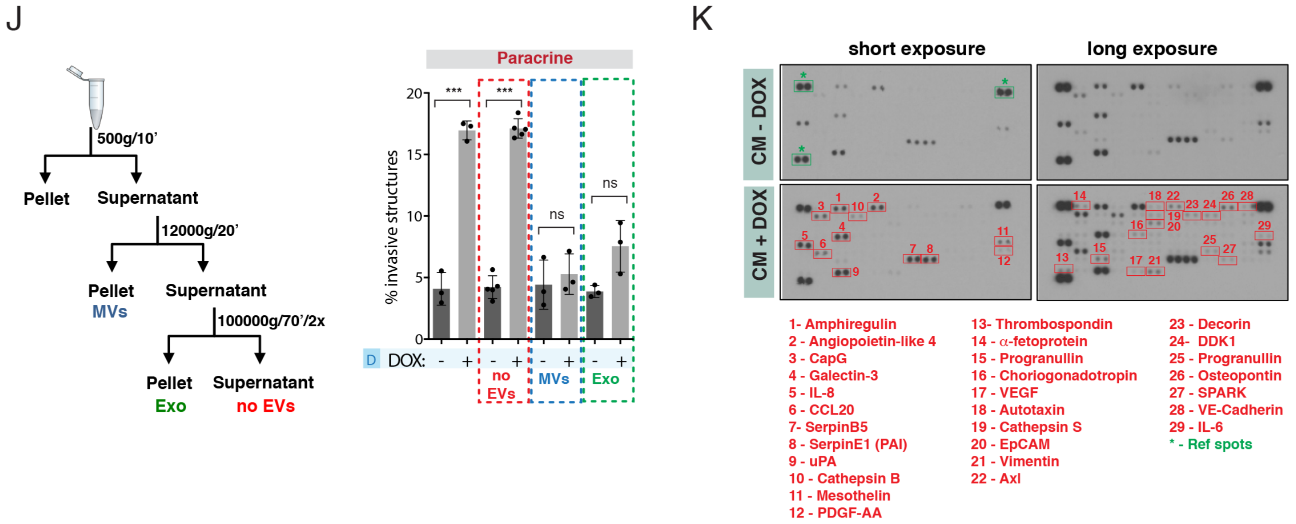

Figure S2. Proteins secreted by cells with extra centrosomes promote paracrine invasion via RTK activation. Related to Figure 2. (A) Levels of E-cadherin, N-cadherin and Vimentin in cells incubated with CM for 4 days. TGF-β treatment (5 ng/ml, 4 days) was used as positive control. (B) Schematic representation of Vivaspin filtration and tryspin incubation. (C) Quantification of invasive acini cells incubated with CM after Vivaspin filtration. P: proteins, M: metabolites. (D) phospho-RTK array obtained from cells incubated with CM. (E) phospho-Her2 levels in cells treated with 40 μg/ml of Trastuzumab (Her2 inhibitor) for 1 hr. S, serum starved; +M, plus fresh medium. (F) phospho-c-Met levels in cells treated with 1 μM PHA-66752 (c-Met inhibitor) for 1 hr. S, serum starved; +M, plus fresh medium. (G) phospho-EGFR levels in cells treated with different concentrations of Erlotinib (1 and 4 μM, 24 hrs). (H) Left, Quantification of invasive acini cells treated with EGFR inhibitor (Erlotinib, 1 and 4 μM) and incubated with CM. Right, Acinar structures. Red arrowheads indicate invasive acini. Scale bar 40μm. (I) Table summarising the conditions used to prepare CM for proteomic analysis. Cells with (+DOX) or without extra centrosomes (-DOX) were plated at different densities to ensure a similar cell number by the time of CM collection. Protein concentration and invasive capacity of the CM was assessed in all samples. (J) Left, Schematic representation of the ultracentrifugation method to isolate MVs and Exo. Right, Quantification of invasive acini in cells incubated with CM with and without MVs/Exo. (K) Human XL Oncology Array incubated with CM collected from cells with (CM+DOX) and without (CM-DOX) extra centrosomes. For all graphics error bars represent mean +/- SD from three independent experiments. ****$P < 0.001$, ****$P < 0.0001$,* ns not significant.

Figure S3

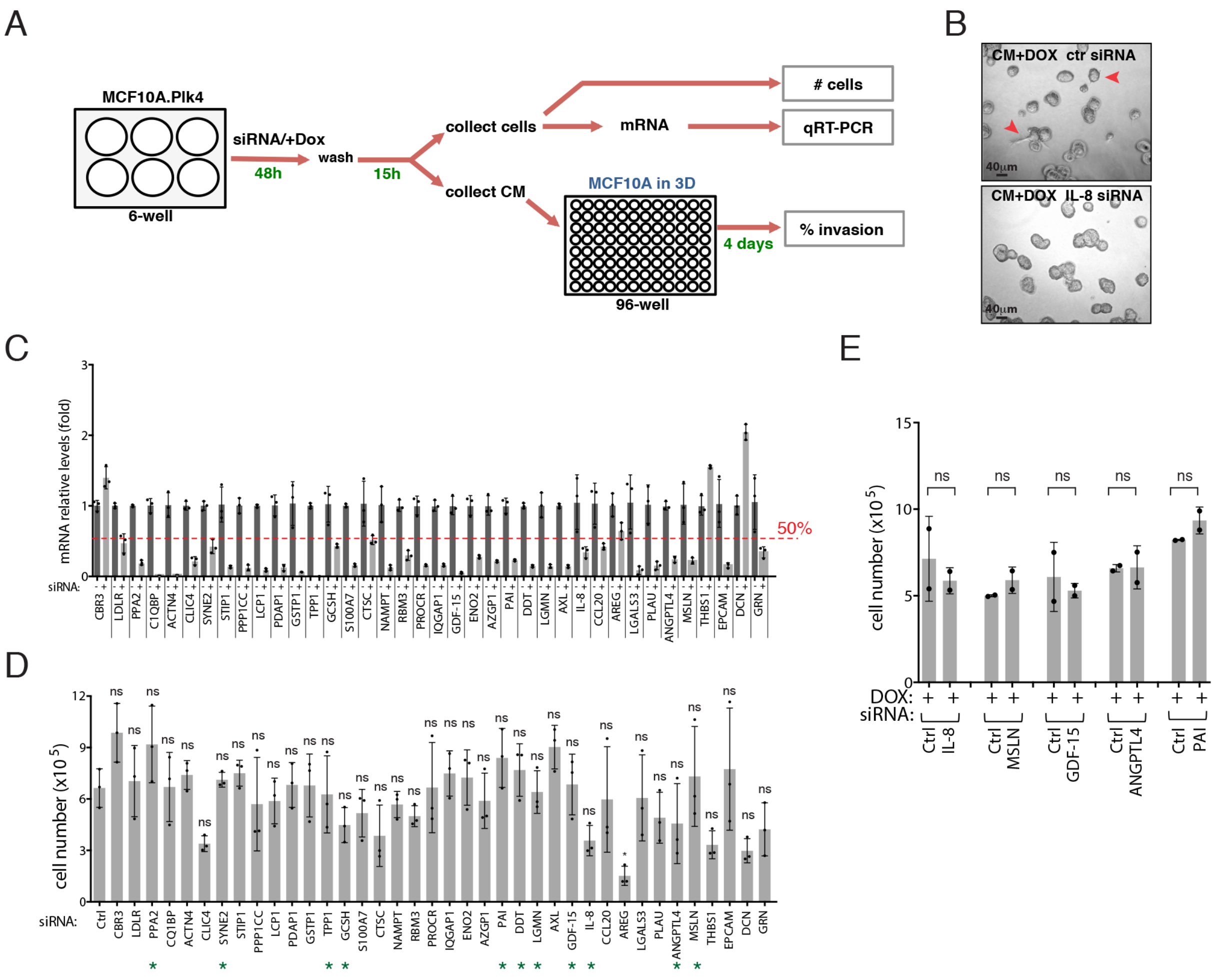

A
MCF10A.Plk4
6-well
siRNA/+Dox
48h
wash
15h
collect cells
collect CM
mRNA
cells
qRT-PCR
MCF10A in 3D
96-well
4 days
% invasion
B
CM+DOX ctr siRNA
CM+DOX IL-8 siRNA
40µm
C
mRNA relative levels (fold)
50%
siRNA:
CBR3 LDLR PPA2 C1QBP ACTN4 CLIC4 SYNE2 STIP1 PPP1CC LCP1 PDAP1 GSTP1 TPP1 GCSH S100A7 CTSC NAMPT RBM3 PROCR IQGAP1 GDF-15 ENO2 AZGP1 PAI DDT LGMN AXL IL-8 CCL20 AREG LGALS3 PLAU ANGPTL4 MSLN THBS1 EPCAM DCN GRN
D
cell number (x10^5)
siRNA:
Ctrl CBR3 LDLR PPA2 CQ1BP ACTN4 CLIC4 SYNE2 STIP1 PPP1CC LCP1 PDAP1 GSTP1 TPP1 GCSH S100A7 CTSC NAMPT RBM3 PROCR IQGAP1 ENO2 AZGP1 PAI DDT LGMN AXL GDF-15 IL-8 CCL20 AREG LGALS3 PLAU ANGPTL4 MSLN THBS1 EPCAM DCN GRN
ns
E
cell number (x10^5)
DOX:
siRNA:
Ctrl IL-8
Ctrl MSLN
Ctrl GDF-15
Ctrl ANGPTL4
Ctrl PAI
ns

Figure S3. siRNA screen identified secreted pro-invasive factors in MCF10A cells with extra centrosomes. Related to Figure 3 and Table S1. (A) Schematic representation of the siRNA screen set-up. (B) Acini images from 96-well plate siRNA screen. Scale bar 40μm. (C) mRNA levels for all the siRNA conditions to assess depletion efficiency. Red dashed line represents 50% reduction of mRNA levels. (D) Cell number upon siRNA treatment to assess the impact of the different conditions on cell proliferation/viability. Green asterisks depict the positive screen hits shown in Figure 3F. (E) Cell number in cells with extra centrosomes (+DOX) upon siRNA depletion of some of our positive hits. For all graphics error bars represent mean +/- SD from three independent experiments. *$P < 0.05$, ns not significant.

Figure S4

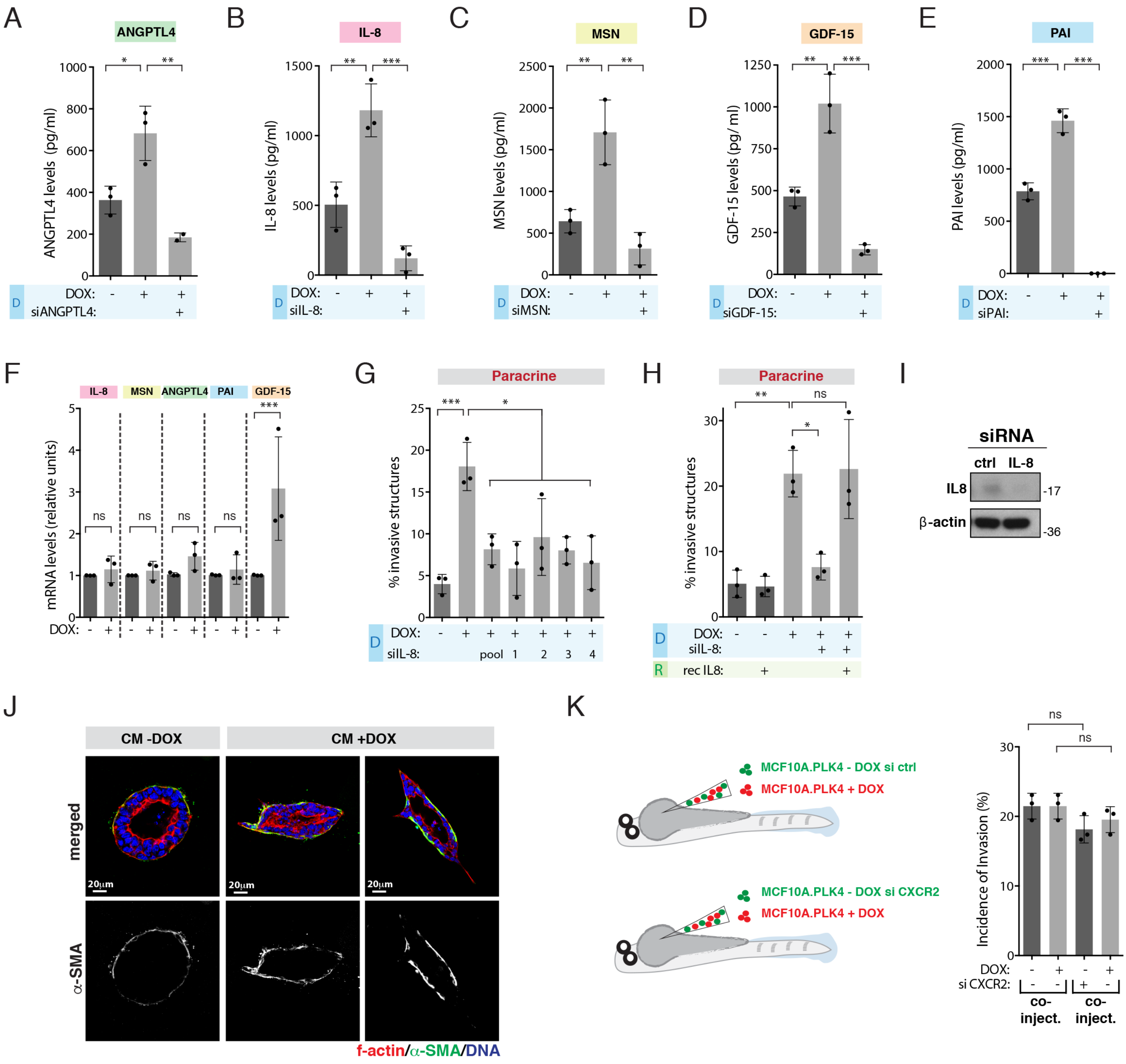

A
ANGPTL4
ANGPTL4 levels (pg/ml)
DOX:
siANGPTL4:
B
IL-8
IL-8 levels (pg/ml)
siIL-8:
C
MSN
MSN levels (pg/ml)
siMSN:
D
GDF-15
GDF-15 levels (pg/ ml)
siGDF-15:
E
PAI
PAI levels (pg/ml)
siPAI:
F
mRNA levels (relative units)
G
Paracrine
% invasive structures
pool
H
rec IL8:
I
siRNA
ctrl
IL-8
IL8
β-actin
J
CM -DOX
CM +DOX
merged
α-SMA
20µm
f-actin/α-SMA/DNA
K
MCF10A.PLK4 - DOX si ctrl
MCF10A.PLK4 + DOX
MCF10A.PLK4 - DOX si CXCR2
Incidence of Invasion (%)
si CXCR2:
co-inject.

Figure S4. Validation of the positive hits identified in the siRNA screen. Related to Figure 4. (A) Levels of secreted ANGPTL4. (B) Levels of secreted IL-8. (C) Levels of secreted MSN. (D) Levels of secreted GDF-15. (E) Levels of secreted PAI. (F) mRNA levels of the different pro-invasive factors after induction of centrosome amplification (48 hrs). (G) Quantification of invasive structures in acini incubated with CM collected from cells depleted of IL-8 (siRNA pool and individual sequences). (H) Quantification of invasive structures in acini incubated with CM collected from cells depleted of IL-8 and supplemented with recombinant IL-8 (0.5 μg/ml). (I) IL-8 levels after siRNA depletion. (J) Non-invasive and invasive mammary organoids from WT mice stained for f-actin (phalloidin, red), α-SMA (green) and DNA (blue). Scale bar 20μm. (K) Left, Schematic representation of the zebrafish co-injections. Right, Quantification of the incidence of invasion in the co-injection experiments (-DOX/+DOX) when CXCR2 is depleted in cells with normal centrosomes (-DOX). Number of injected fish co-injection control siRNA=71; co-injection CXCR2 siRNA=121. For all graphics error bars represent mean +/- SD from three independent experiments. *$P < 0.05$, **$P < 0.01$, ***$P < 0.001$, ns not significant.

Figure S5

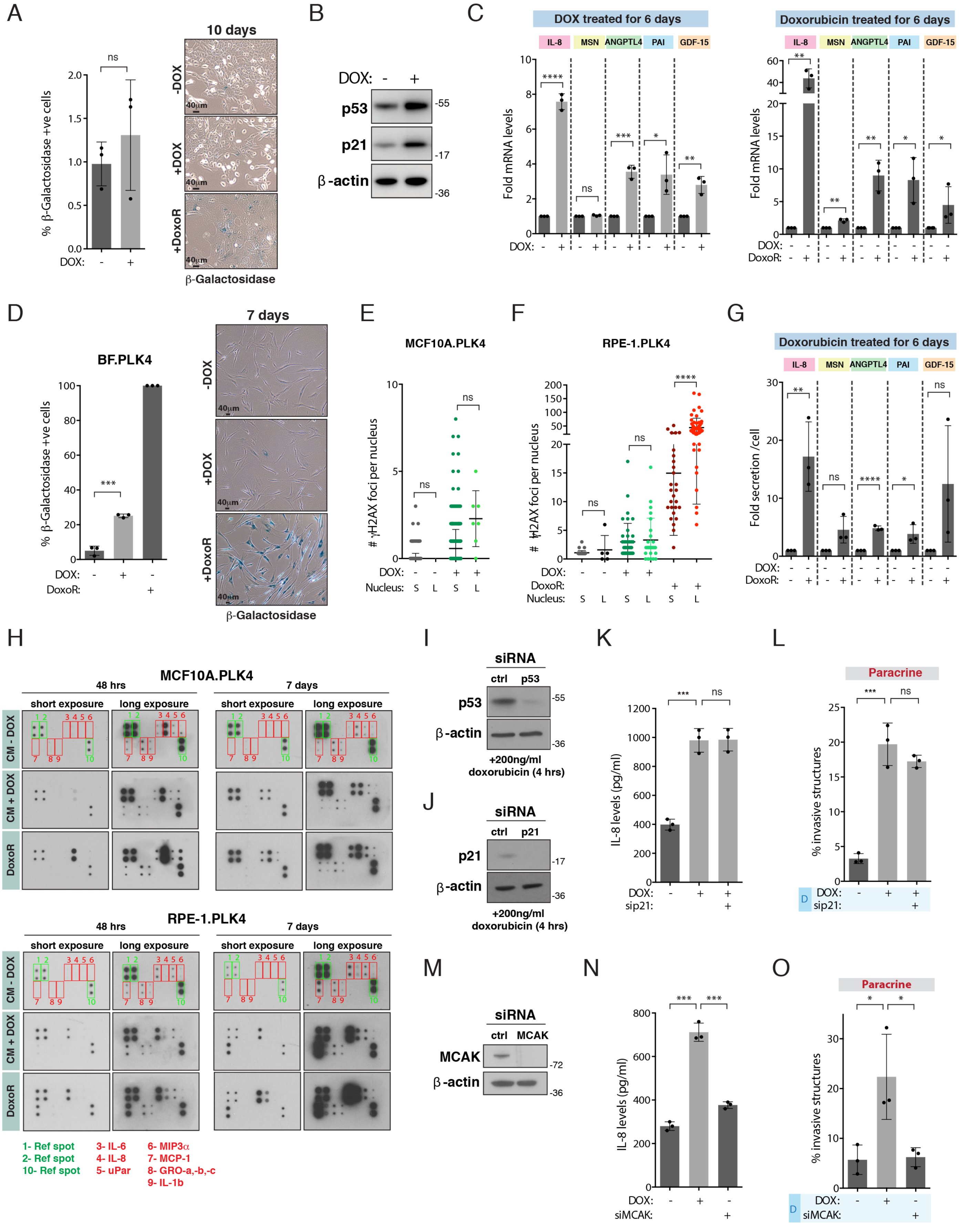

A
10 days
% β-Galactosidase +ve cells
DOX:
-DOX
+DOX
+DoxoR
β-Galactosidase
B
DOX:
p53
p21
β-actin
C
DOX treated for 6 days
Doxorubicin treated for 6 days
IL-8
MSN
ANGPTL4
PAI
GDF-15
Fold mRNA levels
DoxoR:
D
BF.PLK4
7 days
E
MCF10A.PLK4
γH2AX foci per nucleus
Nucleus:
F
RPE-1.PLK4
G
Fold secretion /cell
H
MCF10A.PLK4
48 hrs
7 days
short exposure
long exposure
CM - DOX
CM + DOX
DoxoR
RPE-1.PLK4
1- Ref spot
2- Ref spot
10- Ref spot
3- IL-6
4- IL-8
5- uPar
6- MIP3α
7- MCP-1
8- GRO-a,-b,-c
9- IL-1b
I
siRNA
ctrl
p53
+200ng/ml doxorubicin (4 hrs)
J
p21
K
IL-8 levels (pg/ml)
sip21:
L
Paracrine
% invasive structures
M
MCAK
N
siMCAK:
O

Figure S5. Secretion of pro-invasive factors in cells with extra centrosomes does not require a full senescence response and is not a consequence of aneuploidy. Related to Figure 5 and Table S1. (A) Left, Quantification of β-galactosidase positive MCF10A.PLK4 cells after induction of centrosome amplification for 10 days. Right, Images representing cells stained for β-galactosidase (blue). Scale bar 40μm. (B) Levels of p53 and p21 after induction of extra centrosomes (48 hrs). (C) mRNA levels of the different pro-invasive factors 6 days after induction of centrosome amplification (Left) or Doxorubicin treatment (Right). (D) Left, Quantification of β-galactosidase positive BF.PLK4 cells 7 days after induction of centrosome amplification or doxorubicin treatment (100 ng/ml). Right, Cells stained for β-galactosidase (blue). Scale bar 40μm. (E) Quantification of γH2AX foci in MCF10A cells with extra centrosomes after 7days. (F) Quantification of γH2AX foci in RPE-1 cells with extra centrosomes after 7days. DoxoR-induced senescent cells were used as positive control. L, large nuclei; S, small nuclei. Data as seen in Fig. 5f. (G) Quantification of secreted pro-invasive factors in senescent cells treated with DoxoR after 6 days. (H) SASP array incubated with CM collected from cells with (CM+DOX) and without (CM-DOX) extra centrosomes. DoxoR-induced senescent cells were used as positive control. (I) Levels of p53 after p53 siRNA. (J) Levels of p21 after p21 siRNA. (K) Levels of secreted IL-8 in cells depleted of p21. (L) Quantification of invasive structures in acini incubated with CM collected from cells depleted of p21. (M) Levels of MCAK after MCAK siRNA depletion. (N) Levels of secreted IL-8 in cells depleted of MCAK. (O) Quantification of invasive structures in acini incubated with CM collected from cells depleted of MCAK. For all graphics error bars represent mean +/- SD from three independent experiments. *$P < 0.05$, **$P < 0.01$, ***$P < 0.001$, ****$P < 0.0001$, ns not significant.

Figure S6

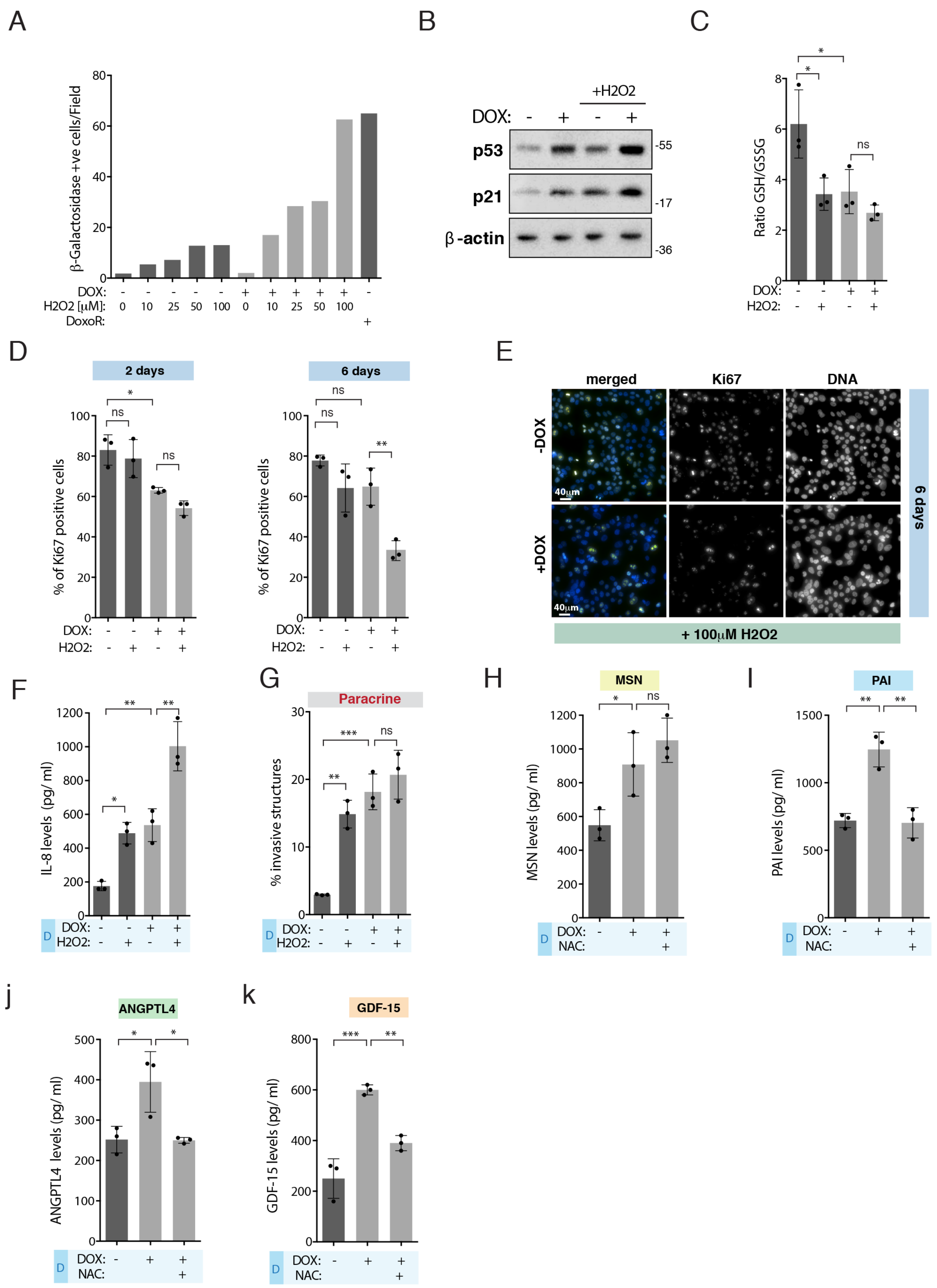

A
β-Galactosidase +ve cells/Field
DOX:
H2O2 [μM]:
DoxoR:
B
+H2O2
DOX:
p53
p21
β-actin
-55
-17
-36
C
Ratio GSH/GSSG
DOX:
H2O2:
D
2 days
6 days
% of Ki67 positive cells
E
merged
Ki67
DNA
-DOX
+DOX
40μm
6 days
+ 100μM H2O2
F
IL-8 levels (pg/ ml)
G
Paracrine
% invasive structures
H
MSN
MSN levels (pg/ ml)
NAC:
I
PAI
PAI levels (pg/ ml)
j
ANGPTL4
ANGPTL4 levels (pg/ ml)
k
GDF-15
GDF-15 levels (pg/ ml)

Figure S6. Increased ROS levels in cells with extra centrosomes promote secretion and paracrine invasion. Related to Figure 6 and Table S1. (A) Quantification of β-galactosidase positive MCF10A.PLK4 cells after 6 days, before and after 48 hrs treatment with DOX and treated with different doses of H2O2 for 48 hrs. (B) Levels of p53 and p21 in cells with (+DOX) and without (-DOX) extra centrosomes treated with H2O2 (100 μM). (C) Ratio of GSH/GSSG in cells after induction of extra centrosomes (48 hrs) and H2O2 (100 μM) treatment. (D) Quantification of Ki67 positive cells 2 and 6 days after induction of centrosome amplification and H2O2 (100 μM) treatment. (E) Cells stained for Ki67 (green) and DNA (blue). Scale bar 40μm. (F) Levels of secreted IL-8 in cells with (+DOX) and without (-DOX) extra centrosomes treated with H2O2 (100 μM, 48 hrs). (G) Quantification of invasive structures in acini incubated with CM collected from cells with (+DOX) and without (-DOX) extra centrosomes treated with H2O2 (100 μM). (H) Levels of secreted MSN after NAC treatment (5 mM, 48 hrs). (I) Levels of secreted PAI after NAC treatment (5 mM, 48 hrs). (J) Levels of secreted ANGPTL4 after NAC treatment (5 mM, 48 hrs). (K) Levels of secreted GDF-15 after NAC treatment (5 mM, 48 hrs). For all graphics error bars represent mean +/- SD from three independent experiments. *$P < 0.05$, **$P < 0.01$, ***$P < 0.001$, ns not significant.

Figure S7

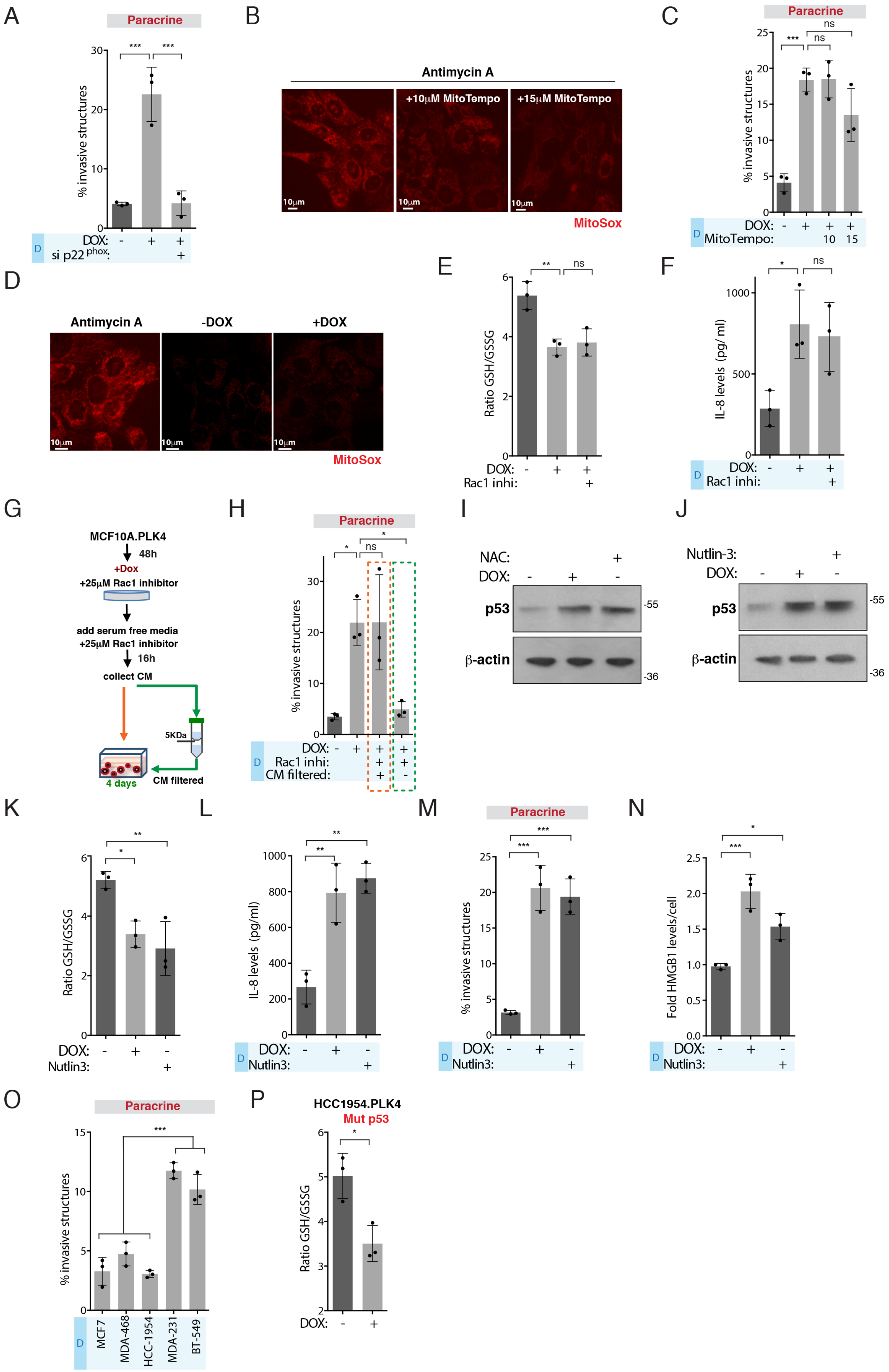
A
Paracrine
% invasive structures
***
***
DOX: - + +
si p22 phox: +
D
B
Antimycin A
+10µM MitoTempo
+15µM MitoTempo
10µm
MitoSox
C
Paracrine
% invasive structures
***
ns
ns
DOX: - + + +
MitoTempo: 10 15
D
Antimycin A
-DOX
+DOX
10µm
MitoSox
E
Ratio GSH/GSSG
**
ns
DOX: - + +
Rac1 inhi: +
F
IL-8 levels (pg/ ml)
*
ns
DOX: - + +
Rac1 inhi: +
G
MCF10A.PLK4
48h
+Dox
+25µM Rac1 inhibitor
add serum free media
+25µM Rac1 inhibitor
16h
collect CM
5KDa
4 days
CM filtered
H
Paracrine
% invasive structures
*
ns
*
DOX: - + + +
Rac1 inhi: + +
CM filtered: + -
I
NAC: +
DOX: - + -
p53
β-actin
-55
-36
J
Nutlin-3: +
DOX: - + -
p53
β-actin
-55
-36
K
Ratio GSH/GSSG
**
*
DOX: - + -
Nutlin3: +
L
IL-8 levels (pg/ml)
**
**
DOX: - + -
Nutlin3: +
M
Paracrine
% invasive structures
***
***
DOX: - + -
Nutlin3: +
N
Fold HMGB1 levels/cell
*
***
DOX: - + -
Nutlin3: +
O
Paracrine
% invasive structures
***
MCF7
MDA-468
HCC-1954
MDA-231
BT-549
P
HCC1954.PLK4
Mut p53
Ratio GSH/GSSG
*
DOX: - +

Figure S7. p53-mediated ROS production promotes secretion and paracrine invasion in cells with extra centrosomes. Related to Figure 7 and Table S1. (A) Quantification of invasive structures in acini incubated with CM collected from cells depleted of p22^{phox}. (B) Cells treated with Antimycin A (35 μM) to increase mitochondrial ROS and stained with fluorogenic dye Mitosox (red). MitoTempo was used to inhibit mitochondrial ROS (10 and 15 μM, 48 hrs). Scale bar 10μm. (C) Quantification of invasive structures in acini incubated with CM collected from cells with extra centrosomes treated with MitoTempo (10 and 15 μM, 48 hrs). (D) Cells were stained with MitoSox (red). Scale bar 10μm. (E) Ratio of GSH/GSSG in cells after induction of extra centrosomes (48 hrs) and treated with RAC1 inhibitor (NSC23766, 25 μM, 48 hrs). (F) Levels of secreted IL-8 in cells with (+DOX) and without (-DOX) extra centrosomes treated with RAC1 inhibitor (25 μM, 48 hrs). (G) Schematic representation of the experimental setting to remove RAC1 inhibitor from the CM before adding on 3D cultures. (H) Quantification of invasive structures in acini incubated with CM collected from cells treated with RAC1 inhibitor (25 μM) before and after removal of the drug (CM filtered). (I) p53 levels in cells with extra centrosomes (+DOX) and treated with NAC (5 mM, 48 hrs). (J) Western blot analysis of p53 levels in cells treated with Nutlin-3 (5 mM, 48 hrs). (K) Ratio of GSH/GSSG in cells treated with Nutlin-3 (5 mM, 48 hrs). (L) Levels of secreted IL-8 in cells treated with Nutlin-3 (5 mM, 48 hrs). (M) Quantification of invasive structures in acini incubated with CM collected from cells treated Nutlin-3 (5 mM, 48 hrs). (N) Levels of secreted HMGB1 in cells treated with Nutlin-3 (5 mM, 48 hrs). (O) Quantification of invasive structures in acini incubated with CM collected from breast cancer cell lines with low and high centrosome amplification. (P) Ratio of GSH/GSSG in HCC1954.PLK4 cells after induction of extra centrosomes (+DOX) for 48 hrs. For all graphics error bars represent mean +/- SD from three independent experiments. *$P < 0.05$, **$P < 0.01$, ***$P < 0.001$, ns not significant.

Table S1: Quantification of the percentage of extra centrosomes in the different conditions and cell lines. Related to Figures 1, 3, 5, 6, 7, S1, S3, S5, S6 and S7.

Cell line	Treatment(s)	% Centrosome amplification
**MCF10A.PLK4**	n/a*	12
**MCF10A.PLK4**	DOX	87
**MCF10A.PLK4-608**	DOX	11
**MCF10A.PLK4**	after DOX + siRNA ctr	42
**MCF10A.PLK4**	after DOX + siRNA SAS-6	18
**HaCat.PLK4**	n/a*	21
**HaCaT.PLK4**	DOX	73
**MCF10A**	RO-3306	58
**MCF10A.PLK4**	DOX + siRNA IL-8	66
**MCF10A.PLK4**	DOX + siRNA ANGPTL4	78
**MCF10A.PLK4**	DOX + siRNA GDF15	65
**MCF10A.PLK4**	DOX + siRNA PAI	71
**MCF10A.PLK4**	DOX + siRNA MSN	61
**MCF10A.PLK4**	DOX + siRNA p53	64
**MCF10A.PLK4**	DOX + siRNA p21	65
**MCF10A.PLK4**	DOX 6 days	39
**MCF10A.PLK4**	DOX + NAC	87
**MCF10A.PLK4**	DOX + Apocynin	93
**MCF10A**	Nutlin-3	8
**MCF10A.PLK4**	DOX + Rac inhibitor	82
**MCF10A.PLK4**	H2O2	8
**MCF10A.PLK4**	DOX + H2O2	46
**MCF7. PLk4**	n/a*	18
**MCF7. PLk4**	DOX	57
**HCC1954.PLK4**	n/a*	16
**HCC1954. PLK4**	DOX	82
**BF.PLK4**	n/a*	1
**BF.PLK4**	DOX	44
**RPE.PLK4**	n/a*	15
**RPE.PLK4**	DOX	80

* n/a: not applicable, no treatment performed

Supplementary Table 6: References for all siRNAs used. Related to Figures 3 and S3.

Gene symbol	Reference	Company
**CXCL8**	M-004756-00	Dharmacon
**CCL20**	M-007832-01	Dharmacon
**AREG**	M-017435-00	Dharmacon
**LGALS3**	M-010606-02	Dharmacon
**PLAU**	M-006000-02	Dharmacon
**ANGPTL4**	M-007807-02	Dharmacon
**MSLN**	M-006346-02	Dharmacon
**THBS1**	M-019743-01	Dharmacon
**EPCAM**	M-004568-03	Dharmacon
**DCN**	M-021491-00	Dharmacon
**GRN**	M-009285-02	Dharmacon
**SYNE2**	D-019259-01	Dharmacon
**CBR3**	L-008597-00	Dharmacon
**LDLR**	L-011073-00	Dharmacon
**PPA2**	L-012348-00	Dharmacon
**C1QBP**	L-011225-01	Dharmacon
**ACTN4**	L-011988-00	Dharmacon
**CLIC4**	L-013553-00	Dharmacon
**STIP1**	L-019802-00	Dharmacon
**PPP1CC**	L-006827-00	Dharmacon
**LCP1**	L-011716-00	Dharmacon
**PDAP1**	L-017675-00	Dharmacon
**GSTP1**	L-011179-00	Dharmacon
**TPP1**	L-005810-00	Dharmacon
**GCSH**	L-017907-00	Dharmacon
**S100A7**	L-011769-02	Dharmacon
**CTSC**	L-005835-00	Dharmacon
**NAMPT**	L-004581-00	Dharmacon
**RBM3**	L-018969-01	Dharmacon
**PROCR**	L-017326-00	Dharmacon
**IQGAP1**	L-004694-00	Dharmacon
**GDF15**	L-019875-00	Dharmacon
**ENO2**	L-009777-00	Dharmacon
**AZGP1**	L-012567-01	Dharmacon
**SERPINE1**	L-019376-01	Dharmacon
**DDT**	L-012201-01	Dharmacon
**LGMN**	L-005924-00	Dharmacon
**AXL**	L-003104-00	Dharmacon
**CEP192**	L-032250-01	Dharmacon
**P21**	L-003471-00	Dharmacon
**SAS6**	L-019156-01	Dharmacon
**P53**	L-003329-00	Dharmacon
**CXCR2**	L-005647-00	Dharmacon
**CYBA**	L-011020-02	Dharmacon
**CONTROL**	1027310	Qiagen

Supplementary Table 7: Primer sequences used in the RT-qPCR. Related to Figures S3, S4 and S5.

Gene symbol	Primer Forward 5'-3'	Primer Reverse 5'-3'
**GAPDH**	TTAAAAGCAGCCCTGGTGAC	CTCTGCTCCTCCTGTTCGAC
**CXCL8**	GTTTTTGAAGAGGGCTGAG	TTTGCTTGAAGTTTCACTGG
**CCL20**	TATATTGTGCGTCTCCTCAG	GCTATGTCCAATTCCATTCC
**AREG**	AAAGAAAGAAAAAGGGAGGC	CATTTGCATGTTACTGCTTC
**LGALS3**	AGATTTCCAAAGAGGGAATG	AAGTGCAAACAATGACTCTC
**PLAU**	GAAAACCTCATCCTACACAAG	ATTCTCTTTTCCAAAGCCAG
**ANGPTL4**	AGGCAGAGTGGACTATTTG	CCTCCATCTGAGGTCATC
**MSLN**	GACGTCCTAAAGCATAAACTG	TCATCTTGAGGAAGAGGTAG
**THBS1**	GTGACTGAAGAGAACAAAGAG	CAGCTATCAACAGTCCATTC
**EPCAM**	GTCTGAGAAGGCTGAGATAAAG	CTTCAAAGATGTCTTCGTCC
**DCN**	TTCACGCATTGATTCTTGTC	GCTGATTCTTGGACAGATAAAG
**GRN**	GACCTGATCCAGAGTAAGTG	CATGTCACATTTCACATCCC
**SYNE2**	GAGAAGATAGAAGAAGCACTC	TCTTATAGGTTTTCTGCTGC
**CBR3**	AGTGAGGTGCTAGTTTCCAAGG	CTGCACATTCTTGTGGACCC
**LDLR**	GAGGACAAAGTATTTTGGACAG	GTAGGTTTTCAGCCAACAAG
**PPA2**	CTGGAAGCTACTCTTAATTGG	GCCTTGTTTTTGAATTCTCC
**C1QBP**	AACATTAACAACAGCATCCC	TCATCCTCTGGATAATGACAG
**ACTN4**	AGTATGACAAGCTGAGGAAG	CTGAAAAGGCATGGTAGAAG
**CLIC4**	CCCAGAATCAAATCATGCTG	TCAATTTCATCAGGGAGAGG
**STIP1**	AACGAGTGTTTTCAGAAAGG	TATAACCCTTGATGAAGGTCG
**PPP1CC**	GAGGTTTATCACCAGATCTTC	CAGCCTAAGACATGTTTATCG
**LCP1**	AAGCTCTGATTGCTCTTTTG	GAAGTTGCCAATTTTGTTGC
**PDAP1**	AAGATGACTACCAGCAAAAG	CTGCTTCTCAATCTCTTCTC
**GSTP1**	GCAAATACATCTCCCTCATC	GTCTCAAAAGGCTTCAGTTG
**TPP1**	AATAACCTGACCCTAGAGAATG	CCACATAGTGATGAAACTCAG
**GCSH**	TTGTTATGAAGATGGTTGGC	TCTAAGTCTTCTATCCACCAC
**S100A7**	TTAGTGCCTGTGACAAAAAG	GTAGTCTGTGGCTATGTCTC
**CTSC**	AACAAACTGGCCATGAAC	ATAAAGACTCCAGAAGGGAC
**NAMPT**	CTAATGGCCTTGGGATTAAC	TCCAGTGTAACAAAATTCCC
**RBM3**	AGTGGCAGGTATTATGACAG	TCTGCCATTATAGTCTCTGG
**PROCR**	CATATGAAGTCTTTGGAGGC	CATATGGAAGTCTTTGGAGGC
**IQGAP1**	ATGCCTTTGACATCATTGAC	TCTAGGTTTCTGGTAGGACTG
**GDF15**	CGAAGACTCCAGATTCCG	ACTTCTGGCGTGAGTATC
**ENO2**	ATGTCCATAGAGAAGATCTGG	GACACCTTTGCCTAAGTAAC
**AZGP1**	ACAGAAATCACAGTCAATGG	TCCAAGTCTACTCAAGACAG
**SERPINE1**	ATCCACAGCTGTCATAGTC	CACTTGGCCCATGAAAAG
**DDT**	CCCACTTCTTTGAGTTTCTC	ATCTCTCTGGAAGAAGCAG
**LGMN**	ACTATGATGAGAAGAGGTCC	GGTGGAGATTGTTTTGTTTC
**AXL**	CATGAAACATGGAGACCTAC	ATCTCTTGGTACTCAGATACTC
