# Supplementary material for: Oxidative Stress in Cells with Extra Centrosomes Drives Non-Cell-Autonomous Invasion
Source: Dev Cell. 2018 Nov 19;47(4):409–424.e9. doi: 10.1016/j.devcel.2018.10.026 (PMC6251975; doi:10.1016/j.devcel.2018.10.026)
Supplement: Document S2. Article plus Supplemental Information [file mmc10.pdf]

Article

Developmental Cell

Oxidative Stress in Cells with Extra Centrosomes Drives Non-Cell-Autonomous Invasion

Graphical Abstract

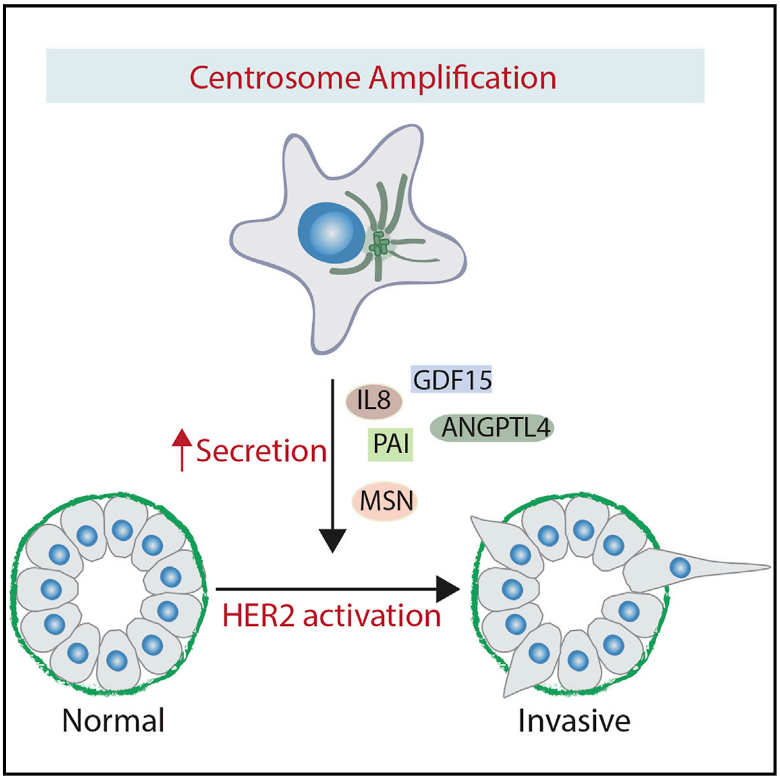

Highlights

- Centrosome amplification induces non-cell-autonomous invasion
- Oxidative stress leads to the secretion of pro-invasive factors
- IL-8 is a major ROS-mediated secreted factor important for paracrine invasion
- High ROS can lead to a senescence-like phenotype in cells with extra centrosomes

Authors

Teresa Arnandis, Pedro Monteiro, Sophie D. Adams, ..., Ilaria Malanchi, Pedro R. Cutillas, Susana A. Godinho

Correspondence

s.godinho@qmul.ac.uk

In Brief

Arnandis et al. uncovered a non-cell-autonomous function for centrosome amplification in cancer. Cells with extra centrosomes induce paracrine invasion via secretion of pro-invasive factors. Altered secretion is partly regulated by elevated reactive oxygen species in cells with extra centrosomes. This work highlights far-reaching consequences of centrosome amplification in cancer.

Arnandis et al., 2018, Developmental Cell *47*, 409–424
November 19, 2018 © 2018 The Authors. Published by Elsevier Inc.
https://doi.org/10.1016/j.devcel.2018.10.026

CellPress

Developmental Cell
Article

CellPress

Oxidative Stress in Cells with Extra Centrosomes Drives Non-Cell-Autonomous Invasion

Teresa Arnandis,[1] Pedro Monteiro,[1] Sophie D. Adams,[1] Victoria Louise Bridgeman,[2] Vinothini Rajeeve,[3] Emanuela Gadaleta,[1] Jacek Marzec,[1] Claude Chelala,[1] Ilaria Malanchi,[2] Pedro R. Cutillas,[3] and Susana A. Godinho[1,4,*]
[1]Barts Cancer Institute, Queen Mary University of London, Charterhouse Square, London EC1M 6BQ, UK
[2]Tumour Host Interaction Laboratory, The Francis Crick Institute, 1 Midland Rd, London NW1 1AT, UK
[3]Integrative Cell Signalling and Proteomics, Barts Cancer Institute, Queen Mary University of London, Charterhouse Square, London EC1M 6BQ, UK
[4]Lead Contact
*Correspondence: s.godinho@qmul.ac.uk
https://doi.org/10.1016/j.devcel.2018.10.026

SUMMARY

Centrosomal abnormalities, in particular centrosome amplification, are recurrent features of human tumors. Enforced centrosome amplification *in vivo* plays a role in tumor initiation and progression. However, centrosome amplification occurs only in a subset of cancer cells, and thus, partly due to this heterogeneity, the contribution of centrosome amplification to tumors is unknown. Here, we show that supernumerary centrosomes induce a paracrine-signaling axis via the secretion of proteins, including interleukin-8 (IL-8), which leads to non-cell-autonomous invasion in 3D mammary organoids and zebrafish models. This extra centrosomes-associated secretory phenotype (ECASP) promotes invasion of human mammary cells via HER2 signaling activation. Further, we demonstrate that centrosome amplification induces an early oxidative stress response via increased NOX-generated reactive oxygen species (ROS), which in turn mediates secretion of pro-invasive factors. The discovery that cells with extra centrosomes can manipulate the surrounding cells highlights unexpected and far-reaching consequences of these abnormalities in cancer.

INTRODUCTION

The centrosome is the principal microtubule (MT) organizing center in animal cells and consists of a pair of centrioles surrounded by the pericentriolar material (PCM) (Bettencourt-Dias and Glover, 2007). The centrosome is duplicated once per cell cycle during S-phase to ensure that at the onset of mitosis, cells have two centrosomes. The importance of the centrosome cycle is emphasized by its tight regulation and conservation throughout evolution (Nigg and Stearns, 2011; Werner et al., 2017). However, cancer cells often display centrosomal abnormalities; in particular, centrosome amplification has been extensively characterized in both solid and hematological malignancies (Chan, 2011; Marteil et al., 2018; Zyss and Gergely, 2009). Mounting evidence indicates that extra centrosomes are not bystanders of tumor progression and can directly impact tumorigenesis by generating aneuploidy and promoting the acquisition of invasive traits (Ganem et al., 2009; Godinho et al., 2014). Furthermore, recently developed mouse models demonstrated that induction of centrosome amplification, via transient Polo-like kinase 4 (PLK4) overexpression, not only accelerates tumorigenesis in the absence of the tumor suppressor p53 (Coelho et al., 2015; Sercin et al., 2016) but also promotes tumor formation in p53-proficient mice (Levine et al., 2017). Therefore, centrosome amplification can play a role in the initiation and progression of tumors.

Intriguingly, the presence of supernumerary centrosomes comes with a cost (Rhys and Godinho, 2017). Cells with extra centrosomes divide slower and require efficient mechanisms that enable the formation of a ‘‘pseudo-bipolar’’ spindle during mitosis (e.g., centrosome clustering) to prevent catastrophic multipolar division (Basto et al., 2008; Ganem et al., 2009; Kwon et al., 2008; Rhys et al., 2018). Furthermore, in cells with intact tumor suppressors, centrosome amplification induces p53-mediated cell-cycle arrest (Fava et al., 2017; Holland et al., 2012). Thus, while it is predictable that cells with extra centrosomes undergo negative selection *in vitro* (Krzywicka-Racka and Sluder, 2011; Mittal et al., 2017), it is perhaps counterintuitive that *in vivo* tumors maintain ‘‘less-fit’’ cells carrying centrosomal abnormalities. This is particularly surprising given tumor heterogeneity, where most human tumors display high genetic and phenotypic diversity (McGranahan and Swanton, 2017), including heterogeneous centrosome numbers (Chan, 2011). Thus, why are cells with extra centrosomes not outcompeted during tumor evolution? It is becoming clear that tumor evolution cannot be merely explained by positive selection of the fittest clones (McGranahan and Swanton, 2017; Tabassum and Polyak, 2015). In fact, widespread intratumor heterogeneity (ITH) challenges the idea that the dominant subclone solely drives tumor phenotypes in a cell autonomous manner (McGranahan and Swanton, 2017). Using mouse xenograft models, Polyak and colleagues found that a subclone overexpressing interleukin (IL)-11 acted as a non-cell-autonomous driver of tumor growth and was essential to maintain ITH by promoting the growth of less-fit clones (Marusyk et al., 2014). Here, we set out to investigate whether cells with extra centrosomes play non-cell-autonomous roles that could benefit the surrounding cells and explain their maintenance in tumors.

This is an open access article under the CC BY license (http://creativecommons.org/licenses/by/4.0/).

RESULTS

Centrosome Amplification Induces Paracrine Invasion

To investigate whether the presence of extra centrosomes promotes non-cell-autonomous functions, we took advantage of non-transformed cells to avoid additional effects caused by cancer mutations. To do so, conditioned media (CM) was collected from our previously established human mammary epithelial cell line MCF10A.PLK4 (donor [D] cells) where centrosome amplification is driven by transient induction of PLK4 upon doxycycline (DOX) treatment (Godinho et al., 2014) (Figure S1A). CM collected at 16, 24, and 36 hr from donor cells was added on top of recipient (R) MCF10A cells grown in 3D cultures, which form acinar structures (Figure 1A). Strikingly, CM collected from cells with extra centrosomes (CM+DOX) was able to induce a robust invasive phenotype (~20%), characterized by the formation of actin-rich invasive protrusions capable of degrading the basement membrane (Figures 1B and S1B). We previously found that centrosome amplification was sufficient to drive invasion in a cell-autonomous manner (Godinho et al., 2014); however, paracrine invasion was not a consequence of increased centrosome numbers in the recipient cells (Figure S1A). Live cell imaging of 3D acini treated with CM shows how these invasive protrusions enable collective migration through the extracellular matrix (ECM) (Videos S1 and S2) and allow cells to invade the surrounding environment (Figure S1C; Videos S3 and S4). When compared with invasive protrusions induced directly by extra centrosomes, protrusions induced by the CM+DOX appeared morphologically distinct, containing increased percentages of nuclei (~23% as opposed to ~5%) (Figure S1D). Moreover, when added on top of cells with extra centrosomes (+DOX), CM+DOX further increased invasion, with many of the structures displaying a more severe and abnormal invasive phenotype (Figure S1E), suggesting an additive effect. CM collected from human keratinocytes with extra centrosomes (HaCat.PLK4+DOX) also induced paracrine invasion of MCF10A cells, showing that this phenotype is not cell-type dependent (Figure S1F and Table S1).

We established that secretion of pro-invasive factors is a direct consequence of centrosome amplification and not due to unspecific effects of PLK4 overexpression or DOX treatment. First, depletion of SAS-6, a centrosomal protein essential for centrosome duplication (Rodrigues-Martins et al., 2007; Strnad et al., 2007), in cells upon overexpression of PLK4 (+DOX) prevents centrosome amplification and paracrine invasion (Figures 1C and 1D). Second, CM collected from cells treated with DOX to induce the expression of a truncated PLK4 mutant ($PLK4^{1–608}$) that does not induce centrosome amplification does not induce paracrine invasion (Figure 1E) (Guderian et al., 2010). Third, depletion of SAS-6 after centrosome amplification leads to loss of extra centrosomes and blocks the ability of these cells to induce paracrine invasion, demonstrating that this phenotype can be reversed by loss of extra centrosomes (Figures S1G and S1H; Table S1). Finally, increased paracrine invasion was observed in cells where centrosome amplification was generated by prolonged CDK1 inhibition, in the absence of DOX treatment (Figure S1I and Table S1) (Loncarek et al., 2010).

We validated the ability of cells with extra centrosome to promote paracrine invasion in primary mouse mammary organoids that better recapitulate the architecture of the mammary gland. In this system, both myoepithelial cells (expressing α-SMA) and luminal cells become invasive upon treatment with CM from cells with extra centrosomes (Figures 1F and 1G). This type of invasion, in particular the collective invasive strands, resembles what has been described for invasive tumor organoids in 3D cultures (Cheung et al., 2013). Branching morphogenesis can also be observed in these conditions, but we did not quantify this phenotype as invasion (Figure S1J).

To test if in the context of heterogeneous cell populations, cells with extra centrosomes could promote invasion of surrounding cells, we took advantage of the zebrafish model where co-injection of differentially labeled cells was performed. While injection of MCF10A cells with normal centrosome number (−DOX) does not induce an invasive phenotype, induction of centrosome amplification (+DOX) is sufficient to promote an invasive behavior *in vivo*, scored as the percentage of fish with cells that invaded into the tail (~20%) (Figures 1H–1J). However, when control cells and cells with extra centrosomes are co-injected, non-invasive control cells become invasive (~15%) (Figures 1H–1J). These results support a non-cell-autonomous role for centrosome amplification *in vivo*.

Induction of Paracrine Invasion Is Mediated by RTK Signaling

To investigate the mechanisms by which CM from cells with extra centrosomes promoted invasion, we first tested if CM+DOX induced epithelial to mesenchymal transition (EMT) in MCF10A cells. We found that cells treated with CM+DOX did not undergo EMT, as assessed by the expression of epithelial (E-cadherin) and mesenchymal (N-cadherin and Vimentin) markers (Figure S2A). Next, we tested if the pro-invasive factors secreted by cells with extra centrosomes were permanently making them invasive by pre-treating MCF10A cells with CM for 48 hr before plating in 3D cultures (CM OFF) (Figure 2A). We found that pre-treatment with CM+DOX was not sufficient to induce an invasive phenotype (Figure 2A). Thus, signaling activation via secreted molecules is likely inducing paracrine invasion. To uncover the origin of the secreted pro-invasive factors, we filtered the CM using Vivaspin columns with a 5 kDa cutoff to separate larger molecules (e.g., proteins) from small molecules (e.g., metabolites). We found that only the larger fraction (>5 kDa) was able to induce invasion (Figures S2B and S2C). Treatment of the CM+DOX with trypsin-coated beads completely abolished the invasive phenotype, further supporting that it is a protein-mediated phenotype (Figures 2B and S2B).

To dissect the signaling pathways activated by CM+DOX, we performed a phospho-receptor tyrosine kinase (RTK) array in MCF10A cells treated with CM collected from control (CM−DOX) and cells with extra centrosomes (CM+DOX). Analyses of the phospho-RTK array revealed that EGFR, HER2 (ErbB2), and c-Met (HGFR) signaling were increased in cells treated with CM+DOX, although only HER2 was significantly increased (Figures 2C and S2D). Addition of the HER2 inhibitor (Trastuzumab), but not the c-Met inhibitor (PHA-66752) to the recipient cells, prevented paracrine invasion induced by the CM+DOX, without affecting acinar growth (Figures 2D, S2E, and S2F). Our data demonstrate that activation of HER2 signaling in the recipient cells drives non-cell-autonomous invasion. Because EGF

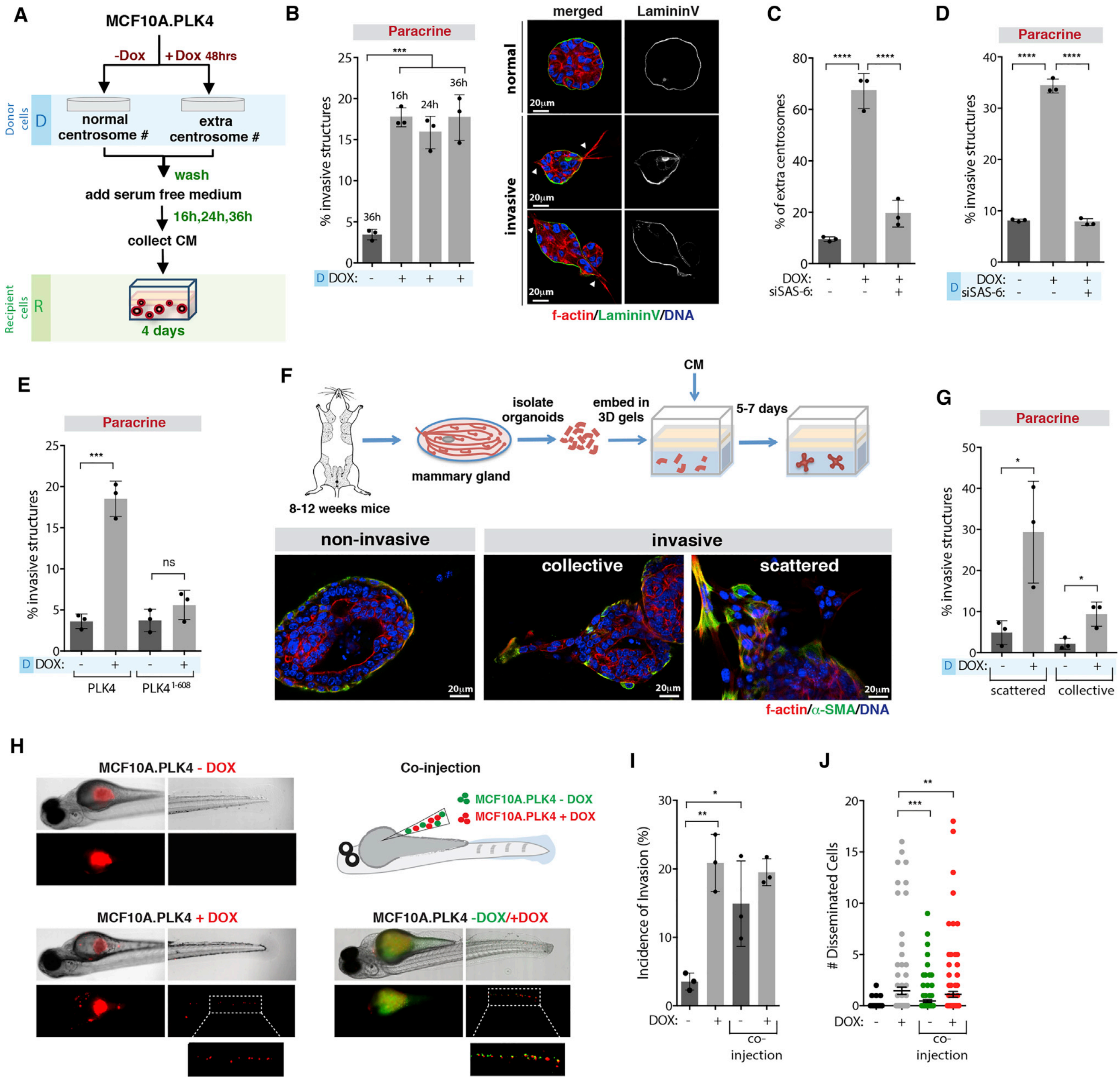

Figure 1. Centrosome Amplification Induces Paracrine Invasion

(A) Experimental flowchart.

(B) Left, quantification of invasive structures. Right, normal and invasive 3D acini. White arrowheads indicate invasive protrusions. Scale bar: 20 μM.

(C) Quantification of centrosome amplification.

(D) Quantification of invasive structures.

(E) Quantification of invasive structures.

(F) Top, schematic representation of mammary organoids isolation and growth. Bottom, non-invasive and invasive mammary organoids. Scale bar: 20 μM.

(G) Quantification of invasive organoids.

(H) Images of zebrafish injected with cells with (+DOX) or without (−DOX) extra centrosomes (left) or co-injected +DOX/−DOX (right).

(I) Incidence of invasive cells in zebrafish embryos. Number of injected fish −DOX = 121; +DOX = 103; and co-injection +/−DOX = 116.

(J) Number of disseminated cells in the zebrafish tail. Error bars represent mean ± SEM.

For all graphics, error bars represent mean ± SD from three independent experiments. *$p < 0.05$, **$p < 0.01$, ***$p < 0.001$, ****$p < 0.0001$; ns not significant. See also Figure S1; Videos S1, S2, S3, and S4; Table S1.

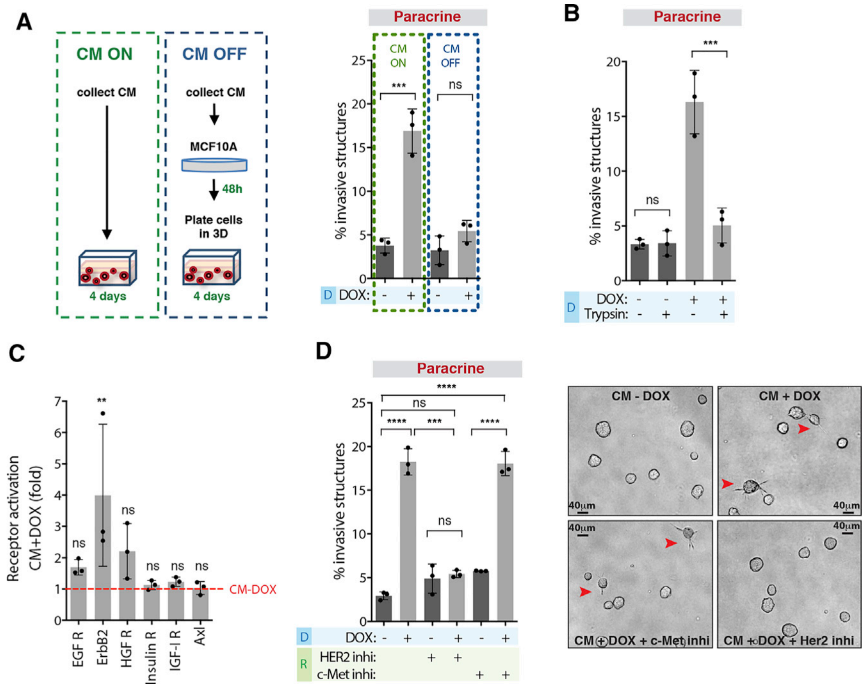

Figure 2. Induction of Paracrine Invasion Is Mediated by RTK Signaling

(A) Left, schematic representation of the different CM treatments. Right, quantification of invasive structures.

(B) Quantification of invasive structures.

(C) Fold increase in RTK phosphorylation in MCF10A cells after incubation with CM+DOX.

(D) Left, quantification of invasive structures with or without HER2 (Trastuzumab, 40 μg/mL) and c-Met (PHA-66752, 1 μM) inhibitors. Right, acinar structures. Red arrowheads indicate invasive acini. Scale bar: 40 μM.

For all graphics, error bars represent mean ± SD from three independent experiments. $^{**}p < 0.01$, $^{***}p < 0.001$, $^{****}p < 0.0001$; ns not significant.

See also Figure S2.

signaling is essential for MCF10A proliferation, it remains undetermined if EGFR is important for the invasive phenotype observed (Figures S2G and S2H).

Secretome Analysis Reveals Differential Protein Secretion in Cells with Amplified Centrosomes

To identify the secreted proteins important for paracrine invasion, we performed label-free mass spectrometry on the CM−DOX and CM+DOX (Figure 3A). CM was collected 16 hr after incubation with serum-free medium to prevent cell death, as assessed by the presence of the enzyme lactate dehydrogenase (LDH) in the media (Figure S2I). Proteomic analyses uncovered changes (log2-fold > 1.5) in the secretomes of cells with and without centrosome amplification (Figure 3B and Table S2), demonstrating the existence of an extra centrosomes-associated secretory phenotype (ECASP). Similar to other secretomic studies (Acosta et al., 2013), qualification of proteins differentially present in the CM revealed that approximately 25% of those are assigned to the extracellular compartment (Figure 3C). Further analyses of this compartment suggested that many of the identified secreted proteins have been previously associated with extracellular vesicles, particularly exosomes (Exos) (Figure 3C). However, while fractions enriched for microvesicles (MVs) or Exos did not significantly promote invasion, CM depleted of extracellular vesicles (MVs and Exos) retained the potential to induce invasion (Figure S2J). To complement our secretomic analyses and exclude proteins associated with MVs and Exos, we performed a quantitative membrane-based protein array (Human XL Oncology Array) of the collected CM (Figures 3A and 3D). Short and long exposures of the membranes revealed proteins significantly secreted by cells with amplified centrosome number (Figures 3D and S2K). Ingenuity pathway analysis (IPA) of the secreted proteins identified by mass spectrometry and/or protein array demonstrated that many have been previously linked to cancer invasion and migration (Figure 3E and Table S3). From those, we selected 38 proteins based on fold change and function in cancer (Table S4) and performed a small-scale small interfering RNA (siRNA) screen in cells with extra centrosomes to identify the secreted pro-invasive proteins (Figures S3A and S3B). qRT-PCR to assess knockdown efficiency indicated that 3 out of 38 proteins were not depleted, and therefore they were not pursued further (Figure S3C). We identified 11 proteins that upon depletion decreased paracrine invasion by at least 1 standard deviation (SD) of the siRNA control condition (∼5%) (Figure 3F, green circles). The decrease in paracrine invasion was independent of cell viability and proliferation rates (Figure S3D). We further validated some of the hits that have been previously associated with invasion using independent siRNA pool sets: Interleukin-8 (IL-8), Mesothelin (MSLN), Angiopoietin-like protein 4 (ANGPTL4), SerpinE1 (PAI), and Growth-Factor Differentiation 15 (GDF-15) (Figures 3G and S3E) and confirmed their increased secretion by ELISA (Figures S4A–S4E). Apart from GDF-15, increased secretion of these factors at 48 hr cannot be explained by increased mRNA levels (Figure S4F). One of our top hits, IL-8, also known as C-X-C motif ligand 8 (CXCL8), is a pro-inflammatory chemokine with known roles in promoting cancer cell invasion (Waugh and Wilson, 2008). IL-8 signaling has also been shown to induce transactivation of HER2 (Singh et al., 2013), which is important for centrosome amplification-induced paracrine invasion (Figure 2D). Deconvoluted siRNA pools for IL-8 confirmed its role in paracrine invasion (Figure S4G). Furthermore, while recombinant IL-8 was not sufficient to induce invasion when added to CM−DOX, it fully restored the invasive capacity of the CM+DOX collected from cells depleted of IL-8 (Figures S4H and S4I). Taken together, our data suggest that paracrine invasion induced by extra centrosomes is likely promoted by a combination of secreted factors, with IL-8 playing a crucial role.

Secreted IL-8 Is Crucial for Paracrine Invasion through HER2 Activation

To investigate the mechanisms by which IL-8 could be promoting paracrine invasion, we inhibited IL-8 G-protein-coupled receptors

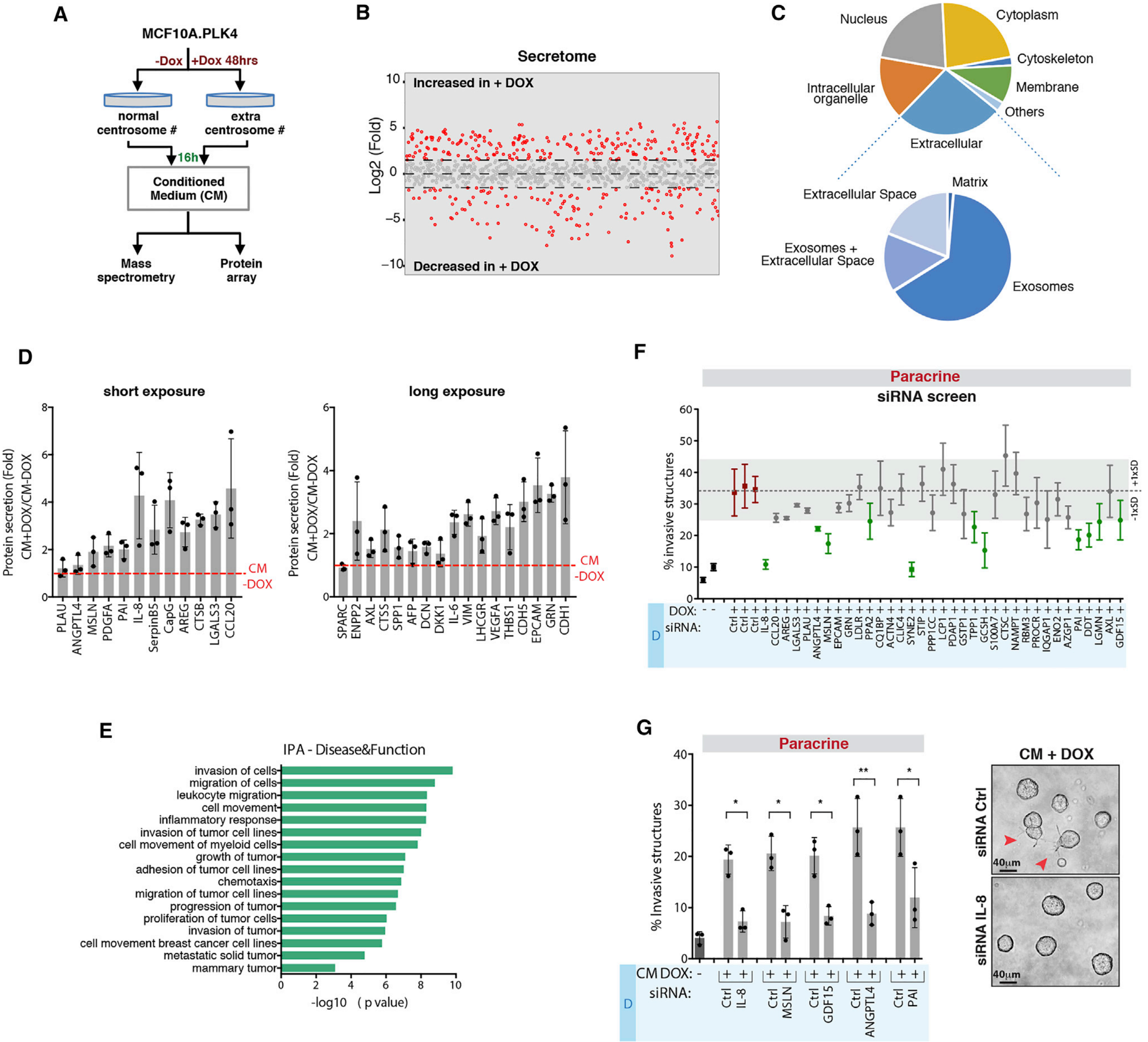

Figure 3. Secretome Analysis Reveals Differential Protein Secretion in Cells with Amplified Centrosomes

(A) Experimental flowchart.

(B) Log_2-fold changes in protein abundance in the CM of cells with extra centrosomes (+DOX). Red circles indicate changes >1.5-fold difference.

(C) Pie charts represent the cellular localization of the proteins increased in CM+DOX. See STAR Methods for details.

(D) Fold change of secreted proteins in CM+DOX using protein array.

(E) IPA classification of the extracellular secreted proteins identified by mass spectrometry and protein arrays.

(F) Quantification of invasive structures after siRNA depletion.

(G) Left, validation of specific positive hits identified in (F). Right, acinar structures. Red arrowheads indicate invasive acini. For all graphics, error bars represent mean ± SD from three independent experiments. *$p < 0.05$, **$p < 0.01$. Scale bar: 40 μM.

See also Figure S3 and Table S1.

CXCR1 and CXCR2 in the recipient cells using specific inhibitors (Casilli et al., 2005; Chao et al., 2007). While the CXCR1/2 allosteric inhibitor Reparixin only partially prevented invasion, inhibition of CXCR1/2 with SCH563705 (potently inhibits CXCR2) abolished paracrine invasion without affecting 3D growth (Figure 4A). This was confirmed by siRNA depletion of CXCR2 in the recipient cells (Figures 4B and 4C). Importantly, CXCR2 depletion in cells with extra centrosomes did not prevent direct invasion, although we did observe a consistent decrease in the invasive phenotype (Figure 4C). These results further support that the pathways that underline direct and paracrine invasion are distinct. We confirmed the importance of IL-8 signaling in this process using mouse mammary organoids. Although mice do not express IL-8, they express CXCR2 that binds to human IL-8 (Singer and Sansonetti,

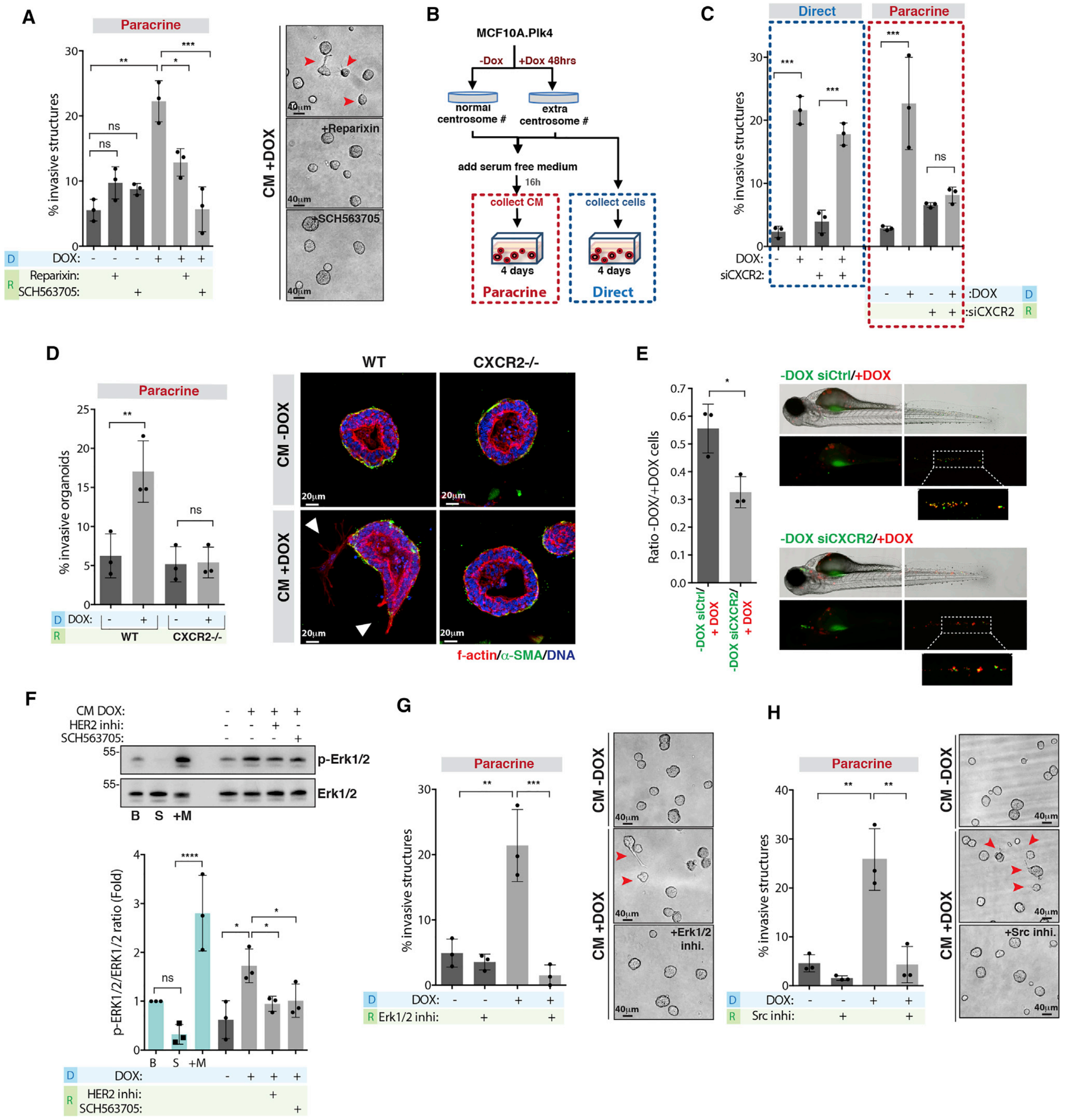

Figure 4. Secreted IL-8 Is Crucial for Paracrine Invasion through Her2 Activation

(A) Left, quantification of invasive structures with and without the CXCR1/2 inhibitors Reparixin (100 nM) and SCH563705 (100 nM). Right, acinar structures. Red arrowheads indicate invasive acini. Scale bar: 40 μM.

(B) Experimental flowchart.

(C) Quantification of invasive structures upon CXCR2 depletion in cells with extra centrosomes (direct) or incubated with CM+DOX (paracrine).

(D) Left, quantification of invasive mammary organoids from WT or CXCR2$^{-/-}$ mice. Right, non-invasive and invasive mammary organoids. Scale bar: 20 μM.

(E) Left, ratio of disseminated cells in co-injection experiments. Right, zebrafish embryos co-injected with cells with (+DOX, red) and without centrosome amplification (−DOX, green). Number of injected fish co-injection control siRNA = 71; co-injection CXCR2 siRNA = 121.

(F) Top, levels of p-Erk1/2 and total Erk1/2 in cells. Bottom, ratio of phospho/total Erk1/2. B, basal conditions; S, serum starved cells; +M, serum starved cells after incubation with fresh medium.

(G) Left, quantification of invasive structures with or without Erk1/2 inhibitor (PD98059, 20 μM). Right, acinar structures. Red arrowheads indicate invasive acini. Scale bar: 40 μM.

(legend continued on next page)

2004). Mammary organoids generated from mice knockout for CXCR2 (CXCR2$^{-/-}$) did not show increased invasion when treated with CM+DOX (Figures 4D and S4J). Co-injection experiments in zebrafish demonstrated that, although depletion of CXCR2 in control MCF10A cells (−DOX) did not abolish paracrine invasion (Figure S4K), it significantly decreased the number of control cells that co-invaded with cells with extra centrosomes (+DOX), indicating that IL-8 signaling plays an important role in paracrine invasion *in vivo* (Figure 4E).

Because CM+DOX activates HER2 (Figure 2C), we decided to test if HER2 activation requires IL-8 signaling. To do so, we assessed Erk1/2 phosphorylation, as a consequence of HER2 activation, in recipient cells treated with CM+DOX. We found that, when compared to CM−DOX, CM+DOX induces a 2-fold increase in Erk1/2 activation. Moreover, pre-treating the recipient cells with HER2 or CXCR1/2 inhibitors significantly decreased Erk1/2 activation (Figure 4F). Similar to HER2 inhibition, chemical inhibition of Erk1/2 and Src, important for HER2 transactivation mediated by IL-8 (Singh et al., 2013), in the recipient cells also prevented invasion induced by CM+DOX (Figures 4G and 4H). Taken together, our results show that Erk1/2 activation is important for paracrine invasion and requires activation of the IL-8 receptor.

Centrosome Amplification Induces an Early Stress Response that Leads to Altered Secretion

Altered protein secretion in senescent cells, known as the senescence-associated secretory phenotype (SASP), was previously shown to lead to IL-8 secretion (Coppe et al., 2008). Early secretory phenotype observed in cells with extra centrosomes is unlikely to be a consequence of senescence since it is induced very early (48 hr after induction of extra centrosomes), and high levels of proliferating cells can be observed even after 6 days, as measured by ki67 staining (Figure 5A). However, the percentage of dividing cells with extra centrosomes is lower at day 6 (Table S1). Thus, it is possible that this early response to centrosome amplification could culminate in a senescence phenotype. To test this idea, we performed β-galactosidase staining in cells 6 and 10 days after induction of centrosome amplification. In contrast to cells treated with the DNA-damage-inducing drug Doxorubicin (DoxoR) (Bolesta et al., 2012), centrosome amplification in MCF10A cells was not sufficient to drive a strong senescence phenotype (Figures 5B and S5A). However, our data suggest that these cells display senescence-like features. First, centrosome amplification increases p53 and p21 protein levels (Holland et al., 2012) (Figure S5B), which is also observed in senescent cells (Wiley and Campisi, 2016). Second, the comparison of the mRNA levels of the identified pro-invasive factors at day 6 revealed a similar trend between senescent cells (DoxoR treated) and cells with extra centrosomes, although overall senescent cells exhibit a stronger response (Figure S5C). In addition, senescent cells exhibit a similar paracrine invasive behavior as centrosome amplification, although they exhibit higher levels of secreted pro-invasive factors (Figures 5C, 5D, and S5G). This partial senescence-like response could be a consequence of the lack of p16 in MCF10A cells (Brenner and Aldaz, 1995), since both p16 and p53 are important mediators of senescence (Wiley and Campisi, 2016). To test if centrosome amplification was sufficient to induce senescence in cells with intact p16 and p53, we used human retinal pigment epithelium (RPE-1) and human primary breast fibroblast (BF) cell lines. Although RPE-1 were negative for β-galactosidase staining, centrosome amplification was sufficient to induce an enlarged cell morphology phenotype in ~30% of the cells consistent with senescence after 7 days (Figure 5E and Table S1). Similar results were observed in BF cells as scored by β-galactosidase staining (Figure S5D and Table S1). Notably, the levels of β-galactosidase were lower than that of BF cells treated with DoxoR, suggesting that extra centrosomes might elicit a different or less strong senescent response. We also assessed double-stranded DNA (dsDNA) breaks, as measured by γH2AX foci, and found that while there is a significant increase in cells with extra centrosomes, only 1.7% and 11.3% of MCF10A and RPE-1 cells, respectively, show more than 5 DNA damage foci (Figure 5F). This contrasts with DoxoR-induced senescent cells where ~99% of cells have more than 5 DNA damage foci (Figure 5F). Furthermore, while enlarged nuclei in senescent cells induced by DoxoR correlate with increased DNA damage foci, the same was not observed in cells with extra centrosomes (Figures S5E and S5F). To further understand the association of ECASP with senescence, we compared the secretion of well-established SASP components (Coppe et al., 2008), including IL-8 (our positive control), IL-6, uPar, MIP-3α, MCP-1, GRO -a, -b, -c, and IL-1β, in cells with extra centrosomes or treated with DoxoR over time (48 hr and 7 days). At 48 hr, we did not observe a strong SASP, and only secretion of IL-6 and IL-8 was observed (Figures 5G and 5H). At 7 days, DoxoR-induced senescent cells displayed a stronger SASP than cells with extra centrosomes in both MCF10A and RPE-1 cells (Figures 5G and 5H). In RPE-1 cells, centrosome amplification and DoxoR-treated cells show a similar pattern of secreted SASP components. By contrast, MCF10A cells with extra centrosomes only show increased secretion of IL-6 and IL-8 even after 7 days (Figures 5G, 5H, and S5H). Taken together, these results suggest that centrosome amplification can promote senescence and SASP in some cells. Similar results on a SASP induced by centrosome amplification have also been observed by others (D. Pellman, personal communication). Importantly, the early secretory phenotype we observed at 48 hr does not require cells to become senescent, as these still display high levels of proliferation. Instead, we hypothesized that this early secretory phenotype is caused by an early stress response, which could lead to senescence. Further supporting this idea, secretion of HMGB1, which is secreted very early after a senescence-induced stimulus and before the development of SASP (Davalos et al., 2013), is also observed 48 hr after

(H) Left, quantification of invasive structures with and without Src inhibitor (PP2, 5 μM). Right, acinar structures. Red arrowheads indicate invasive acini. Scale bar: 40 μM.

For all graphics, error bars represent mean ± SD from three independent experiments. *p < 0.05, **p < 0.01, ***p < 0.001; ns not significant.

See also Figure S4.

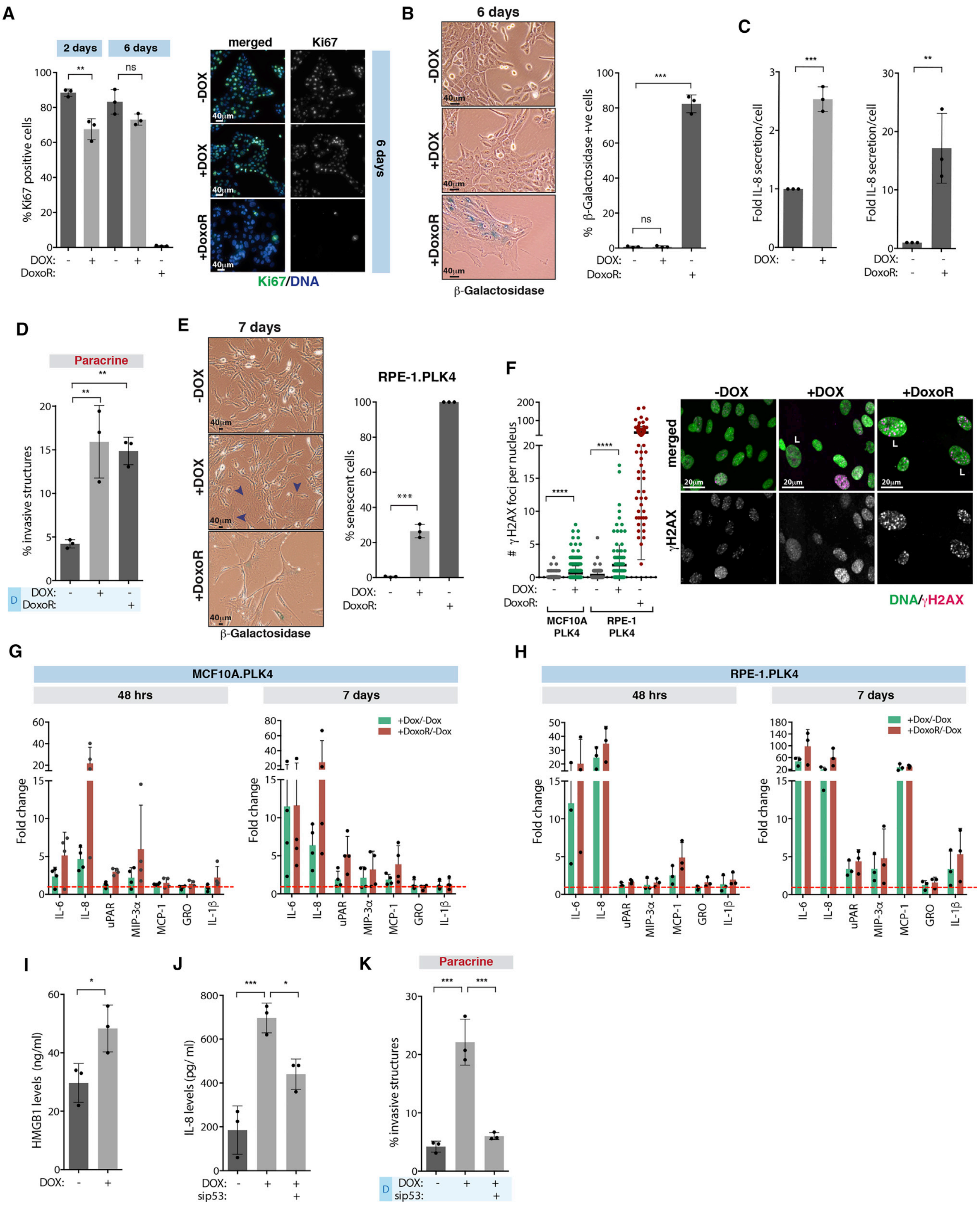

Figure 5. Centrosome Amplification Induces an Early Stress Response that Leads to Altered Secretion

(A) Left, quantification of Ki67 positive cells. Right, cells stained for Ki67. Scale bar: 40 μM.

(B) Left, cells stained for β-galactosidase (blue). Right, quantification of β-galactosidase positive cells after 6 days. Scale bar: 40 μM.

(legend continued on next page)

induction of extra centrosomes (Figure 5I). This stress response requires p53, since short-term depletion of p53 abolished paracrine invasion and decreased IL-8 secretion (Figures 5J, 5K, and S5I). This is not due to cell-cycle arrest mediated by p53 since p21 depletion did not prevent IL-8 secretion and paracrine invasion (Figures S5J–S5L). Taken together, our results suggest that a stress response downstream of extra centrosomes alters secretion that in some conditions can develop into a SASP.

Increased ROS Levels in Cells with Extra Centrosomes Drive Secretion

Recent work showed that induction of highly abnormal karyotypes leads to senescence and secretion of pro-inflammatory cytokines (Santaguida et al., 2017). In our conditions, induction of centrosome amplification for 48 hr leads to low levels of chromosome missegregation (Godinho et al., 2014). We found that depletion of MCAK, which induces similar levels of aneuploidy to centrosome amplification (Godinho et al., 2014), does not lead to paracrine invasion and IL-8 secretion (Figures S5M–S5O). One common denominator between IL-8 secretion and senescence is increased reactive oxygen species (ROS) levels (Fialkow et al., 2007; Gorrini et al., 2013). The levels of ROS can dictate the cellular response: high levels lead to senescence and apoptosis, whereas milder levels are associated with tumorigenesis (Gorrini et al., 2013). Thus, we postulated that increased levels of ROS in cells with extra centrosomes could alter secretion and, depending on the cellular context, also induce senescence. To test this, we measured ROS levels in cells with extra centrosomes using the fluorogenic dye DCFDA. We found that induction of centrosome amplification induces a 1.5-fold increase in ROS, which can be prevented by treating cells with the antioxidant N-acetyl cysteine (NAC). A similar response was observed in cells treated with DoxoR for 3 hr (Figure 6A). This was further confirmed by quantifying the levels of reduced Glutathione, which is decreased in response to ROS (~2-fold) (Figure 6B). Induction of extra centrosomes leads to the nuclear accumulation of the nuclear factor erythroid 2-related factor 2 (NRF2), a transcription factor that is stabilized and translocates into the nucleus in response to ROS (Figure 6C) (Gorrini et al., 2013). Consistently, gene expression analysis of MCF10A.PLK4 with extra centrosomes revealed an early transcriptional response involving the overexpression of genes that control intracellular ROS, some of which are downstream of NRF2 (Figure 6D and Table S5).

We uncovered that ROS is a key player in the paracrine invasive phenotype mediated by extra centrosomes. Treatment of cells with NAC prevented both IL-8 secretion and paracrine invasion, without affecting the levels of centrosome amplification (Figures 6E and 6F; Table S1). To test whether the lack of a classical senescent phenotype in MCF10A cells could be overcome by further increasing ROS, we treated cells with different doses of the ROS-inducing agent H2O2. Indeed, H2O2 concentrations that did not induce senescence in control cells were able to induce a stronger p53 response and promote senescence in cells with extra centrosomes (Figures 6G and S6A–S6C). This is consistent with increased DNA damage foci and a decrease in cell proliferation and dividing cells with extra centrosomes (Figures 6H, S6D, and S6E; Table S1). Moreover, 100 μM H2O2 was sufficient to promote IL-8 secretion and paracrine invasion, further supporting a role for ROS in this process (Figures S6F and S6G). Interestingly, reducing ROS levels with NAC also affected the secretion of ANGPTL4, GDF-15, and PAI, while MSN was still secreted at similar levels (Figures S6H–S6K). Because NAC prevents paracrine invasion, we tested if a combination of ROS-mediated secreted factors was sufficient to drive invasion. In contrast to the addition of recombinant IL-8, ANGPTL4, or GDF-15 alone, the combination of these three factors to CM−DOX was sufficient to promote paracrine invasion (Figure 6I), suggesting that ROS-mediated secretion plays crucial roles in non-cell-autonomous invasion.

Intracellular ROS can originate in the cytoplasm or mitochondria (Block and Gorin, 2012; Murphy, 2009). Inhibition of NADPH oxidases (NOXs), which drives cytoplasmic ROS, with apocynin decreased ROS levels, prevented IL-8 secretion and paracrine invasion in cells with extra centrosomes (Figures 6J–6L). This was confirmed by siRNA depletion of p22^{phox}, a common subunit of the NOX1-4 complexes (Figure S7A). By contrast, inhibition of mitochondrial ROS with MitoTempo did not prevent paracrine invasion, and centrosome amplification did not increase mitochondrial ROS, as assessed using the fluorogenic dye MitoSox (Figures S7B–S7D).

RAC1 activity is increased in cells with extra centrosomes (Godinho et al., 2014) and can activate NOX to increase ROS production (Block and Gorin, 2012); therefore, we tested whether RAC1 activity was important for ROS generation. To do so, we used a RAC1 inhibitor that we have previously shown to prevent increased RAC1 activity in response to centrosome amplification (Godinho et al., 2014). In this condition, RAC1 inhibition did not prevent increased ROS, and as a consequence, these cells were able to secrete IL-8 (Figures S7E and S7F). As RAC1 is important for the formation of invasive protrusions, we tested if the CM+DOX collected from D cells treated with RAC1 inhibitor still had the potential to induce invasion, after

(C) Relative IL-8 secretion (fold, ng/cell) in cells with extra centrosomes (Left) or treated with DoxoR (Right).

(D) Quantification of invasive structures.

(E) Left, cells stained for β-galactosidase (blue). Right, quantification of senescence in RPE-1.PLK4 cells. Note that senescence was assessed by enlarged morphology (purple arrowheads). Scale bar: 40 μM.

(F) Left, quantification of γH2AX foci. Right, cells were stained for γH2AX. L, large nuclei. Number of cells MCF10A.PLK4 −DOX = 469; +DOX = 466; and RPE-1.PLK4 −DOX = 155; +DOX = 115; +DoxoR = 84. Scale bar: 20 μM.

(G and H) (G) Fold change of secreted SASP components in MCF10A and (H) RPE-1 cells.

(I) HMGB1 secretion after 48 hr.

(J) IL-8 secretion after p53 depletion (48 hr).

(K) Quantification of invasive structures.

Graphic in (G) represents 4 independent experiments; for all other graphics, error bars represent mean ± SD from three independent experiments. *$p < 0.05$, **$p < 0.01$, ***$p < 0.001$, ****$p < 0.0001$; ns, not significant.

See also Figure S5 and Table S1.

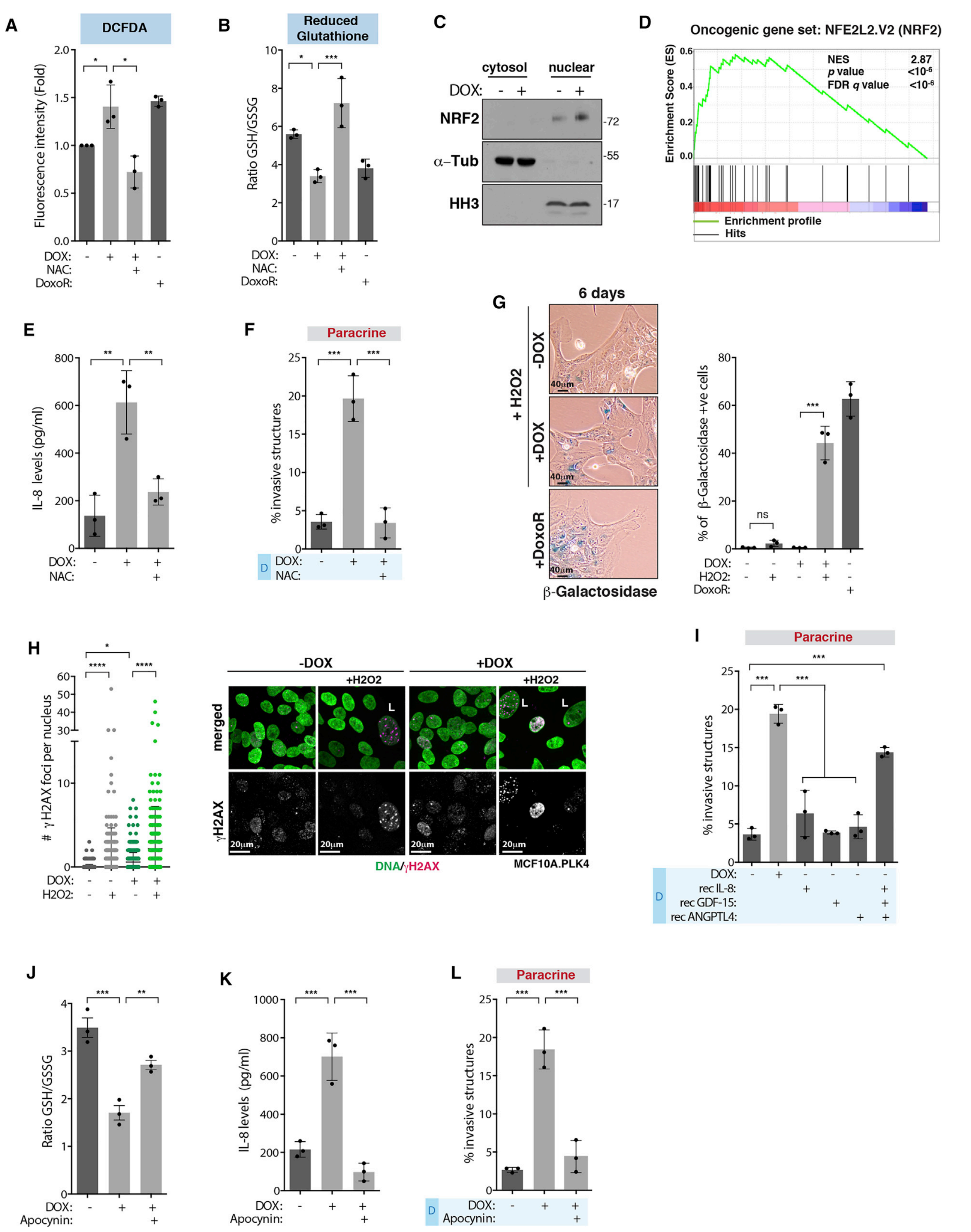

(legend on next page)

using Vivaspin columns to remove the inhibitor from the CM (Figure S7G). Whereas CM+DOX containing the RAC1 inhibitor prevented the formation of invasive protrusions, after removal of the inhibitor CM+DOX still retained the ability to induce paracrine invasion (Figure S7H). Thus, increased RAC1 activity is not necessary for the early secretion of pro-invasive factors.

We postulated that increased ROS could be a consequence of p53 stabilization since p53 plays important roles in redox homeostasis (Liu et al., 2008), and we found it to be important for paracrine invasion (Figure 5K). The role of p53 in modulating cellular ROS is complex. While p53 can be downstream of high levels of ROS, p53 activation has also been shown to promote ROS, which is important to drive senescence or apoptosis (Vigneron and Vousden, 2010). NAC treatment did not block p53 stabilization in cells with extra centrosomes, suggesting that p53 activation is not mediated by ROS (Figure S7I). Furthermore, we found that induction of p53 stabilization for 48 hr using Nutlin-3, an inhibitor of the p53 negative regulator MDM2, is sufficient to induce ROS, IL-8 secretion, and paracrine invasion in normal MCF10A cells, independently of centrosome amplification (Figures S7J–S7M; Table S1). Nutlin-3 treatment also induces HMGB1 secretion, similarly to centrosome amplification (Figure S7N). Altogether, our results suggest that p53-mediated ROS production leads to an early secretory response in cells with extra centrosomes that promotes non-cell-autonomous invasion.

Centrosome Amplification in Breast Cancer Mediates Paracrine Invasion and Is Associated with IL-8 Secretion

To establish the relevance of our findings in cancer, we next tested whether CM collected from cells with supernumerary centrosomes could induce invasion in organoid cultures of primary cells from mouse tumors derived from Polyomavirus middle T oncogene (PyMT) (Ogura et al., 2017). We found that CM+DOX was sufficient to increase invasion of tumor organoids after 4 and 7 days' incubation. This was accompanied by an increase in the number of tubular structures particularly at day 7 (Figures 7A and 7B). Tubular structures incubated with CM+DOX display an increase in the area and number of branches. This was not due to increased proliferation since round organoids do not show these differences (Figures 7C and 7D). Branches are locally regulated invasive epithelial buds essential for formation of the mammary gland (Sternlicht, 2006); thus, the increase in tubular organoids and branching further supports a role for CM+DOX in promoting invasion. The formation of branches is controlled via paracrine interactions and requires HER2 signaling (Sternlicht, 2006), similar to paracrine-induced invasion by cells with extra centrosomes.

We found that CM collected from breast cancer cell lines with extra centrosomes (MDA-231 and BT-549) was also able to increase paracrine invasion of MCF10A cells, suggesting that this effect is not restricted to non-transformed cells. This further emphasizes that non-cell-autonomous invasion induced by centrosome amplification does not require cells to be senescent (Figures 7E and S7O). Using the Cancer Cell Line Encyclopedia (CCLE) database, we found a correlation between centrosome amplification and the expression of the secreted pro-invasive factors, excluding GDF-15, in a panel of breast cancer cell lines (Figures 7E and 7F), which we previously characterized for centrosome amplification (Rhys et al., 2018). Importantly, induction of centrosome amplification in the breast cancer cells lines MCF-7 (p53 WT) and HCC-1954 (p53 mutant), which are of luminal and basal origin, respectively, was also sufficient to induce paracrine invasion in the recipient MCF10A cells (Figure 7G and Table S1). Thus, centrosome amplification-induced paracrine invasion is independent of the breast cancer subtype and can occur in the presence of at least some p53 mutations. In the case of HCC-1954 harboring the missense c.488A>G p53 mutation, increased ROS was observed upon induction of extra centrosomes (Figure S7P). Therefore, the presence of extra centrosomes elicits a similar non-cell-autonomous phenotype in non-transformed and cancer cells. Taken together, we propose that a stress response downstream of extra centrosomes leads to the secretion of proteins, partly mediated by increased ROS, that promote an invasive behavior in the surrounding cells (Figure 7H).

DISCUSSION

Our study establishes that the impact of extra centrosomes in tumors goes beyond altering the biology of cells that carry this abnormality by promoting non-cell-autonomous invasion. Structural centrosomal abnormalities have been recently shown to play non-cell-autonomous roles by changing the biomechanical properties of the epithelium leading to the budding of mitotic cells (Ganier et al., 2018). Here, we show that non-cell-autonomous invasion promoted by centrosome amplification is mediated by a secretory response that culminates with the secretion of multiple pro-invasive factors, including IL-8, ANGPTL4, PAI,

Figure 6. Increased ROS Levels in Cells with Extra Centrosomes Drive Secretion

(A and B) (A) Levels of intracellular ROS using DCFDA or (B) by measuring the ratio of GSH/GSSG (48 hr).

(C) NRF2 protein levels in the cytosolic and nuclear fractions.

(D) Gene expression profile of cells with extra centrosomes (48 hr) compared to an NRF2 (NFE2L2)-induced gene-set signature.

(E) IL-8 secretion in after NAC treatment.

(F) Quantification of invasive structures.

(G) Left, cells stained for β-galactosidase (blue). Right, quantification of β-galactosidase positive cells. Scale bar: 40 μM.

(H) Left, quantification of γH2AX foci. Right, cells were stained for DNA (green) and γH2AX (magenta). L, large nuclei. Number of cells MCF10A.PLK4 −DOX = 469; −DOX+H2O2 = 518; +DOX = 466; +DOX+H2O2 = 377. Scale bar: 20 μM.

(I) Quantification of invasive structures.

(J) Ratio of GSH/GSSG.

(K) IL-8 secretion in cells treated with apocynin.

(L) Quantification of invasive structures. For all graphics error bars, represent mean ± SD from three independent experiments. *$p < 0.05$, **$p < 0.01$, ***$p < 0.001$, ****$p < 0.0001$; ns not significant.

See also Figure S6 and Table S1.

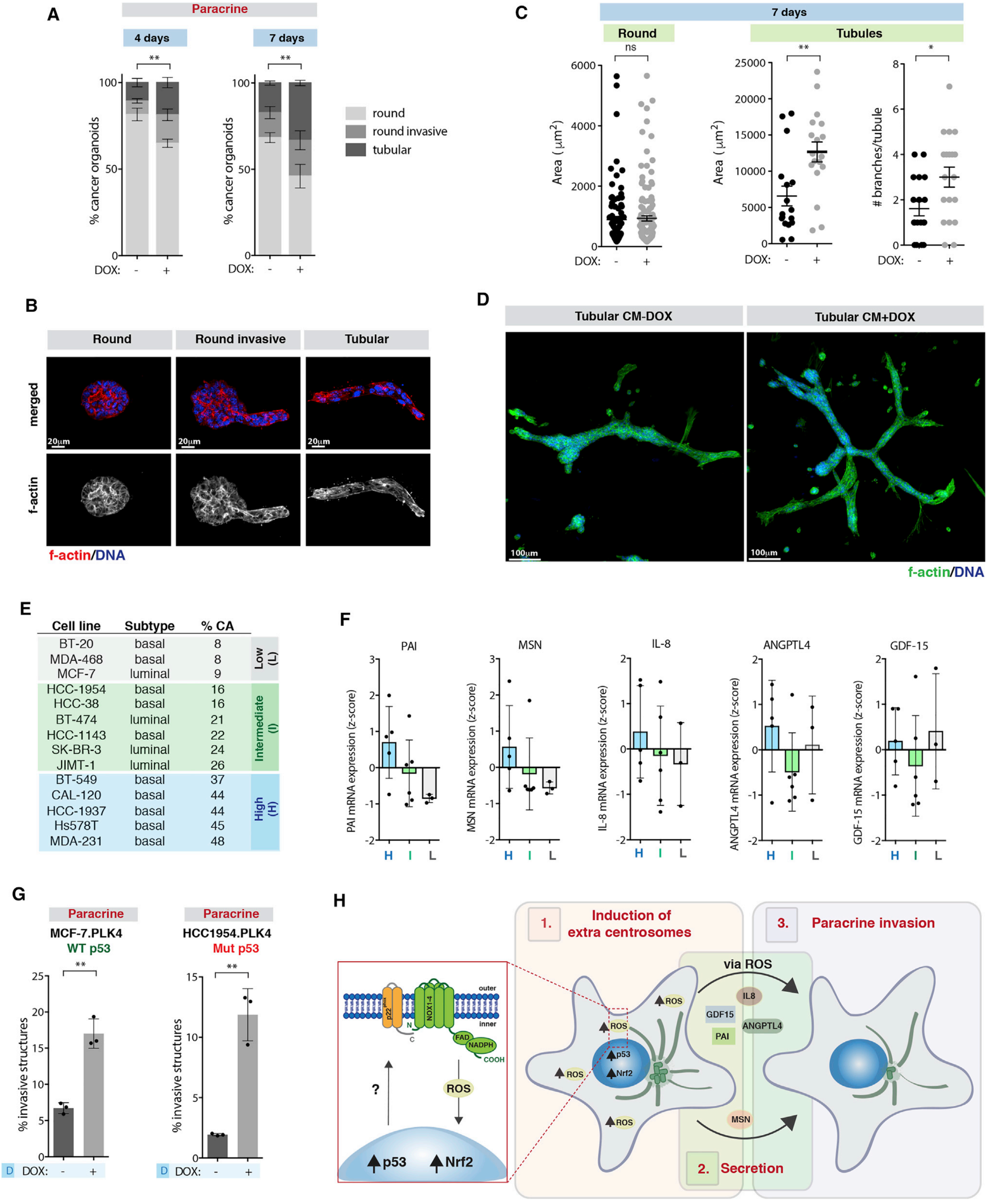

Figure 7. Centrosome Amplification in Breast Cancer Mediates Paracrine Invasion and Is Associated with IL-8 Secretion

(A) Quantification of round invasive and tubular structures in PyMT-derived tumor organoids.

(B) Tumor organoids. Scale bar: 20 μM.

(legend continued on next page)

MSN, and GDF-15, previously implicated in cancer invasion (Chang et al., 2012; Duffy, 2004; Tan et al., 2012; Wang et al., 2017; Waugh and Wilson, 2008). The combination of some of these factors (IL-8, ANGPTL4, and GDF-15) was sufficient to induce paracrine invasion, suggesting that multiple pathways are involved. One of the pathways important for this process is HER2 signaling, which can be activated by Src kinase downstream of the IL-8-CXCR1/2 axis (Singh et al., 2013). It is still unclear which pathways downstream of GDF-15 and ANGPTL4 are involved. In cancer, the cognitive receptor for GDF-15 is unknown; however, the C-terminal fragment of ANGPTL4, used in this study, can bind and activate β1 and β5 integrins, which could aid invasion (Goh et al., 2010). In breast cancer cells, centrosome amplification drives paracrine invasion and is correlated with the expression of pro-invasive factors, highlighting the importance of these findings in cancer.

IL-8, a recognized pro-inflammatory chemokine, is overexpressed in tumors and plays roles in invasion, proliferation, and survival of tumor cells, as well as in angiogenesis and immune infiltration (Liu et al., 2016; Waugh and Wilson, 2008). Consequently, elevated serum levels of IL-8 are associated with distant metastasis and considered an unfavorable prognostic factor in breast cancer (Benoy et al., 2004; Milovanovic et al., 2013). How IL-8 expression and secretion is regulated in tumors is not completely understood. IL-8 regulation occurs mostly at the transcriptional level, and its expression is induced by inflammatory signals (e.g., tumor necrosis factor α, IL-1β), environmental stresses (e.g., hypoxia) and exposure to chemotherapy agents (e.g., 5-fluorouracil, paclitaxel) (Waugh and Wilson, 2008). We found that centrosome amplification can lead to increased IL-8 expression. Moreover, our data suggest that early IL-8 secretion is induced by NOX-mediated ROS production. These findings are reminiscent of what has been observed in neutrophils in which rapid release of IL-8 was shown to be a consequence of NOX-induced ROS (Hidalgo et al., 2015). Our work suggests that the presence of extra centrosomes could regulate IL-8 production and/or secretion in tumors. Supporting this idea, we found that IL-8 expression correlates with centrosome amplification in breast cancer cell lines. Moreover, breast cancer stem cells with high levels of the ubiquitin-specific protease 44 (USP44) display centrosome amplification and increased IL-8 expression, which promotes vascularization and predicts aggressive behavior (Liu et al., 2015).

Generation of ROS is vital to redox signaling. To prevent oxidative stress, ROS levels are exquisitely balanced by pro- and antioxidants (Terada, 2006). This balance is often perturbed in cancer, leading to overall higher ROS levels. However, because high levels of ROS are toxic, tumors develop strong antioxidant mechanisms to prevent cell death (Harris et al., 2015). NRF2, one of the major regulators of antioxidant responses, is often stabilized in response to oncogenes such as K-RAS and MYC and is essential for tumor detoxification and growth (DeNicola et al., 2011; Sporn and Liby, 2012). We demonstrate that NFR2 is also stabilized and accumulates in the nucleus where it drives an antioxidant transcriptional response downstream of centrosome amplification. This could be important to prevent ROS-induced damage and a vital adaptation mechanism to centrosome amplification. ROS increase in cells with amplified centrosomes appears to be mediated by p53 stabilization, since p53 depletion decreases ROS-mediated IL-8 secretion and prevents paracrine invasion. This is consistent with the central role of p53 in ROS production (Liu et al., 2008). p53 overexpression was shown to increase ROS levels via transactivation of several ROS-generating enzymes such as NQO1 as well as $p67^{phox}$, an activating subunit of NOX2 complex, while suppressing the expression of antioxidant genes (Drane et al., 2001; Italiano et al., 2012; Polyak et al., 1997; Vousden and Lane, 2007).

At higher levels, ROS can mediate senescence and apoptosis downstream of p53 (Vigneron and Vousden, 2010; Wiley and Campisi, 2016). Indeed, centrosome amplification can induce senescence in a fraction of RPE-1 and BF cells. However, in MCF10A cells, centrosome amplification only induced a senescence-like response. This is consistent with our findings demonstrating that MCF10A cells do not develop a strong SASP. This further supports that the early secretory response we observe in these cells precedes senescence. The differences in response to centrosome amplification fit with the idea that the type and extent of the p53-inducing stress and/or cellular context determines the outcome of ROS production (Kastenhuber and Lowe, 2017). Thus, it is plausible that the response to ROS downstream of extra centrosomes varies among cell types, and it will be interesting to investigate if this would culminate with different secretory signatures. It also suggests that only cells with robust antioxidant mechanisms are able to resist centrosome amplification-induced senescence. Intriguingly, both in tumors and liver, where strong antioxidant mechanisms are important for cell survival, accumulation of cells with extra centrosomes can be observed without induction of a senescence phenotype (Duncan, 2013; Gorrini et al., 2013), (Schwabe and Brenner, 2006). We propose that in order for these cells to efficiently proliferate, they require effective mechanisms to prevent ROS-induced senescence. Since cellular senescence can act as a safeguard against tumorigenesis, it is possible that escaping from ROS-induced senescence could allow cells with extra centrosomes to become tumorigenic while affecting the surrounding cells via paracrine signaling.

Recent work showed that supernumerary centrosomes are sufficient to drive tumorigenesis in mice (Levine et al., 2017). Although these tumors show recurrent aneuploidies, the role of aneuploidy in the initiation of extra centrosome-derived tumors

(C) Area and branching of the tumor organoids. Error bars represent mean ± SEM.
(D) PyMT tubular organoids. Scale bar: 100 μM.
(E) Levels of centrosome amplification and breast cancer subtype.
(F) mRNA expression levels of pro-invasive factors.
(G) Quantification of invasive structures.
(H) Schematic representation of how centrosome amplification promotes secretion and paracrine invasion.
Unless specified, for all graphics, error bars represent mean ± SD from three independent experiments. *$p < 0.05$, **$p < 0.01$; ns, not significant.
See also Figure S7 and Table S1.

remains elusive. Our work, demonstrating that centrosome amplification induces ROS production, suggests that ROS signaling and DNA mutagenesis could play a role in tumor initiation. These findings also highlight ROS as a potential weakness of cells with extra centrosomes, and perhaps therapies that specifically target antioxidant pathways, currently under clinical trials, could be critical in targeting these cells (Gorrini et al., 2013). Indeed, we found that low doses of H2O2 decrease the proliferation of cells with extra centrosomes. The demonstration that centrosome amplification alters protein secretion indicates that cells carrying extra centrosomes have the ability to change the surrounding tumor cells as well as the tumor microenvironment. Hence, as the therapeutic potential of targeting subset of cells with extra centrosomes within a tumor remains uncertain (Godinho and Pellman, 2014), our findings raise the exciting possibility that targeting these cells could have a bigger impact in the clinic than anticipated. The notion that cells with extra centrosomes could be a source of pro-tumorigenic factors, such as IL-8, indicates that tumors could benefit from having cells with extra centrosomes. We postulate that these non-cell-autonomous advantageous effects could help to explain why cells with amplified centrosomes, despite their fitness cost, are kept during tumor evolution.

STAR★METHODS

Detailed methods are provided in the online version of this paper and include the following:

- KEY RESOURCES TABLE
- CONTACT FOR REAGENT AND RESOURCE SHARING
- EXPERIMENTAL MODEL AND SUBJECT DETAILS
  - Cell Culture
  - Mouse Mammary Organoids
  - Zebrafish Embryo Xenograft Model
  - Mouse Tumor Organoids
- METHOD DETAILS
  - Lentiviral Production and Infection
  - Conditioned Media
  - Chemicals
  - Recombinant Proteins
  - Indirect Immunofluorescence 2D
  - Indirect Immunofluorescence 3D
  - Long-Term Live-Cell Imaging
  - ELISAS
  - Nuclear Fraction Isolation
  - Western Blotting
  - siRNA
  - qRT-PCR
  - Secretomics Optimization
  - Mass Spectrometry
  - Analyses of the Extracellular Protein Compartment
  - β-Galactosidase Staining
  - Measure of ROS Production
  - Human Phospho-Receptor Tyrosine Kinase Array
  - Human XL Oncology Array Kit
  - Human SASP Array Kit
  - Microarray Analysis and GSEA
  - CCLE Expression Analysis
  - Exosomes and Microvesicle Isolation
- DATA AND SOFTWARE AVAILABILITY
- QUANTIFICATION AND STATISTICAL ANALYSIS
  - Statistics

SUPPLEMENTAL INFORMATION

Supplemental Information includes seven figures, seven tables, and four videos and can be found with this article online at https://doi.org/10.1016/j.devcel.2018.10.026.

ACKNOWLEDGMENTS

We are grateful to members of the Godinho lab, S. McClelland, D. Calado, and T. Sharp for comments or discussion of the manuscript; C. Brennan for helping with the zebrafish embryo injections; and P. Schmidt, A. O'Loghlen, and S. Kermorgant for reagents. This work was supported by a Cancer Research UK Centre Grant to Barts Cancer Institute (C16420/A18066). P.R.C. was funded by BBSRC (BB/M006174/1) and Barts and The London Charity (297/2249). S.A.G. is a fellow of the Lister Institute and is supported by the Medical Research Council (MR/M010414/1).

AUTHOR CONTRIBUTIONS

Conceptualization, T.A. and S.A.G.; Methodology, T.A., V.L.B., I.M., and S.A.G.; Investigation, T.A., P.M., S.D.A., V.R., P.R.C., and S.A.G.; Formal Analysis, E.G., J.M., and C.C.; Resources, V.L.B. and I.M.; Writing – Original Draft, S.A.G.; Writing – Review & Editing, T.A., P.M., S.D.A., and S.A.G.; Supervision, S.A.G.; Funding Acquisition, P.R.C. and S.A.G.

DECLARATION OF INTERESTS

The authors declare no competing interests.

Received: September 3, 2018
Revised: October 10, 2018
Accepted: October 23, 2018
Published: November 19, 2018

REFERENCES

Acosta, J.C., Banito, A., Wuestefeld, T., Georgilis, A., Janich, P., Morton, J.P., Athineos, D., Kang, T.W., Lasitschka, F., Andrulis, M., et al. (2013). A complex secretory program orchestrated by the inflammasome controls paracrine senescence. Nat. Cell Biol. *15*, 978–990.

Arnandis, T., and Godinho, S.A. (2015). Studying centrosome function using three-dimensional cell cultures. Methods Cell Biol. *129*, 37–50.

Barretina, J., Caponigro, G., Stransky, N., Venkatesan, K., Margolin, A.A., Kim, S., Wilson, C.J., Lehar, J., Kryukov, G.V., Sonkin, D., et al. (2012). The Cancer Cell Line Encyclopedia enables predictive modelling of anticancer drug sensitivity. Nature *483*, 603–607.

Basto, R., Brunk, K., Vinadogrova, T., Peel, N., Franz, A., Khodjakov, A., and Raff, J.W. (2008). Centrosome amplification can initiate tumorigenesis in flies. Cell *133*, 1032–1042.

Benoy, I.H., Salgado, R., Van Dam, P., Geboers, K., Van Marck, E., Scharpe, S., Vermeulen, P.B., and Dirix, L.Y. (2004). Increased serum interleukin-8 in patients with early and metastatic breast cancer correlates with early dissemination and survival. Clin. Cancer Res. *10*, 7157–7162.

Bettencourt-Dias, M., and Glover, D.M. (2007). Centrosome biogenesis and function: centrosomics brings new understanding. Nat. Rev. Mol. Cell Biol. *8*, 451–463.

Block, K., and Gorin, Y. (2012). Aiding and abetting roles of NOX oxidases in cellular transformation. Nat. Rev. Cancer *12*, 627–637.

Bolesta, E., Pfannenstiel, L.W., Demelash, A., Lesniewski, M.L., Tobin, M., Schlanger, S.E., Nallar, S.C., Papadimitriou, J.C., Kalvakolanu, D.V., and Gastman, B.R. (2012). Inhibition of Mcl-1 promotes senescence in cancer cells: implications for preventing tumor growth and chemotherapy resistance. Mol. Cell. Biol. *32*, 1879–1892.

Brenner, A.J., and Aldaz, C.M. (1995). Chromosome 9p allelic loss and p16/CDKN2 in breast cancer and evidence of p16 inactivation in immortal breast epithelial cells. Cancer Res. *55*, 2892–2895.

Cacalano, G., Lee, J., Kikly, K., Ryan, A.M., Pitts-Meek, S., Hultgren, B., Wood, W.I., and Moore, M.W. (1994). Neutrophil and B cell expansion in mice that lack the murine IL-8 receptor homolog. Science *265*, 682–684.

Casado, P., Rodriguez-Prados, J.C., Cosulich, S.C., Guichard, S., Vanhaesebroeck, B., Joel, S., and Cutillas, P.R. (2013). Kinase-substrate enrichment analysis provides insights into the heterogeneity of signaling pathway activation in leukemia cells. Sci. Signal. *6*, rs6.

Casilli, F., Bianchini, A., Gloaguen, I., Biordi, L., Alesse, E., Festuccia, C., Cavalieri, B., Strippoli, R., Cervellera, M.N., Di Bitondo, R., et al. (2005). Inhibition of interleukin-8 (CXCL8/IL-8) responses by repertaxin, a new inhibitor of the chemokine receptors CXCR1 and CXCR2. Biochem. Pharmacol. *69*, 385–394.

Chan, J.Y. (2011). A clinical overview of centrosome amplification in human cancers. Int. J. Biol. Sci. *7*, 1122–1144.

Chang, M.C., Chen, C.A., Chen, P.J., Chiang, Y.C., Chen, Y.L., Mao, T.L., Lin, H.W., Lin Chiang, W.H., and Cheng, W.F. (2012). Mesothelin enhances invasion of ovarian cancer by inducing MMP-7 through MAPK/ERK and JNK pathways. Biochem. J. *442*, 293–302.

Chao, J., Taveras, A.G., Chao, J., Aki, C., Dwyer, M., Yu, Y., Purakkattle, B., Rindgen, D., Jakway, J., Hipkin, W., et al. (2007). C(4)-alkyl substituted furanyl cyclobutenediones as potent, orally bioavailable CXCR2 and CXCR1 receptor antagonists. Bioorg. Med. Chem. Lett. *17*, 3778–3783.

Chen, Y., Zhang, Y., Yin, Y., Gao, G., Li, S., Jiang, Y., Gu, X., and Luo, J. (2005). SPD–a web-based secreted protein database. Nucleic Acids Res. *33*, D169–D173.

Cheung, K.J., Gabrielson, E., Werb, Z., and Ewald, A.J. (2013). Collective invasion in breast cancer requires a conserved basal epithelial program. Cell *155*, 1639–1651.

Coelho, P.A., Bury, L., Shahbazi, M.N., Liakath-Ali, K., Tate, P.H., Wormald, S., Hindley, C.J., Huch, M., Archer, J., Skarnes, W.C., et al. (2015). Over-expression of Plk4 induces centrosome amplification, loss of primary cilia and associated tissue hyperplasia in the mouse. Open Biol. *5*, 150209.

Coppe, J.P., Patil, C.K., Rodier, F., Sun, Y., Munoz, D.P., Goldstein, J., Nelson, P.S., Desprez, P.Y., and Campisi, J. (2008). Senescence-associated secretory phenotypes reveal cell-nonautonomous functions of oncogenic RAS and the p53 tumor suppressor. PLoS Biol. *6*, 2853–2868.

Costa-Silva, B., Aiello, N.M., Ocean, A.J., Singh, S., Zhang, H., Thakur, B.K., Becker, A., Hoshino, A., Mark, M.T., Molina, H., et al. (2015). Pancreatic cancer exosomes initiate pre-metastatic niche formation in the liver. Nat. Cell Biol. *17*, 816–826.

Davalos, A.R., Kawahara, M., Malhotra, G.K., Schaum, N., Huang, J., Ved, U., Beausejour, C.M., Coppe, J.P., Rodier, F., and Campisi, J. (2013). p53-dependent release of Alarmin HMGB1 is a central mediator of senescent phenotypes. J. Cell Biol. *201*, 613–629.

DeNicola, G.M., Karreth, F.A., Humpton, T.J., Gopinathan, A., Wei, C., Frese, K., Mangal, D., Yu, K.H., Yeo, C.J., Calhoun, E.S., et al. (2011). Oncogene-induced Nrf2 transcription promotes ROS detoxification and tumorigenesis. Nature *475*, 106–109.

Drane, P., Bravard, A., Bouvard, V., and May, E. (2001). Reciprocal down-regulation of p53 and SOD2 gene expression-implication in p53 mediated apoptosis. Oncogene *20*, 430–439.

Duffy, M.J. (2004). The urokinase plasminogen activator system: role in malignancy. Curr. Pharm. Des. *10*, 39–49.

Duncan, A.W. (2013). Aneuploidy, polyploidy and ploidy reversal in the liver. Semin. Cell Dev. Biol. *24*, 347–356.

Fava, L.L., Schuler, F., Sladky, V., Haschka, M.D., Soratroi, C., Eiterer, L., Demetz, E., Weiss, G., Geley, S., Nigg, E.A., et al. (2017). The PIDDosome activates p53 in response to supernumerary centrosomes. Genes Dev. *31*, 34–45.

Fialkow, L., Wang, Y., and Downey, G.P. (2007). Reactive oxygen and nitrogen species as signaling molecules regulating neutrophil function. Free Radic. Biol. Med. *42*, 153–164.

Ganem, N.J., Godinho, S.A., and Pellman, D. (2009). A mechanism linking extra centrosomes to chromosomal instability. Nature *460*, 278–282.

Ganier, O., Schnerch, D., Oertle, P., Lim, R.Y., Plodinec, M., and Nigg, E.A. (2018). Structural centrosome aberrations promote non-cell-autonomous invasiveness. EMBO J *37*, https://doi.org/10.15252/embj.201798576.

Godinho, S.A., and Pellman, D. (2014). Causes and consequences of centrosome abnormalities in cancer. Philos. Trans. R. Soc. Lond. B Biol. Sci. *369*, 20130467.

Godinho, S.A., Picone, R., Burute, M., Dagher, R., Su, Y., Leung, C.T., Polyak, K., Brugge, J.S., Thery, M., and Pellman, D. (2014). Oncogene-like induction of cellular invasion from centrosome amplification. Nature *510*, 167–171.

Goh, Y.Y., Pal, M., Chong, H.C., Zhu, P., Tan, M.J., Punugu, L., Lam, C.R., Yau, Y.H., Tan, C.K., Huang, R.L., et al. (2010). Angiopoietin-like 4 interacts with integrins beta1 and beta5 to modulate keratinocyte migration. Am. J. Pathol. *177*, 2791–2803.

Gorrini, C., Harris, I.S., and Mak, T.W. (2013). Modulation of oxidative stress as an anticancer strategy. Nat. Rev. Drug Discov. *12*, 931–947.

Guderian, G., Westendorf, J., Uldschmid, A., and Nigg, E.A. (2010). Plk4 trans-autophosphorylation regulates centriole number by controlling betaTrCP-mediated degradation. J. Cell Sci. *123*, 2163–2169.

Harris, I.S., Treloar, A.E., Inoue, S., Sasaki, M., Gorrini, C., Lee, K.C., Yung, K.Y., Brenner, D., Knobbe-Thomsen, C.B., Cox, M.A., et al. (2015). Glutathione and thioredoxin antioxidant pathways synergize to drive cancer initiation and progression. Cancer Cell *27*, 211–222.

Hidalgo, M.A., Carretta, M.D., Teuber, S.E., Zarate, C., Carcamo, L., Concha, I.I., and Burgos, R.A. (2015). fMLP-induced IL-8 release is dependent on NADPH oxidase in human neutrophils. J. Immunol. Res. *2015*, 120348.

Holland, A.J., Fachinetti, D., Zhu, Q., Bauer, M., Verma, I.M., Nigg, E.A., and Cleveland, D.W. (2012). The autoregulated instability of Polo-like kinase 4 limits centrosome duplication to once per cell cycle. Genes Dev. *26*, 2684–2689.

Italiano, D., Lena, A.M., Melino, G., and Candi, E. (2012). Identification of NCF2/p67phox as a novel p53 target gene. Cell Cycle *11*, 4589–4596.

Kastenhuber, E.R., and Lowe, S.W. (2017). Putting p53 in context. Cell *170*, 1062–1078.

Krzywicka-Racka, A., and Sluder, G. (2011). Repeated cleavage failure does not establish centrosome amplification in untransformed human cells. J. Cell Biol. *194*, 199–207.

Kwon, M., Godinho, S.A., Chandhok, N.S., Ganem, N.J., Azioune, A., Thery, M., and Pellman, D. (2008). Mechanisms to suppress multipolar divisions in cancer cells with extra centrosomes. Genes Dev. *22*, 2189–2203.

Levine, M.S., Bakker, B., Boeckx, B., Moyett, J., Lu, J., Vitre, B., Spierings, D.C., Lansdorp, P.M., Cleveland, D.W., Lambrechts, D., et al. (2017). Centrosome amplification is sufficient to promote spontaneous tumorigenesis in mammals. Dev. Cell *322*, 313–322.e5.

Liu, B., Chen, Y., and St Clair, D.K. (2008). ROS and p53: a versatile partnership. Free Radic. Biol. Med. *44*, 1529–1535.

Liu, Q., Li, A., Tian, Y., Wu, J.D., Liu, Y., Li, T., Chen, Y., Han, X., and Wu, K. (2016). The CXCL8-CXCR1/2 pathways in cancer. Cytokine Growth Factor Rev. *31*, 61–71.

Liu, T., Sun, B., Zhao, X., Li, Y., Zhao, X., Liu, Y., Yao, Z., Gu, Q., Dong, X., Shao, B., et al. (2015). USP44+ cancer stem cell subclones contribute to breast cancer aggressiveness by promoting vasculogenic mimicry. Mol. Cancer Ther. *14*, 2121–2131.

Loncarek, J., Hergert, P., and Khodjakov, A. (2010). Centriole reduplication during prolonged interphase requires procentriole maturation governed by Plk1. Curr. Biol. *20*, 1277–1282.

Marteil, G., Guerrero, A., Vieira, A.F., de Almeida, B.P., Machado, P., Mendonca, S., Mesquita, M., Villarreal, B., Fonseca, I., Francia, M.E., et al. (2018). Over-elongation of centrioles in cancer promotes centriole amplification and chromosome missegregation. Nat. Commun. *9*, 1258.

Marusyk, A., Tabassum, D.P., Altrock, P.M., Almendro, V., Michor, F., and Polyak, K. (2014). Non-cell-autonomous driving of tumour growth supports sub-clonal heterogeneity. Nature *514*, 54–58.

McGranahan, N., and Swanton, C. (2017). Clonal heterogeneity and tumor evolution: past, present, and the future. Cell *168*, 613–628.

Milovanovic, J., Todorovic-Rakovic, N., and Abu Rabi, Z. (2013). The prognostic role of interleukin-8 (IL-8) and matrix metalloproteinases -2 and -9 in lymph node-negative untreated breast cancer patients. J. BUON *18*, 866–873.

Mittal, K., Choi, D.H., Ogden, A., Donthamsetty, S., Melton, B.D., Gupta, M.V., Pannu, V., Cantuaria, G., Varambally, S., Reid, M.D., et al. (2017). Amplified centrosomes and mitotic index display poor concordance between patient tumors and cultured cancer cells. Sci. Rep. *7*, 43984.

Murphy, M.P. (2009). How mitochondria produce reactive oxygen species. Biochem. J. *417*, 1–13.

Nguyen-Ngoc, K.V., Shamir, E.R., Huebner, R.J., Beck, J.N., Cheung, K.J., and Ewald, A.J. (2015). 3D culture assays of murine mammary branching morphogenesis and epithelial invasion. Methods Mol. Biol. *1189*, 135–162.

Nigg, E.A., and Stearns, T. (2011). The centrosome cycle: centriole biogenesis, duplication and inherent asymmetries. Nat. Cell Biol. *13*, 1154–1160.

Ogura, M., Bridgeman, V.L., and Malanchi, I. (2017). Macrophages unlock progression of breast cancer cells experiencing matrigel-segregation in transplantation models. Sci. Rep. *7*, 11028.

Perkins, D.N., Pappin, D.J., Creasy, D.M., and Cottrell, J.S. (1999). Probability-based protein identification by searching sequence databases using mass spectrometry data. Electrophoresis *20*, 3551–3567.

Polyak, K., Xia, Y., Zweier, J.L., Kinzler, K.W., and Vogelstein, B. (1997). A model for p53-induced apoptosis. Nature *389*, 300–305.

Rajeeve, V., Vendrell, I., Wilkes, E., Torbett, N., and Cutillas, P.R. (2014). Cross-species proteomics reveals specific modulation of signaling in cancer and stromal cells by phosphoinositide 3-kinase (PI3K) inhibitors. Mol. Cell. Proteomics *13*, 1457–1470.

Rhys, A.D., and Godinho, S.A. (2017). Dividing with extra centrosomes: a double edged sword for cancer cells. Adv. Exp. Med. Biol. *1002*, 47–67.

Rhys, A.D., Monteiro, P., Smith, C., Vaghela, M., Arnandis, T., Kato, T., Leitinger, B., Sahai, E., McAinsh, A., Charras, G., et al. (2018). Loss of E-cadherin provides tolerance to centrosome amplification in epithelial cancer cells. J. Cell Biol. *217*, 195–209.

Rodrigues-Martins, A., Bettencourt-Dias, M., Riparbelli, M., Ferreira, C., Ferreira, I., Callaini, G., and Glover, D.M. (2007). DSAS-6 organizes a tube-like centriole precursor, and its absence suggests modularity in centriole assembly. Curr. Biol. *17*, 1465–1472.

Santaguida, S., Richardson, A., Iyer, D.R., M'Saad, O., Zasadil, L., Knouse, K.A., Wong, Y.L., Rhind, N., Desai, A., and Amon, A. (2017). Chromosome mis-segregation generates cell-cycle-arrested cells with complex karyotypes that are eliminated by the immune system. Dev. Cell *651*, 638–651.e5.

Schwabe, R.F., and Brenner, D.A. (2006). Mechanisms of liver injury. I. TNF-alpha-induced liver injury: role of IKK, JNK, and ROS pathways. Am. J. Physiol. Gastrointest. Liver Physiol. *290*, G583–G589.

Sercin, O., Larsimont, J.C., Karambelas, A.E., Marthiens, V., Moers, V., Boeckx, B., Le Mercier, M., Lambrechts, D., Basto, R., and Blanpain, C. (2016). Transient PLK4 overexpression accelerates tumorigenesis in p53-deficient epidermis. Nat. Cell Biol. *18*, 100–110.

Singer, M., and Sansonetti, P.J. (2004). IL-8 is a key chemokine regulating neutrophil recruitment in a new mouse model of Shigella-induced colitis. J. Immunol. *173*, 4197–4206.

Singh, J.K., Farnie, G., Bundred, N.J., Simoes, B.M., Shergill, A., Landberg, G., Howell, S.J., and Clarke, R.B. (2013). Targeting CXCR1/2 significantly reduces breast cancer stem cell activity and increases the efficacy of inhibiting HER2 via HER2-dependent and -independent mechanisms. Clin. Cancer Res. *19*, 643–656.

Sporn, M.B., and Liby, K.T. (2012). NRF2 and cancer: the good, the bad and the importance of context. Nat. Rev. Cancer *12*, 564–571.

Sternlicht, M.D. (2006). Key stages in mammary gland development: the cues that regulate ductal branching morphogenesis. Breast Cancer Res. *8*, 201.

Strnad, P., Leidel, S., Vinogradova, T., Euteneuer, U., Khodjakov, A., and Gonczy, P. (2007). Regulated HsSAS-6 levels ensure formation of a single procentriole per centriole during the centrosome duplication cycle. Dev. Cell *13*, 203–213.

Subramanian, A., Tamayo, P., Mootha, V.K., Mukherjee, S., Ebert, B.L., Gillette, M.A., Paulovich, A., Pomeroy, S.L., Golub, T.R., Lander, E.S., et al. (2005). Gene set enrichment analysis: a knowledge-based approach for interpreting genome-wide expression profiles. Proc. Natl. Acad. Sci. USA *102*, 15545–15550.

Tabassum, D.P., and Polyak, K. (2015). Tumorigenesis: it takes a village. Nat. Rev. Cancer *15*, 473–483.

Tan, M.J., Teo, Z., Sng, M.K., Zhu, P., and Tan, N.S. (2012). Emerging roles of angiopoietin-like 4 in human cancer. Mol. Cancer Res. *10*, 677–688.

Terada, L.S. (2006). Specificity in reactive oxidant signaling: think globally, act locally. J. Cell Biol. *174*, 615–623.

Vigneron, A., and Vousden, K.H. (2010). p53, ROS and senescence in the control of aging. Aging (Albany NY) *2*, 471–474.

Vousden, K.H., and Lane, D.P. (2007). p53 in health and disease. Nat. Rev. Mol. Cell Biol. *8*, 275–283.

Wang, L., Liu, Y., Li, W., and Song, Z. (2017). Growth differentiation factor 15 promotes cell viability, invasion, migration, and angiogenesis in human liver carcinoma cell line HepG2. Clin. Res. Hepatol. Gastroenterol. *41*, 408–414.

Waugh, D.J., and Wilson, C. (2008). The interleukin-8 pathway in cancer. Clin. Cancer Res. *14*, 6735–6741.

Werner, S., Pimenta-Marques, A., and Bettencourt-Dias, M. (2017). Maintaining centrosomes and cilia. J. Cell Sci. *130*, 3789–3800.

Wiley, C.D., and Campisi, J. (2016). From ancient pathways to aging cells-connecting metabolism and cellular senescence. Cell Metab. *23*, 1013–1021.

Wilkes, E.H., Terfve, C., Gribben, J.G., Saez-Rodriguez, J., and Cutillas, P.R. (2015). Empirical inference of circuitry and plasticity in a kinase signaling network. Proc. Natl. Acad. Sci. USA *112*, 7719–7724.

Zyss, D., and Gergely, F. (2009). Centrosome function in cancer: guilty or innocent? Trends Cell Biol. *19*, 334–346.

STAR★METHODS

KEY RESOURCES TABLE

REAGENT or RESOURCE	SOURCE	IDENTIFIER
Antibodies		
Rabbit Alexa-conjugated A488	Molecular Probes	#A11008; RRID: AB_143165
Mouse Alexa-conjugated A568	Molecular Probes	#A11011; RRID: AB_143157
Mouse Ki67 Alexa-conjugated A488	BD Biosciences	#561165; RRID: AB_10611866
Mouse Laminin V Alexa-conjugated A488	Millipore	#MAB19562X; RRID: AB_570380
Mouse α-tubulin	Sigma-Aldrich	#T9026; RRID: AB_477593
Rabbit centrin2 N-17-R	Santa Cruz	#sc-27793-R; RRID: AB_2082359
Mouse γ-H2AX S139	Merck Millipore	#05-636; RRID: AB_309864
Mouse α-smooth actin	Sigma-Aldrich	#A2547; RRID: AB_476701
Mouse p53	Santa Cruz	#sc-126; RRID: AB_628082
Rabbit p21	Cell Signaling	#2947-S; RRID: AB_823586
Rabbit β-actin	Cell Signaling	#4970; RRID: AB_2223172
Rabbit Histone H3	Cell Signaling	#9715S; RRID: AB_331563
Rabbit IL-8	Abcam	#ab106350; RRID: AB_10890102
Rabbit NRF2	Abcam	#ab62352; RRID: AB_944418
Rabbit ERK1/2	Cell Signaling	#4696; RRID: AB_390780
Rabbit p-ERK1/2 Thr202/Tyr204	Cell Signaling	#9101S; RRID: AB_331646
Rabbit MCAK	Bethyl Lab	#A300-807A-M; RRID: AB_577221
Mouse N-cadherin	BD Bioscience	#610920; RRID: AB_2077527
Mouse E-cadherin	BD Bioscience	#610181; RRID: AB_397580
Mouse Vimentin	BD Bioscience	#550513; RRID: AB_393716
Rabbit p-EGFR Tyr1068	Cell Signaling	#3777S; RRID: AB_2096270
Rabbit p-HER2 Tyr1221/1222	Cell Signaling	#2243S; RRID: AB_490899
Rabbit p-c-Met Tyr1234/1235	Cell Signaling	#3077S; RRID: AB_2143884
Chemicals, Peptides, and Recombinant Proteins		
Doxycycline hyclate	Sigma-Aldrich	#D9891
RO-3306	Sigma-Aldrich	#SML0569
Trastuzumab	Genentech	N/A
Erlotinib	Santa Cruz	#sc-202154
PHA-66752	Sigma-Aldrich	#PZ0147
NSC23766	Millipore	#553502
Reparixin	Cayman Chemical	#21492
SCH563705	MedChem Express	#HY-10011
PD98058	Sigma-Aldrich	#P215
PP2	Sigma-Aldrich	#P0042
H2O2	Sigma-Aldrich	#H1009
N-acetyl cysteine	Sigma-Aldrich	#A9165
Mitotempo	Sigma-Aldrich	#SML0737
Nutlin-3	Sigma-Aldrich	#N6287
Doxorubicin	Sigma-Aldrich	#D1515
Apocynin	Santa Cruz	#sc-203321
Antimycin-A	Abcam	#ab141904
DMEM/F12	Sigma-Aldrich	#D8437
DMEM	Thermo Fisher Scientific	#41966-029
RPMI	Thermo Fisher Scientific	#21875-034

(*Continued on next page*)

Continued

REAGENT or RESOURCE	SOURCE	IDENTIFIER
EGF	Sigma-Aldrich	#E4127
Insulin	Invitrogen	#12585-014
Hydrocortisone	Sigma-Aldrich	#H4001
Cholera toxin	Sigma-Aldrich	#C8052
Penicillin/Streptomycin	Thermo Fisher Scientific	#15140-122
Horse serum	Sigma-Aldrich	#H1138
FBS		#10500-064
Tet-free FBS	Hyclone	#SH30070.03T
Blasticidin	Generon	#2805-10
Geneticin (G418)	Thermo Fisher Scientific	#10131027
Polybrene	Sigma-Aldrich	#H9268
Immobilized trypsin, TPCK treated agarose resin	Thermo Fisher Scientific	#20230
CellTracker Green CMFDA	Thermo Fisher Scientific	#C2925
CellTracker CM-Dil Dye Red	Thermo Fisher Scientific	#C7001
Tricaine	Sigma-Aldrich	#E10521
Formaldehyde 16%	Thermo Fisher Scientific	#28908
Formalin	Sigma-Aldrich	#HT5012
Phalloidin Alexa A568	Molecular Probes	#12380
Hoechst 33342	Molecular Probes	#H3570
Mitosox	Molecular Probes	#M36008
ProLong anti-fade mounting medium	Molecular Probes	#P36934
BSA	Sigma-Aldrich	#A9647
Lipofectamine 2000	Invitrogen	#11668027
Lipofectamine RNAi Max	Invitrogen	#13778075
Power SYBR Green PCR Master Mix	Applied Biosystems	#4367659
RIPA Buffer	Thermo Scientific	#89901
Complete Mini Protease Inhibitor Cocktail	Roche	#11836153001
Phosphatase inhibitor Cocktail	Cell Signaling	#5870
Bradford Protein Assay	Bio-Rad	#5000006
Insulin-Transferrin-Selenium-Ethanolamine (ITS)	Thermo Fisher Scientific	#51500-056
Liberase Research Grade	Sigma-Aldrich	5401020001
Human IL-8	R&D	#208-IL-010
Human C-terminal ANGPTL4	R&D	#4487-AN-050
Human GDF-15	Invitrogen	#EHGDF-15
Human FGF	Sigma-Aldrich	#F0291
**Critical Commercial Assays**		
ELISA GDF-15 kit	Thermo Fisher Scientific	#EHGDF15
ELISA IL-8 kit	Abcam	#ab46032
ELISA PAI kit	Abcam	#ab108891
ELISA Mesothelin kit	R&D	#DMSLN0
ELISA Angiopoietin-like 4 kit	Thermo Fisher Scientific	#EHANGPTL4
ELISA HMGB1 kit	IBL	#ST51011
RNAeasy kit	Qiagen	#74104
High-capacity RNA-to-cDNA kit	Thermo Fisher Scientific	#4387406
Power SYBR Green	Thermo Fisher Scientific	#4367659
Pierce LDH Cytotoxicity Assay kit	Thermo Fisher Scientific	#88953
Senescent cells histochemical staining kit	Sigma-Aldrich	#CS0030-1KT
Cellular Reactive Oxygen Species detection kit	Abcam	#Ab113851
GSH/GSSH-Glo assay	Promega	#V6611

(*Continued on next page*)

Continued		
REAGENT or RESOURCE	SOURCE	IDENTIFIER
BCA Protein assay	Thermo Fisher Scientific	#23225
Human phosphor-receptor tyrosine kinase array kit	R&D	#ARY001B
Proteome Profiler Human XL Oncology array kit	R&D	#ARY026
Custom Human antibody array kit	RayBiotech	#AAH-CUST-M
Deposited Data		
Microarray Data	ArrayExpress	E-MTAB-6415
Experimental Models: Cell Lines		
MCF10A	ATCC	#CRL-10317
MCF10A.PLK4	Godinho et al. (2014)	N/A
HaCaT.PLK4	Godinho et al. (2014)	N/A
BF.PLK4	This work	N/A
RPE1.PLK4	Rhys et al. (2018)	N/A
MCF-7.PLK4	This work	N/A
HCC1954.PLK4	This work	N/A
BT-549	Prof. Peter Schmid (QMUL)	N/A
MDA-MB-231	Prof. Peter Schmid (QMUL)	N/A
MDA-MB-468	Prof. Peter Schmid (QMUL)	N/A
HEK293M	Prof. David Pellman (DFCI)	N/A
Experimental Models: Organisms/Strains		
Mouse: *Mus musculus* C57BL/57 strain	Charles River	JAX C57BL/6J
Mouse: *Mus musculus* B6.129S2(C) – $Cxcr2^{tm1Mwm}$/J	Jackson Laboratory	#006848
Zebrafish: *Danio rerio* $mitfa^{w2/w2}$; $mpv17^{a9/a9}$ (Casper)	N/A	N/A
Oligonucleotides		
siRNAs – See Table S6		N/A
qRT-PCR primers – See Table S7		N/A
Recombinant DNA		
pLenti-CMV-TetR-Blast	Addgene	#17492
pLenti-CMV/TO-Neo-DEST.PLK4	Godinho et al. (2014)	N/A
pInducer.PLK4	Rhys et al. (2018)	N/A
pMD2.G VSV-G	Addgene	#12259
psPAX2 Gag-Pol	Addgene	#12260
Other		
Vivaspin columns MWCO 5000 Da	GE Healthcare	#28-9323-59
8-well chamber slides	Corning	#354108
8-well chamber slides with glass bottom	ibidi	#80827

CONTACT FOR REAGENT AND RESOURCE SHARING

Further information and requests for resources and reagents should be directed to and will be fulfilled by the Lead Contact, Susana A. Godinho (s.godinho@qmul.ac.uk).

EXPERIMENTAL MODEL AND SUBJECT DETAILS

Cell Culture

Cell lines were maintained at 37°C with 5% CO_2 atmosphere. Human mammary epithelial MCF10A cells were grown in DMEM/F12 supplemented with 5% donor horse serum, 20 ng/ml epidermal growth factor (EGF), 10 mg/ml insulin, 100 mg/ml hydrocortisone, 1 ng/ml cholera toxin, 100 U/ml penicillin and streptomycin. HaCat (human keratinocytes; gift from J. Marshall-QMUL), BF (primary human fibroblasts; gift from A. O'Loghlen-QMUL), MDA-468 and MDA-231 (breast cancer; gift from P. Schmid-QMUL) were grown in DMEM supplemented with 10% FBS and 100 U/ml penicillin and streptomycin. RPE-1 (human retinal epithelial) were grown in DMEM/F12 supplemented with 10% FBS and 100 U/ml penicillin and streptomycin. MCF-7, HCC1954 and BT-549 (breast cancer; gift from P. Schmid-QMUL) were grown in RPMI supplemented with 10% FBS and 100 U/ml penicillin and streptomycin.

Tetracycline-free FBS was used to grow cells expressing the *PLK4* Tet-inducible construct, with the exception of MCF10A cells where horse serum was always used.

For 3D cultures, MCF10A cells were grown in the same medium with reduced horse serum (2%). To assay invasion in 3D cultures, cells were grown in a mix of matrigel: collagen-I, as previously described (Arnandis and Godinho, 2015). Growth factor-reduced matrigel with specific protein concentrations between 9 and 11 mg/ml was used. Note that due to the variability in the composition of the matrigel lots, we always tested the ability of cells with extra centrosomes to induce the formation of invasive protrusions (~20%) before we use it for our experiments.

Collagen-I was used at 1.6 mg/ml. Cells were grown for 4 days in 3D cultures before quantifying invasion. 150–200 acini were scored per condition for each experiment.

Mouse Mammary Organoids

Mammary gland organoids were prepared according to previously described methods (Nguyen-Ngoc et al., 2015). Briefly, mammary glands from C57BL/6J female mice between 8 and 12 weeks of age were isolated. In the sterile hood, mammary glands were minced with a scalpel, and the small pieces were transfer to a collagenase solution in DMEM/F12 (2 mg/mL collagenase, 2 mg/mL trypsin, 5 % v/v fetal bovine serum (FBS), 5 μg/mL insulin and 50 μg/mL gentamicin) on a shaker (150 rpm) for 35 min at 37°C. Tubes were spun in a centrifuge at 1500 rpm for 10 min at room temperature and the pellet was kept as the epithelial fraction containing the organoids. After further digestion of the pellet with DNase (2 U/μL), three more short-pulse washes at 1500 rpm were done, and organoid density was calculated by manual counting on the microscope. The structures were seeded at a 2 organoids/μl density and embedded in a mixture of Matrigel: Collagen (3:7) on eight well chambers. Organoid medium (DMEM/F12 with 1% penicillin/streptomycin, 1% ITS and 2.5 nM FGF2) was added on top and invasive organoids were quantified after 4 days. Mammary organoids were fixed with formaldehyde 4% and stained with α-SMA, Phalloidin and Hoechst 33342. Images were taken with a 710 Zeiss laser scanning confocal microscope. We quantified 100 organoids per conditions for each experiment.

C57BL/6J WT animals were obtained from Charles River: https://www.criver.com/products-services/find-model/jax-c57bl6j-mice?region=3616. CXCR2-/- BALB/c mice (*Cxcr2^{tm1Mwm}* knock-out) were obtained from Jackson Laboratories (Cacalano et al., 1994). WT littermates were used as controls. All animal experiments followed Home Office Guidelines determined by the Animals (Scientific Procedures) Act 1986.

Zebrafish Embryo Xenograft Model

Zebrafish (*Danio rerio*) were handled in compliance with local animal care regulations (Queen Mary University of London) under the Animals (Scientific Procedures) Act 1986 and standard protocols. Fish were kept at 28°C in aquaria with day/night light cycles (10 hr dark/14 hr light periods). The developing embryos were kept in an incubator at constant temperature.

MCF10A cells (1x10^6 cells) with normal or extra centrosomes (+DOX, 48 hrs) were stained in suspension with 10 mmol/L CellTracker Green CMFDA (Green) or 2.5 μg/ml CellTracker™ CM-DiI Dye (Red) during 30 min at 37°C. To remove unincorporated dye, cells were rinsed twice with PBS, and one third of the cells of each condition was mixed 1:1 for the co-injection experiments (~300-400 cells per embryo). 48 hr old zebrafish embryos were dechorionated and anesthetized with tricaine (Sigma-Aldrich) prior to implantation of the labelled cells in the perivitelline cavity with a manual injector (Picospritzer III, Parker Hannifin Instruments). After injections, embryos were incubated at 34°C. Three separate experiments were carried out per condition. Counting of disseminated cells was done 24 hrs after injections under high magnification using a Zeiss Axioplan epifluorescence microscope.

Mouse Tumor Organoids

MMTV-PyMT cell isolation and growth has been previously described (Ogura et al., 2017). Briefly, MMTV-PyMT tumors were isolated, mechanically minced and chemically digested using Liberase and DNase I in HBSS and passed through a 100 μm cell strainer. A single cell suspension of this PyMT primary tumor cells were seeded on a glass-bottom 8-well chamber at 2.5 x 10^3 cells/chamber in a 2:1 mixture of Collagen-I (Corning) and Matrigel (Corning) yielding a final collagen concentration of 4 mg/ml and a final Matrigel concentration of 2 mg/ml. Tumor organoids were grown in conditioned media supplemented with 10X concentrated MEM media (DMEM/F12 supplemented with 2% FCS, 10 μg/ml Insulin, 20 ng/ml EGF and 1:50 L-Glutamax at 37°C and 5% CO2 during 7 days. Tumor organoids were fixed with formaldehyde 4% and stained with Phalloidin and Hoechst 33342. Images were taken with a 710 Zeiss laser scanning confocal microscope. The degree of branching and percentage of tubular and rounded structures was manually quantified. Organoid growth and tubular expansion was obtained as the total Phalloidin area/structure using Image J Software. We quantified 100 organoids per condition for each experiment.

METHOD DETAILS

Lentiviral Production and Infection

Cells expressing the inducible PLK4 construct were previously described (Godinho et al., 2014). Briefly, the lentiviral vectors pLenti-CMV-TetR-Blast and p-Lenti-CMV/TO-Neo-Dest expressing the PLK4 cDNA were used consecutively. Cell lines were initially infected with a lentivirus containing the TetR and selected using Blasticidin (5-10 μg/ml). After selection cells were secondarily infected with the PLK4 containing lentivirus and selected with Geneticin (100-200 μg/ml). The selected cells were maintained as a pool to

make a cell population. To generate the HCC1954.PLK4 and BF.PLK4 cell lines we used the pInducer21 lentiviral vector in which the PLK4 cDNA was inserted using the Gateway system. In this case, positive cells were sorted according to GFP signal.

To generate lentivirus, HEK-293M cells were grown in antibiotic free medium and co-transfected with the lentiviral plasmid, VSV-G (pMD2.G) and Gag-Pol (psPAX2) using Lipofectamine 2000, according to the manufacturer's protocol. Lentivirus were harvested 24 and 48 hrs post infection and passed through a 0.45 μM syringe filter unit and stored at -80°C. To infect cells, 8 μg/ml polybrene was included to 1.5 ml of lentivirus and added on top of cells for 6 hrs. This process was repeated the following day and 48 hrs post initial infection. As specified above cells were treated with appropriate antibiotic for selection or amplified for cell sorting.

Conditioned Media

Cells were seeded in a 6 well plate (-DOX: $0.7x10^5$ cells/well; +DOX: $0.9x10^5$ cells/well) and incubated for 2 days in the presence or absence of DOX (2 μg/ml) until they reached 80%-85% confluency. After incubation, cells were washed three times with PBS to remove DOX and 900 μl phenol-free DMEM/F12 medium without serum was added on top of the cells during 16 hrs. After that, the conditioned medium (CM) was collected, centrifuged at 2,000g for 10 min and filtered through a 0.2 μm pore filter. CM was mixed with 10X concentrated 3D media (final concentration 1x) before being added on top of the 3D cultures. Cells were always counted to discard effects due to differences on cell number. If a particular treatment decreased the final cell number, cell seeding was adjusted so that the same cell numbers were obtained at the time of CM collection.

Trypsin treatment of the CM was done by adding 50 μl of beads with immobilized trypsin (TPCK Treated Agarose Resin) to 1 ml of conditioned media and incubated overnight with rotation at 37°C. The following day beads were removed by centrifugation and the CM was added on top of 3D cultures.

Vivaspin columns with a 5kDa cut-off membrane were used to separate larger fractions (>5kDa: proteins) from smaller fractions (<5kDA: metabolites, small molecules). 2 ml of conditioned media were added to the columns and centrifuged at 5000g for 30 min. When drugs were present in the CM, Vivaspin columns were washed away with phenol-free DMEM/F12 medium using 2 successive centrifugations before resuspending the remainder CM with DMEM/F12.

Chemicals

Doxycycline (DOX) was used at 2 mg/ml. The following doses of inhibitors were used: 5 μM R0-3306 (CDK1i), 40 μg/ml Trastuzumab (Herceptin, HER2 inhibitor), 0.5-4 μM Erlotinib (EGFR inhibitor), 1 μM PHA-66752 (c-met inhibitor), 25 μM NSC23766 (RAC1 inhibitor), 100 nM Reparixin (CXCR1/2 inhibitor), 100 nM SCH563705 (CXCR1/2 inhibitor), 20 μM PD98059 (ERK inhibitor), 5 μM PP2 (Src inhibitor), 100 μM H2O2 (Sigma), 5 mM NAC (Sigma), 0.5 mM Apocynin, 35 μM Antimycin-A, 10 μM Mitotempo, 5 μM Nutlin-3 and 100 ng/ml Doxorubicin (DoxoR).

Recombinant Proteins

Recombinant proteins were used at the following concentrations: IL-8 (0.5 μg/ml, 208-IL-010), c-terminal fragment of Angiopoietin-like 4 (4 μg/ml) and GDF-15 (0.01 μg/ml).

Indirect Immunofluorescence 2D

Cells plated in glass coverslips (2D) were washed in PBS and fixed with ice-cold methanol at -20ºC for 10 min for centrin2 staining. Following fixation cells were permeabilized in 0.2% Triton X-100 in PBS for 5 min and blocked in blocking buffer (PBS, 5% BSA, 0.1% Triton X-100) during 30 min. Cells were then stained in primary antibodies diluted in blocking buffer for 60 min. Cells were washed with PBS and incubated 60 min with species-specific fluorescent secondary antibodies (Alexa-conjugated). DNA was stained with Hoechst 33342 (1:5000) for 5 min in PBS. Antibodies used included: anti-α-tubulin DM1α (1:1000), anti-centrin-2 N-17-R (1:100). For all conditions used in this work, centrosome amplification was determined as the percentage of mitotic cells containing extra centrosomes (Table S1). For Ki67 staining (assess cell viability) or γH2AX (assess dsDNA breaks), cells were fixed with 4% of formaldehyde 15 min at room temperature and stained using anti-Ki 67 antibody (1:500) and anti-γH2AX (1:200) diluted in 0.25% BSA. Images were acquired using an inverted Nikon microscope coupled with a spinning disk confocal head (Andor) and analyzed with ImageJ (National Institute of Health, Bethesda, MD, USA). Proliferating cells were quantified as the percentage of Ki67 positive nuclei and dsDNA breaks were quantified as the number of γH2AX-positive foci per nucleus using the NIS-Elements software (Nikon).

To assess mitochondrial ROS, live cells were incubated with 5 μM Mitosox for 10 min at 37ºC. Images were acquired using an inverted Nikon microscope coupled with a spinning disk confocal head (Andor). Images were analyzed with NIS-Elements software (Nikon). 100 cells in mitosis were used to quantify centrosome amplification per condition for each experiment.

Indirect Immunofluorescence 3D

Immunostainings of 3D cultures were performed on partially embedded 3D acini and breast and tumor organoids, both plated on eight-well chambers, according to previous protocols (Godinho et al., 2014; Ogura et al., 2017). Briefly, the media was removed and the structures were washed with PBS and fixed in 5% of formalin in PBS for 20 min at 37°C. After fixation cells were washed 3 times, 10 min each, with PBS: 100 mM glycine and permeabilized with 0.5% Triton X-100 in PBS for 10 min. Cells were blocked with 10% goat serum in IF buffer (130mM NaCl, 7 mM Na2HPO4, 3.5 mM NaH2PO4, 7.7 mM NaN3, 0.1% BSA, 0.2% Triton X-100, 0.05% Tween-20) for 1 hr at room temperature, and primary antibodies were incubated in this solution over night at 4°C. Cells were rinsed 3 times, 20 min each, with IF buffer. When required, cells were incubated with secondary antibodies for 1 hr at room

temperature (Alexa-conjugated). Cells were washed twice with IF buffer and once with PBS followed by 10 min incubation with Hoechst 33342 (1:2500). 3D cultures were mounted in ProLong Antifade mounting medium. Antibodies used include anti Laminin-V AlexaFluor 488 conjugated (1:100) and α-smooth muscle actin (1:150). For f-actin staining 3D cultures were incubated with Phalloidin (1:100; AlexaFluor 568) for 60 min. Images were taken with a 710 Zeiss laser scanning confocal microscope.

Long-Term Live-Cell Imaging

MCF10A cells plated in ibidi chambered slides were used for 3-D imaging. Cells were imaged on a Nikon Eclipse Ti-E inverted microscope equipped with a ORCA-Flash 4.0 camera, a precision motorized stage, and Nikon Perfect Focus, all controlled by NIS-Elements Software (Nikon). Microscope was enclosed within temperature and CO2-controlled environments that maintained an atmosphere of 37° C and 3%-5% humidified CO2. Phase contrast images were captured at every 10 minutes for 20 hrs with either Plan Fluor 10X (0.3 NA) or Plan Apo VC 20X (0.75 NA) objectives. Captured images from each experiment were analyzed using NIS-Elements software. Videos were played at 50 ms per frame.

ELISAS

Levels of secreted proteins were assessed in the CM collected from cells with normal or extra centrosome numbers (+DOX) using commercially available ELISA kits, following manufacturer's instructions: GDF-15, IL-8, PAI, Mesothelin, Angiopoietin-like 4 and HMGB1. Briefly, CM, collected as described above (see CM section), and specific protein standards were loaded on the specific-antibody coated wells of the supplied microplate, which bind to the immobilized (capture) antibody. A sandwich is formed by the addition of the biotinylated antibody, binding to the chemokine on a different epitope from the capture antibody. A conjugated enzyme (Streptavidin-Peroxidase) was added into the assay. After incubation periods and wash steps specified by every supplier to remove unbound antibody from the plate, a substrate solution was added in order to obtain a measurable signal. The intensity of this signal was proportional to the concentration of the protein present in the CM. Assays were performed in triplicate, and absorbance at 450 nm was read on a plate reader.

Nuclear Fraction Isolation

70% confluent cells on a 6-well plate were washed twice and scraped with PBS. This fraction was centrifuged at 850 g for 10 min to collect the cells. Cells were then lysed by 15 min incubation in hypotonic buffer (20 mM HEPES [pH 7.6], 20% glycerol, 10 mM NaCl, 1.5 mM MgCl2, 0.2 mM EDTA, 0.1% Triton X-100, 25 mM NaF, 25 mM β-glycerophosphate, 1 mM phenylmethylsulfonyl fluoride, 1 mM sodium orthovanadate, 1 mM dithiothreitol, and protease inhibitors) supplemented with detergents (NP-40, 10%). After this incubation, nuclei were collected by centrifugation (14000 g for 1 min at 4◦C) and supernatant recovered as cytosolic fraction. This pellet, including mainly intact nuclei, was lysed in a rocking platform for 30 min with gentle agitation and nuclear soluble fractions were collected after centrifugation (14000 g for 10 min at 4◦C).

Western Blotting

Cells were collected and resuspended in RIPA buffer supplemented with protease and phosphatase inhibitors. Protein concentration was quantified using the Bradford Protein Assay (20 μg was loaded per well). Protein samples were then resuspended in Laemmli buffer and separated on sodium dodecyl sulphate polyacrylamide gel electrophoresis (SDS-PAGE) and transferred onto PVDF membranes. Antibodies used included anti-p53 (1:1000), anti-p21 (1:1000), anti-β-actin (1:5000), anti-α-tubulin (1:2000), anti-Histone H3 (1:10000), anti-Interleukin-8 (1:1000), anti-NRF2 (1:1000), anti-ERK (1:1000), anti-pERK Thr202/Tyr204 (1:1000), pEGFR Tyr1068 (1:1000) anti-MCAK (1:1000), anti-N-cadherin (1:500), anti-E-Cadherin (1:500), anti-Vimentin RV202 (1:500), anti-pHER2 Tyr1221/1222 (1:1000) and anti-p-c-Met Tyr1234/1235 (1:1000). Western blots were developed using SRX-101A Konica Minolta and scanned. The intensity of the bands was measured by densitometry using ImageJ (National Institute of Health, Bethesda, MD, USA).

To assess the levels of p-Erk1/2, cells pre-treated with HER2 (Trastuzumab, 40 μg/ml, 1 hr) and CXCR1/2 (SCH563705, 100 nM, 1 hr) inhibitors and incubated with CM for 10 min.

siRNA

siRNA was performed using Lipofectamine RNAiMax. 50 nM of siRNA was used per well in a 6-well plate. Cells were incubated with the transfection mix for 6 hrs, washed and normal growth medium was added. Cells were analyzed 48 hrs post transfection. siRNAs used are described in Table S5. To assess invasion in the siRNA mini screen, 150-200 acini were quantified per conditions in each experiment.

qRT-PCR

RNA was prepared using the Qiagen RNAeasy kit according to the manufacturer's instructions. 200 ng of RNA was used to produce cDNA using the High-Capacity RNA-to-cDNA kit according to the manufacturer's instructions. For qRT-PCR we used Power SYBR Green followed by analysis with 7500 Real Time PCR system (Applied Biosystems). All primers used for qRT-PCR are described in Table S6.

Secretomics Optimization

Secretome analysis was done in the CM collected from cells with normal or extra centrosomes (+DOX, 48 hrs). Since secreted proteins are often masked by high amounts of protein supplements in the culture medium, we used a modified serum-deprived method previously described (Acosta et al., 2013). After three washes with PBS, DMEM/F12phenol-free medium without serum was added on top of the cells during 16 hrs. After this incubation, CM was collected and cells were counted. The collected CM was assessed for protein concentration, measured using Bradford Protein Assay. Lactate dehydrogenase (LDH) detection was also assessed using a the LDH Cytotoxicity Assay Kit, as a measure of cell death. Samples were concentrated using Vivaspin Columns (Vivaspin MWCO 5000 Da) before proceeding to the protein analysis by mass spectrometry.

Mass Spectrometry

Proteomics experiments were performed using mass spectrometry as reported before (Casado et al., 2013; Rajeeve et al., 2014). Briefly, enriched CM proteins were digested with trypsin and resultant peptides were desalted using C18 plus carbon top tips (Glygen corparation, TT2MC18.96) and eluted with 70% acetonitrile (ACN) with 0.1% formic acid. Dried peptides were dissolved in 0.1% TFA and analyzed by nanoflow LC-MS/MS in an ultimate 3000 RSL nano instrument coupled on-line to a Q Exactive plus mass spectrometer (Thermo Fisher Scientific). A PepMap RP 75 μm ID x 150 mm column was used for peptide separation. Gradient elution was from 3% to 35% buffer B in 120 min at a flow rate 250nL/min with buffer A being used to balance the mobile phase (buffer A was 0.1% formic acid in water and B was 0.1% formic acid in ACN). The mass spectrometer was controlled by Xcalibur software (version 4.0) and operated in the positive ion mode. The spray voltage was 1.95 kV and the capillary temperature was set to 255°C. The Q-Exactive plus was operated in data dependent mode with one survey MS scan followed by 15 MS/MS scans. The full scans were acquired in the mass analyzer at 375- 1500m/z with the resolution of 70 000, and the MS/MS scans were obtained with a resolution of 17 500. MS raw files were converted into Mascot Generic Format using Mascot Distiller (version 2.5.1) and searched against the SwissProt database (release December 2015) restricted to human entries using the Mascot search daemon (version 2.5.0) (Perkins et al., 1999). Allowed mass windows were 10 ppm and 25 mmu for parent and fragment mass to charge values, respectively. Variable modifications included in searches were oxidation of methionine and pyro-glu (N-term). Label-free quantification was performed by calculating the peak areas of extracted ion chromatograms (XICs) for the respective peptide ion. Mass and time windows were 7ppm and 1.5 minutes respectively. Pescal was used to automate the generation of XICs as described (Wilkes et al., 2015). To reliably differentiate the extracellular components from intracellular contaminants, a filtering step was applied using different Databases: Gene ontology, Secreted Protein Database and Signal Peptide Database. From the Secreted Protein Database ranks 0 to 2 were considered as belonging to the extracellular compartment.

Analyses of the Extracellular Protein Compartment

To define the proteins from the mass spectrometry data that belonged to the extracellular compartment we used several databases, including Gene ontology, Secreted Protein Database and Signal Peptide Database. Secreted protein Database (SPD) consists of a core dataset and a reference dataset (Chen et al., 2005). The core dataset contains 18 152 secreted proteins retrieved from Swiss-Prot/TrEMBL, Ensembl, RefSeq and CBI-Gene. We used a combined automatic and manual processing to collect as much secreted proteins as possible. The dataset Rank0 from Swiss-Prot includes some partial sequences without the N- or C-termini. Given that most of the signal peptides are located at the N-terminal of proteins, we eliminated the entries without N-terminal methionine (Met, M) in CBI-Gene, Ensembl, Swiss-Prot/TrEMBL and RefSeq in our prediction results. Proteins in the datasets of Rank1, 2, 3 all have N-terminal Met. For our analyses we selected proteins that ranked 0-2 as belonging to the extracellular compartment since proteins in rank 3 have lower probability of belonging to the extracellular compartment (Chen et al., 2005).

β-Galactosidase Staining

Positive senescent cells were scored using a commercial available kit (Senescence Cells Histochemical Staining Kit). Briefly, cells in 6-well plates were washed, fixed and stained overnight with a staining mixture at 37°C without CO_2. Under these conditions, β-galactosidase activity is easily detectable in senescent cells, but undetectable in quiescent, immortal, or tumor cells. Percentage of positive cells was counted manually with a microscope. We quantified 1500-2000 cells per conditions for each experiment. For the H2O2 treatments, cells were incubated only 48 hrs with H2O2 after which cells were washed and left from the appropriate time (6-10 days) before assessing senescence. As control positive, cells were treated with 100 ng/ml DoxoR for the entire duration of the experiment.

Measure of ROS Production

ROS was measured using DCFDA (Cellular Reactive Oxygen Species Detection Assay Kit), a cell permeable fluorogenic dye that measures hydroxyl, peroxyl and other reactive oxygen species (ROS) activity within the cell. Briefly, cells were plated after 48 hrs of DOX treatment at a low confluency overnight ($20x10^4$ cells/ well) in 96 well transparent bottom black-plate. The following day, the media was removed and 20 μM of DCFDA was added to the corresponding wells and incubated for 30 min at 37°C. The dye was washed away before reading. Positive controls were treated with 25 μM DoxoR for 3 hrs. The signal was detected by fluorescence spectroscopy with maximum excitation and emission spectra of 495 nm and 529 nm respectively.

ROS production was also assessed through the detection of oxidized proteins. Here we measure the total amount of oxidized (GSSG) and reduced (GSH) glutathione using bioluminescent signals, according to manufacturer's instructions (GSH/GSSG-Glo™

Assay). Briefly, cells were plated at a low confluency overnight ($12x10^4$ cells/ well) in 96 well transparent bottom white-plate. The following day, the media was removed and reduced glutathione lysis reagent or oxidized glutathione lysis reagent were added to the corresponding wells and shake for 5 min at RT. Luciferin generation and detection reagent were added subsequently for 30 and 15 min, respectively. The bioluminescent signal was read per well using a plate reader luminometer. Final ratios were normalized to protein concentration, which was determined per well using BCA Protein Assay Kit. NAC (5 mM) and Apocynin (0.5 mM) were added at time of centrosome amplification induction (48 hrs) and DoxoR treatment was performed for 3 hrs (25 μM).

Human Phospho-Receptor Tyrosine Kinase Array

RTK activation was assessed using an antibody-based array (Human Phospho-Receptor Tyrosine Kinase Array Kit) following manufacturer's instructions. Briefly, cells were treated for 48hrs with CM collected from control cells and cells with extra centrosomes (+DOX, 48hrs) and supplemented with completed medium. After incubation, cells were lysed using a lysis buffer provided by the kit and protein concentration was determined using Bradford (Bio-Rad). 300 μg of protein cell lysates were added on top of the membranes and incubated overnight at 2-8°C on a rocking platform. Thereafter, a pan anti-phosphotyrosine antibody was added to detect the activated RTKs. After several washes of the membranes, phosphorylated RTKs were spotted using a Chemi Reagent Mix and developed in an autoradiography film cassette. Following quantification of scanned images using ImageJ software (National Institute of Health, Bethesda, MD, USA) by densitometry, the relative activation of specific phosphorylated RTKs between normal and cells with extra centrosomes was plotted.

Human XL Oncology Array Kit

We screened protein secretion using a membrane-based sandwich immunoassay (Proteome Profiler Human XL Oncology Array Kit), following manufacturer's protocol. Briefly, cell culture supernatants collected from normal or cells with extra centrosomes plated in 6-well plates were diluted and incubated overnight with the membrane arrays. These membranes contain a set of capture antibodies, spotted in duplicate, that bind to specific target proteins. The membranes were then washed to remove unbound material and incubated with a cocktail of biotinylated detection antibodies. Streptavidin-HRP and chemiluminescent detection reagents were then applied, and the signal was captured using autoradiography films. The intensity of every spot was measured by densitometry using ImageJ (National Institute of Health, Bethesda, MD, USA) and the relative intensity versus control was calculated and depicted.

Human SASP Array Kit

MCF10A.PLK4 and RPE-1.PLK4 cells were seeded in a 6 well plate [MCF10A.PLK4: $0.7x10^5$ cells for -DOX; $0.9x10^5$ for +DOX and $2x10^5$ for DoxoR (100ng/mL) and RPE-1.PLK4: $0.5x10^5$ cells for -DOX; $0.75x10^5$ for +DOX and $1.1x10^5$ for DoxoR (100ng/mL)] and incubated for 2 days in the presence or absence of DOX. For 48 hrs analyses, CM was collected as described above after DOX treatment. For 7 days analyses, cells were split at day 2 and day 4 and serum free medium was added at day 6 and collected at day 7. Senescence-Associated Secretory Phenotype (SASP) was screened using a membrane-based sandwich immunoassay (Custom C-series Human Antibody Array), following manufacturer's protocol. We defined a set of known SASP components, including: including IL-8, IL-6, uPar, MIP-3α, MCP-1, GRO -a, -b, -c and IL-1β, based on previous work (Coppe et al., 2008), that were spotted in duplicate on a membrane provided by RayBiotech. Cell culture supernatants collected from cells with normal or extra centrosomes and cells treated with DoxoR were diluted in serum-free media and blocking solution. Volume equivalent to 2.10^5 cells/condition was incubated overnight with the membrane arrays. The membranes were then washed to remove unbound material and incubated with a cocktail of biotinylated detection antibodies. Streptavidin-HRP and chemiluminescent detection reagents were then applied, and the signal was captured using autoradiography films. The intensity of every spot was measured by densitometry using ImageJ (National Institute of Health, Bethesda, MD, USA) and the relative intensity versus control was calculated and depicted.

Microarray Analysis and GSEA

Total RNA was extracted from MCF10A.PLK4 untreated (-DOX) or treated with DOX (+DOX) for 48 hrs using the RNeasy kit. RNA was hybridized against the Affymetrix HG-U133_Plus_2 microarrays according to manufacturer's instructions. Three biological replicates (3 -DOX and 3 +DOX) were analyzed with two technical replicates each. Genes differentially regulated between –DOX and +DOX groups were identified using limma with a false discovery rate (FDR) <0.05. Gene set enrichment analysis (GSEA) (Subramanian et al., 2005) was performed to investigate whether gene expression profiles of MCF10A cells with extra centrosomes (+DOX, 48 hrs) show bias towards specific signatures (http://software.broadinstitute.org/gsea/msigdb). In the GSEA, genes were ranked by Z score corresponding to false discovery rate (FDR) adjusted p values of the expression differences between normal cells and cells with extra centrosomes. 100,000 permutations were performed to assess the statistical significance of the enrichment score. The Gene Set for NRF2-regulated genes can be found here: http://software.broadinstitute.org/gsea/msigdb/cards/NFE2L2.V2.html.

CCLE Expression Analysis

Raw mRNA abundance values for cell lines were obtained from the Cancer Cell Line Encyclopedia (CCLE) (Barretina et al., 2012). Expression values for a subset of 14 cell lines were quantile normalized using Robust Multi-array Average (RMA). Each cell line was assigned to a comparative group based on its centrosome amplification; high (BT-549, CAL-120, HCC-1937, Hs578T, MDA-231), intermediate (HCC-1954, HCC-38, BT-474, HCC-1143, SK-BR-3, JIMT-1), and low (BT-20, MDA-468,

MCF-7) (Figure 7C). Boxplots of the mRNA abundance levels of cell lines appertaining to each group were generated for a group of pro-invasive factors.

Exosomes and Microvesicle Isolation

Isolation of microvesicles and exosomes was done by differential ultracentrifugation as described previously (Costa-Silva et al., 2015). Briefly, CM was collected as described above and cell debris was pelleted by centrifugation at 500*g* for 10 min. Microvesicles fraction was collected by centrifugation at 12,000*g* for 20 min. The supernatant was then centrifuge at 100,000*g* for 70 min in order to obtain the exosomes. The exosome pellet was washed in 20 ml of phosphate-buffered saline (PBS) and collected by ultracentrifugation at 100,000*g* for 70 min (Beckman Ti70). Both microvesicles and exosomes were resuspended in 3D media up to the initial volume in order to test their role in invasion.

DATA AND SOFTWARE AVAILABILITY

Microarray data of control MCF10A.PLK4 cells and MCF10A.PLK4 cells treated with DOX (48 hrs) to induce centrosome amplification is publicly available at ArrayExpress, accession number E-MTAB-6415.

QUANTIFICATION AND STATISTICAL ANALYSIS

Statistics

Appropriate statistical tests were applied as per described in each legend using GraphPad Prism 5.0. Briefly, student's *t*-tests were used for comparisons between two groups. One-way ANOVA with Tukey post hoc test were used for comparison of three or more groups with one independent variable. $*P < 0.05$, $**P < 0.01$, $***P < 0.001$, $****P < 0.0001$, ns not significant.

Developmental Cell, Volume *47*

Supplemental Information

Oxidative Stress in Cells with Extra Centrosomes

Drives Non-Cell-Autonomous Invasion

Teresa Arnandis, Pedro Monteiro, Sophie D. Adams, Victoria Louise Bridgeman, Vinothini Rajeeve, Emanuela Gadaleta, Jacek Marzec, Claude Chelala, Ilaria Malanchi, Pedro R. Cutillas, and Susana A. Godinho

Figure S1

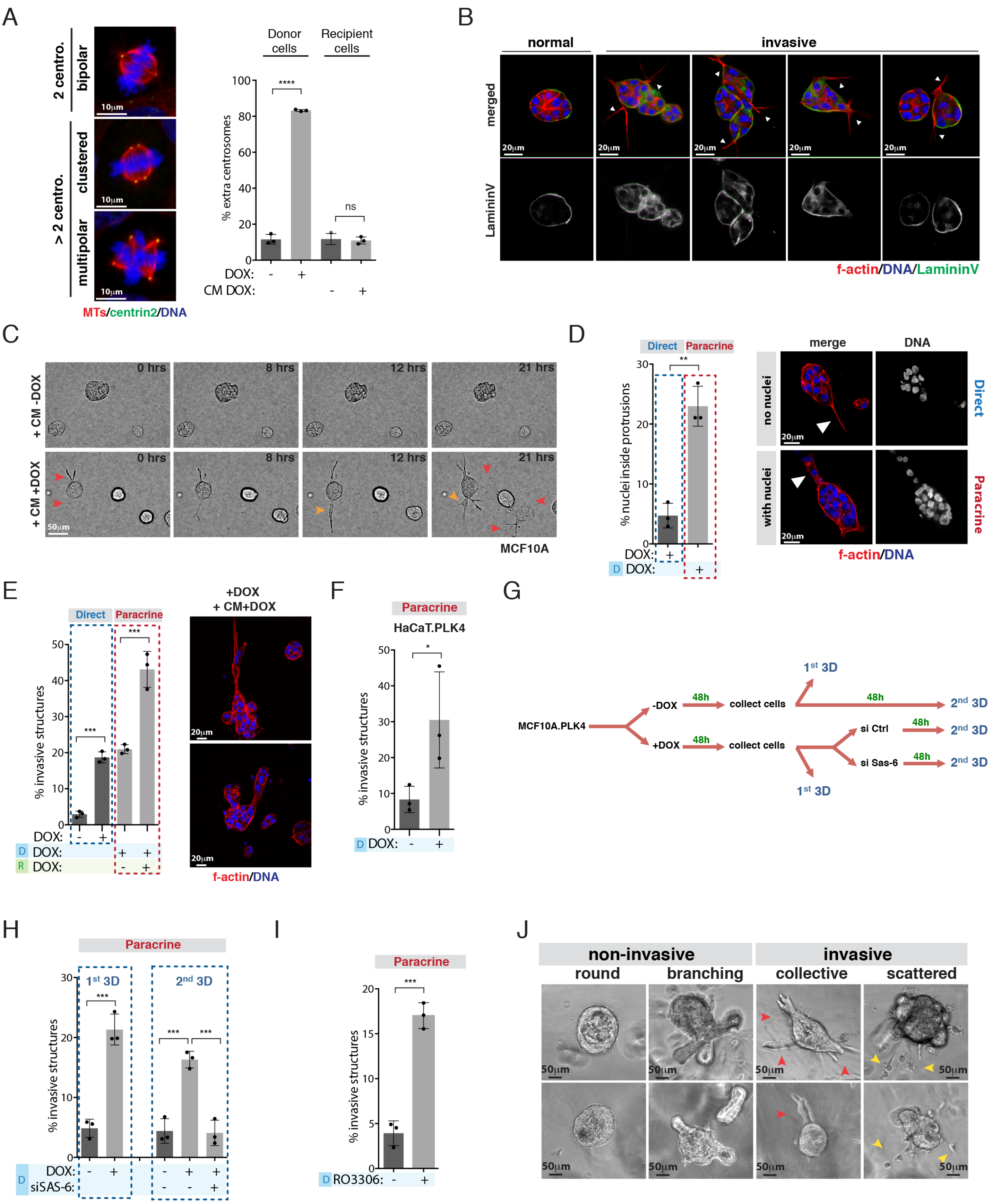
A
2 centro.
bipolar
> 2 centro.
clustered
multipolar
10μm
MTs/centrin2/DNA
Donor cells
Recipient cells
% extra centrosomes
****
ns
DOX: - +
CM DOX: - +
B
normal
invasive
merged
LamininV
20μm
f-actin/DNA/LamininV
C
+ CM -DOX
+ CM +DOX
0 hrs
8 hrs
12 hrs
21 hrs
50μm
MCF10A
D
Direct
Paracrine
% nuclei inside protrusions
**
DOX: +
D DOX: +
merge
DNA
no nuclei
with nuclei
f-actin/DNA
E
% invasive structures
***
DOX: - +
D DOX: + +
R DOX: - +
+DOX
+ CM+DOX
F
Paracrine
HaCaT.PLK4
*
D DOX: - +
G
MCF10A.PLK4
-DOX
+DOX
48h
collect cells
1st 3D
2nd 3D
si Ctrl
si Sas-6
H
1st 3D
2nd 3D
D DOX: - + - + +
siSAS-6: - - +
I
RO3306: - +
J
non-invasive
invasive
round
branching
collective
scattered
50μm

Figure S1. Centrosome amplification induces paracrine invasion. Related to Figure 1, Table S1, Videos S3 and S4. (A) Left, Cells in mitosis with normal and amplified centrosomes (>2 centro.; clustered + multipolar) were stained for microtubules (MTs) (α-Tubulin, red), centrioles (centrin2, green) and DNA (blue). Scale bar 10μm. Right, Centrosome amplification in cells with extra centrosomes (+DOX) or incubated with CM collected from cells with extra centrosomes (CM+DOX). (B) Normal and invasive 3D acini stained for f-actin (phalloidin, red), laminin V (green) and DNA (blue). White arrowheads indicate invasive protrusions. Scale bar 20μm. (C) Still images from live cell imaging of acini incubated with CM (CM-/+DOX). Red arrowheads indicate invasive acini and orange arrowhead indicates a cell/nuclei moving through the invasive protrusion. Scale bar 20μm (D) Left, Quantification of acini with invasive protrusions containing nuclei in cells with extra centrosomes (+DOX) or incubated with CM (CM-/+DOX). Right, Invasive acini stained for f-actin (phalloidin, red) and DNA (blue). Arrowheads indicate invasive protrusions. Scale bar 20μm. (E) Left, Quantification of invasive structures in acini with extra centrosomes (+DOX) and incubated with CM+DOX. Right, Highly invasive/disrupted acini observed in cells with extra centrosomes treated with CM+DOX. Cells were stained for f-actin (phalloidin, red) and DNA (blue). Scale bar 20μm. (F) Quantification of invasive acini incubated with CM from HaCaT.PLK4 cells. (G) Schematic representation of SAS-6 depletion to revert centrosome amplification. (H) Quantification of invasive acini incubated with CM from cells where centrosome amplification was lost (SAS-6 siRNA). (I) Invasive acini in cells incubated with CM collected from cells treated with the CDK1 inhibitor RO-3306 (5μM, 40 hrs). (J) Bright field images of non-invasive and invasive mouse mammary organoids. Scale bar 50μm. For all graphics error bars represent mean +/- SD from three independent experiments. *$P < 0.05$, **$P < 0.01$, ***$P < 0.001$, ****$P < 0.0001$, ns not significant.

Figure S2

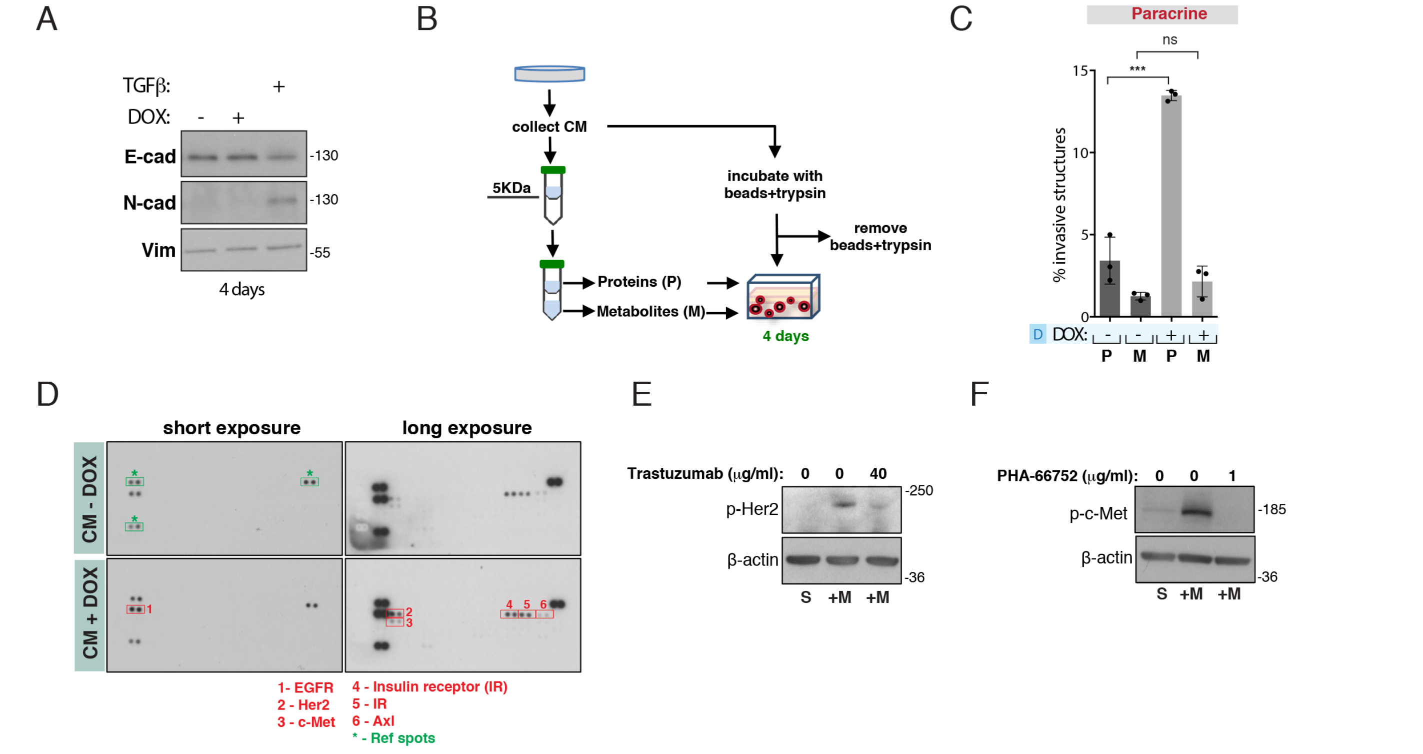

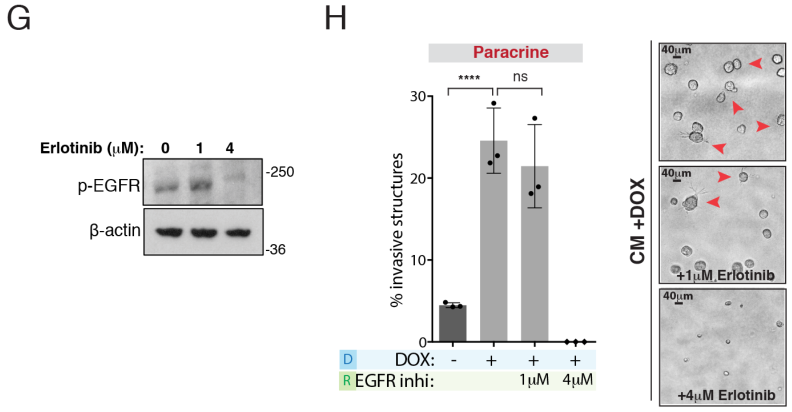

I

MCF10A.PLK4 - DOX					
sample	Initial cell #	Final cell #	Protein (mg/ml)	LDH (fold)	% invasion
1	$33.5e10^5$	$26e10^6$	16.58	1.0	3.36
2	$33.5e10^5$	$26e10^6$	16.01	1.0	3.24
3	$33.5e10^5$	$26e10^6$	17.86	0.9	4.79

MCF10A.PLK4 + DOX					
sample	Initial cell #	Final cell #	Protein (mg/ml)]	LDH (fold)	% invasion
1	$45.2e10^5$	$20e10^6$	19.11	1.1	15.91
2	$45.2e10^5$	$20e10^6$	19.24	1.2	17.45
3	$45.2e10^5$	$20e10^6$	20.63	1.3	17.67

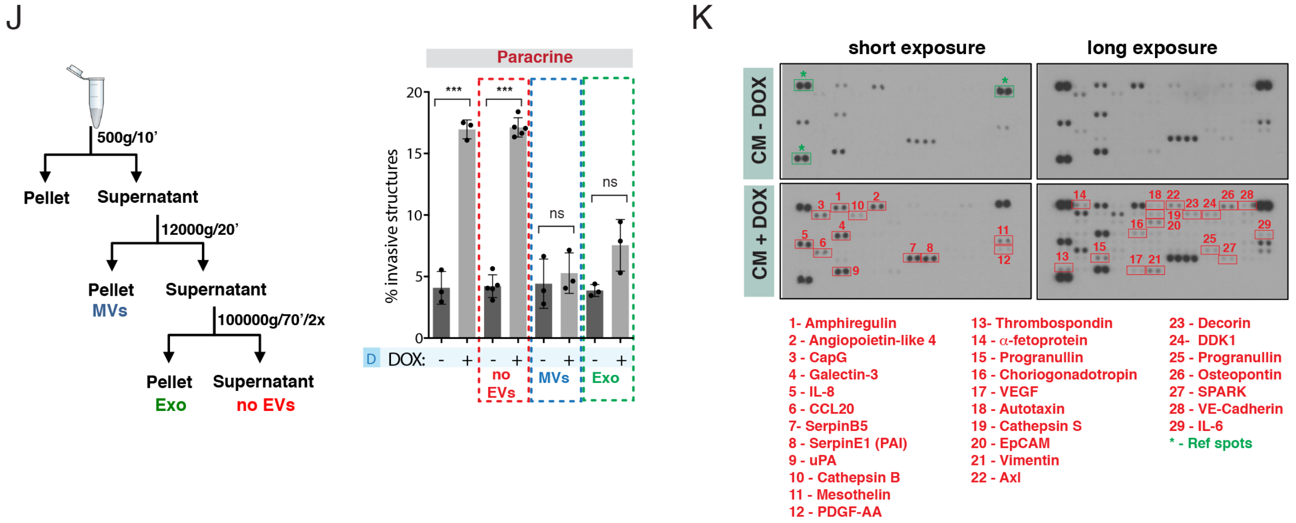

Figure S2. Proteins secreted by cells with extra centrosomes promote paracrine invasion via RTK activation. Related to Figure 2. (A) Levels of E-cadherin, N-cadherin and Vimentin in cells incubated with CM for 4 days. TGF-β treatment (5 ng/ml, 4 days) was used as positive control. (B) Schematic representation of Vivaspin filtration and tryspin incubation. (C) Quantification of invasive acini cells incubated with CM after Vivaspin filtration. P: proteins, M: metabolites. (D) phospho-RTK array obtained from cells incubated with CM. (E) phospho-Her2 levels in cells treated with 40 μg/ml of Trastuzumab (Her2 inhibitor) for 1 hr. S, serum starved; +M, plus fresh medium. (F) phospho-c-Met levels in cells treated with 1 μM PHA-66752 (c-Met inhibitor) for 1 hr. S, serum starved; +M, plus fresh medium. (G) phospho-EGFR levels in cells treated with different concentrations of Erlotinib (1 and 4 μM, 24 hrs). (H) Left, Quantification of invasive acini cells treated with EGFR inhibitor (Erlotinib, 1 and 4 μM) and incubated with CM. Right, Acinar structures. Red arrowheads indicate invasive acini. Scale bar 40μm. (I) Table summarising the conditions used to prepare CM for proteomic analysis. Cells with (+DOX) or without extra centrosomes (-DOX) were plated at different densities to ensure a similar cell number by the time of CM collection. Protein concentration and invasive capacity of the CM was assessed in all samples. (J) Left, Schematic representation of the ultracentrifugation method to isolate MVs and Exo. Right, Quantification of invasive acini in cells incubated with CM with and without MVs/Exo. (K) Human XL Oncology Array incubated with CM collected from cells with (CM+DOX) and without (CM-DOX) extra centrosomes. For all graphics error bars represent mean +/- SD from three independent experiments. ****P < 0.001, ****P < 0.0001,* ns not significant.

Figure S3

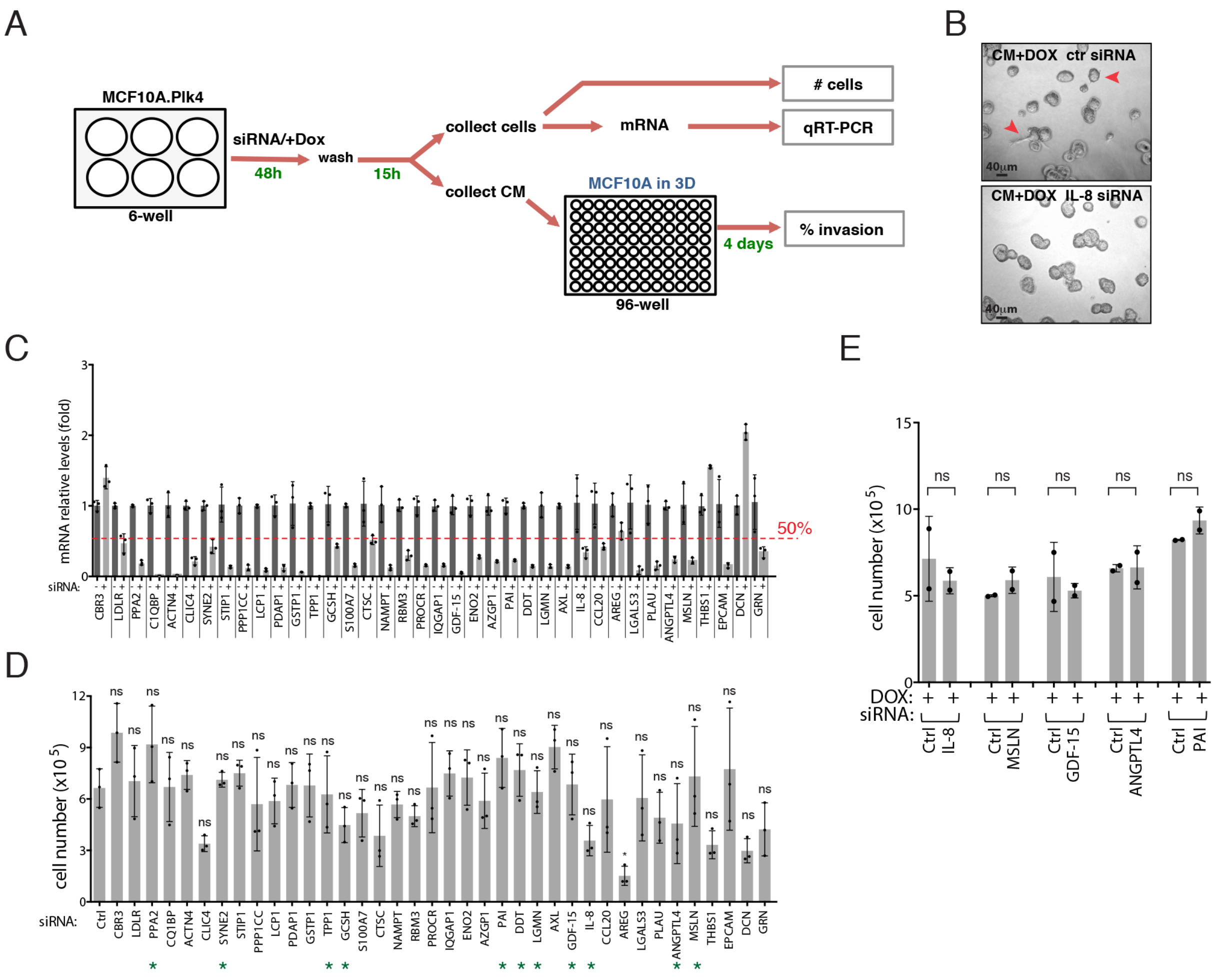
A
MCF10A.Plk4
6-well
siRNA/+Dox
48h
wash
15h
collect cells
mRNA
cells
qRT-PCR
collect CM
MCF10A in 3D
96-well
4 days
% invasion
B
CM+DOX ctr siRNA
CM+DOX IL-8 siRNA
40μm
C
mRNA relative levels (fold)
50%
siRNA:
CBR3 LDLR PPA2 C1QBP ACTN4 CLIC4 SYNE2 STIP1 PPP1CC LCP1 PDAP1 GSTP1 TPP1 GCSH S100A7 CTSC NAMPT RBM3 PROCR IQGAP1 GDF-15 ENO2 AZGP1 PAI DDT LGMN AXL IL-8 CCL20 AREG LGALS3 PLAU ANGPTL4 MSLN THBS1 EPCAM DCN GRN
D
cell number (x10 5)
siRNA:
Ctrl CBR3 LDLR PPA2 CQ1BP ACTN4 CLIC4 SYNE2 STIP1 PPP1CC LCP1 PDAP1 GSTP1 TPP1 GCSH S100A7 CTSC NAMPT RBM3 PROCR IQGAP1 ENO2 AZGP1 PAI DDT LGMN AXL GDF-15 IL-8 CCL20 AREG LGALS3 PLAU ANGPTL4 MSLN THBS1 EPCAM DCN GRN
ns
E
cell number (x10 5)
DOX:
siRNA:
Ctrl IL-8
Ctrl MSLN
Ctrl GDF-15
Ctrl ANGPTL4
Ctrl PAI
ns

Figure S3. siRNA screen identified secreted pro-invasive factors in MCF10A cells with extra centrosomes. Related to Figure 3 and Table S1. (A) Schematic representation of the siRNA screen set-up. (B) Acini images from 96-well plate siRNA screen. Scale bar 40μm. (C) mRNA levels for all the siRNA conditions to assess depletion efficiency. Red dashed line represents 50% reduction of mRNA levels. (D) Cell number upon siRNA treatment to assess the impact of the different conditions on cell proliferation/viability. Green asterisks depict the positive screen hits shown in Figure 3F. (E) Cell number in cells with extra centrosomes (+DOX) upon siRNA depletion of some of our positive hits. For all graphics error bars represent mean +/- SD from three independent experiments. *$P < 0.05$, ns not significant.

Figure S4

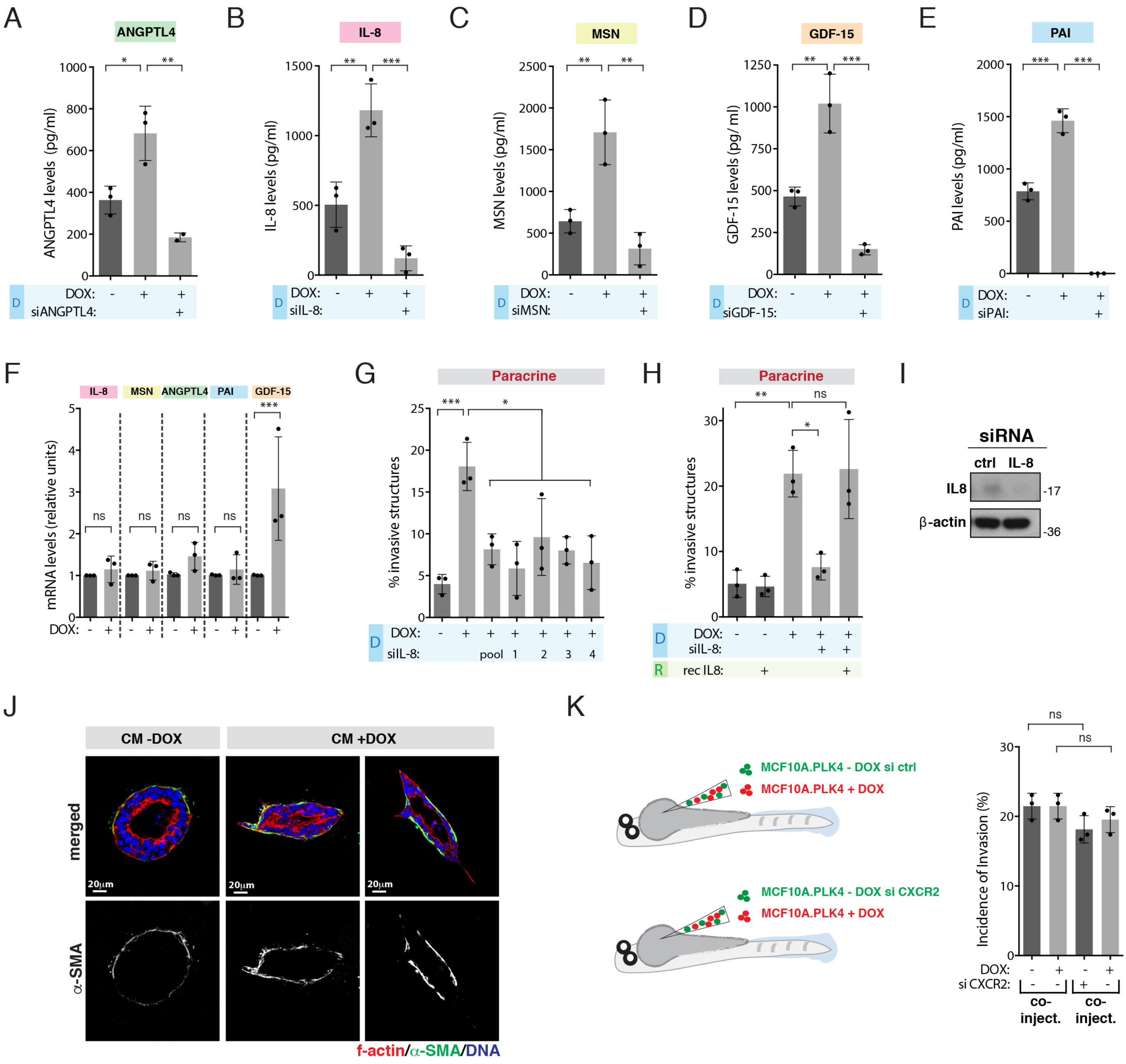

A
ANGPTL4
ANGPTL4 levels (pg/ml)
DOX: - + +
siANGPTL4: +
B
IL-8
IL-8 levels (pg/ml)
DOX: - + +
siIL-8: +
C
MSN
MSN levels (pg/ml)
DOX: - + +
siMSN: +
D
GDF-15
GDF-15 levels (pg/ ml)
DOX: - + +
siGDF-15: +
E
PAI
PAI levels (pg/ml)
DOX: - + +
siPAI: +
F
IL-8 MSN ANGPTL4 PAI GDF-15
mRNA levels (relative units)
DOX:
G
Paracrine
% invasive structures
DOX: - + + + + + +
siIL-8: pool 1 2 3 4
H
Paracrine
% invasive structures
DOX: - - + + +
siIL-8: + +
rec IL8: + +
I
siRNA
ctrl IL-8
IL8 -17
β-actin -36
J
CM -DOX
CM +DOX
merged
α-SMA
20µm
f-actin/α-SMA/DNA
K
MCF10A.PLK4 - DOX si ctrl
MCF10A.PLK4 + DOX
MCF10A.PLK4 - DOX si CXCR2
MCF10A.PLK4 + DOX
Incidence of Invasion (%)
DOX: - + - +
si CXCR2: - - + -
co-inject.
co-inject.

Figure S4. Validation of the positive hits identified in the siRNA screen. Related to Figure 4. (A) Levels of secreted ANGPTL4. (B) Levels of secreted IL-8. (C) Levels of secreted MSN. (D) Levels of secreted GDF-15. (E) Levels of secreted PAI. (F) mRNA levels of the different pro-invasive factors after induction of centrosome amplification (48 hrs). (G) Quantification of invasive structures in acini incubated with CM collected from cells depleted of IL-8 (siRNA pool and individual sequences). (H) Quantification of invasive structures in acini incubated with CM collected from cells depleted of IL-8 and supplemented with recombinant IL-8 (0.5 μg/ml). (I) IL-8 levels after siRNA depletion. (J) Non-invasive and invasive mammary organoids from WT mice stained for f-actin (phalloidin, red), α-SMA (green) and DNA (blue). Scale bar 20μm. (K) Left, Schematic representation of the zebrafish co-injections. Right, Quantification of the incidence of invasion in the co-injection experiments (-DOX/+DOX) when CXCR2 is depleted in cells with normal centrosomes (-DOX). Number of injected fish co-injection control siRNA=71; co-injection CXCR2 siRNA=121. For all graphics error bars represent mean +/- SD from three independent experiments. *$P < 0.05$, **$P < 0.01$, ***$P < 0.001$, ns not significant.

Figure S5

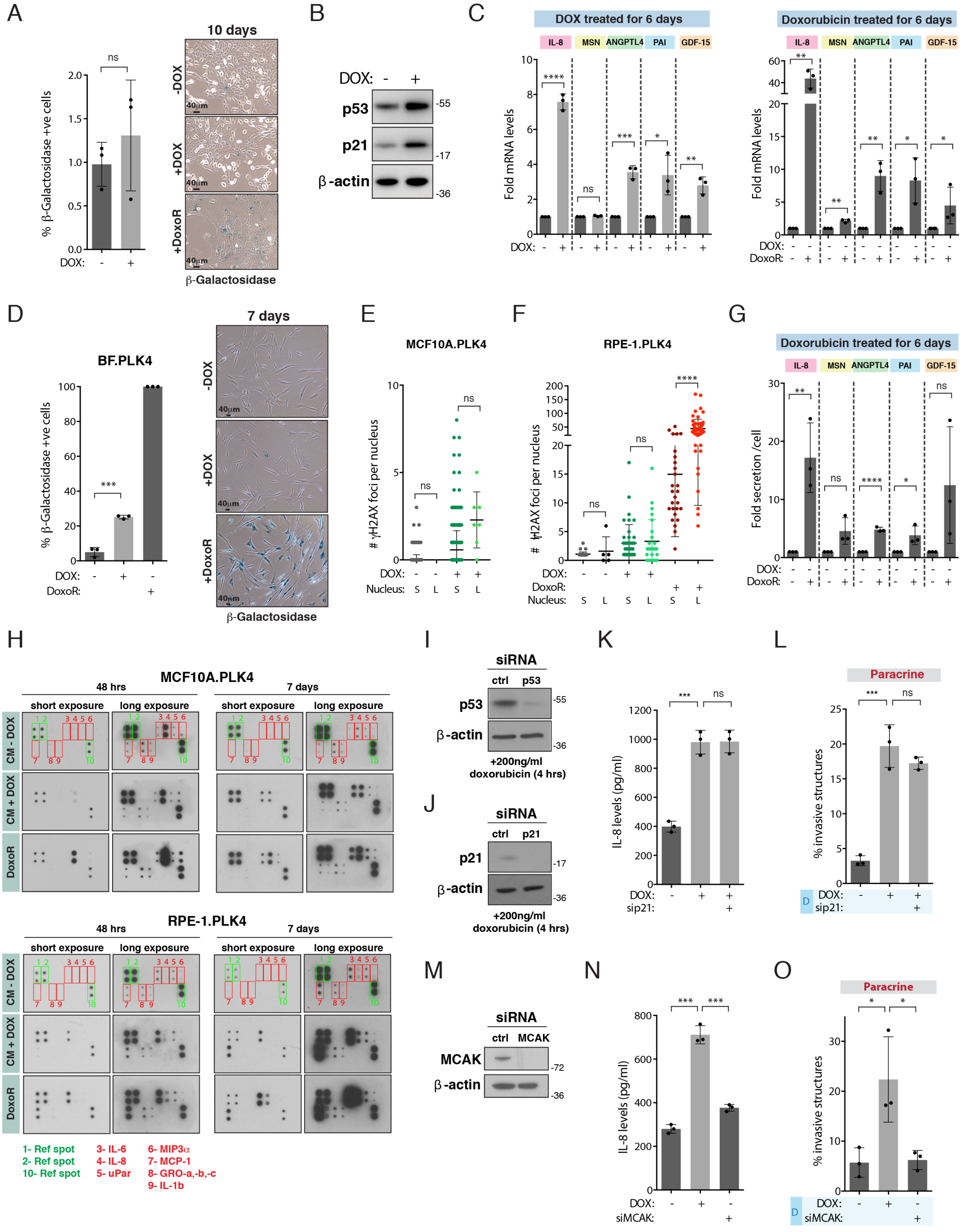

A
10 days
ns
% β-Galactosidase +ve cells
DOX: - +
-DOX
+DOX
+DoxoR
40µm
β-Galactosidase
B
DOX: - +
p53
p21
β-actin
-55
-17
-36
C
DOX treated for 6 days
Doxorubicin treated for 6 days
IL-8
MSN
ANGPTL4
PAI
GDF-15
Fold mRNA levels
DOX:
DoxoR:
D
BF.PLK4
7 days
% β-Galactosidase +ve cells
DOX: - + -
DoxoR: +
β-Galactosidase
E
MCF10A.PLK4
γH2AX foci per nucleus
Nucleus: S L S L
F
RPE-1.PLK4
G
Doxorubicin treated for 6 days
Fold secretion /cell
H
MCF10A.PLK4
48 hrs
7 days
short exposure
long exposure
CM - DOX
CM + DOX
DoxoR
RPE-1.PLK4
1- Ref spot
2- Ref spot
10- Ref spot
3- IL-6
4- IL-8
5- uPar
6- MIP3α
7- MCP-1
8- GRO-a,-b,-c
9- IL-1b
I
siRNA
ctrl p53
p53
β-actin
+200ng/ml doxorubicin (4 hrs)
J
siRNA
ctrl p21
p21
β-actin
+200ng/ml doxorubicin (4 hrs)
K
IL-8 levels (pg/ml)
DOX: - + +
sip21: +
L
Paracrine
% invasive structures
DOX: - + +
sip21: +
M
siRNA
ctrl MCAK
MCAK
β-actin
-72
-36
N
IL-8 levels (pg/ml)
DOX: - + -
siMCAK: +
O
Paracrine
% invasive structures
DOX: - + -
siMCAK: +

Figure S5. Secretion of pro-invasive factors in cells with extra centrosomes does not require a full senescence response and is not a consequence of aneuploidy. Related to Figure 5 and Table S1. (A) Left, Quantification of β-galactosidase positive MCF10A.PLK4 cells after induction of centrosome amplification for 10 days. Right, Images representing cells stained for β-galactosidase (blue). Scale bar 40μm. (B) Levels of p53 and p21 after induction of extra centrosomes (48 hrs). (C) mRNA levels of the different pro-invasive factors 6 days after induction of centrosome amplification (Left) or Doxorubicin treatment (Right). (D) Left, Quantification of β-galactosidase positive BF.PLK4 cells 7 days after induction of centrosome amplification or doxorubicin treatment (100 ng/ml). Right, Cells stained for β-galactosidase (blue). Scale bar 40μm. (E) Quantification of γH2AX foci in MCF10A cells with extra centrosomes after 7days. (F) Quantification of γH2AX foci in RPE-1 cells with extra centrosomes after 7days. DoxoR-induced senescent cells were used as positive control. L, large nuclei; S, small nuclei. Data as seen in Fig. 5f. (G) Quantification of secreted pro-invasive factors in senescent cells treated with DoxoR after 6 days. (H) SASP array incubated with CM collected from cells with (CM+DOX) and without (CM-DOX) extra centrosomes. DoxoR-induced senescent cells were used as positive control. (I) Levels of p53 after p53 siRNA. (J) Levels of p21 after p21 siRNA. (K) Levels of secreted IL-8 in cells depleted of p21. (L) Quantification of invasive structures in acini incubated with CM collected from cells depleted of p21. (M) Levels of MCAK after MCAK siRNA depletion. (N) Levels of secreted IL-8 in cells depleted of MCAK. (O) Quantification of invasive structures in acini incubated with CM collected from cells depleted of MCAK. For all graphics error bars represent mean +/- SD from three independent experiments. *$P < 0.05$, **$P < 0.01$, ***$P < 0.001$, ****$P < 0.0001$, ns not significant.

Figure S6

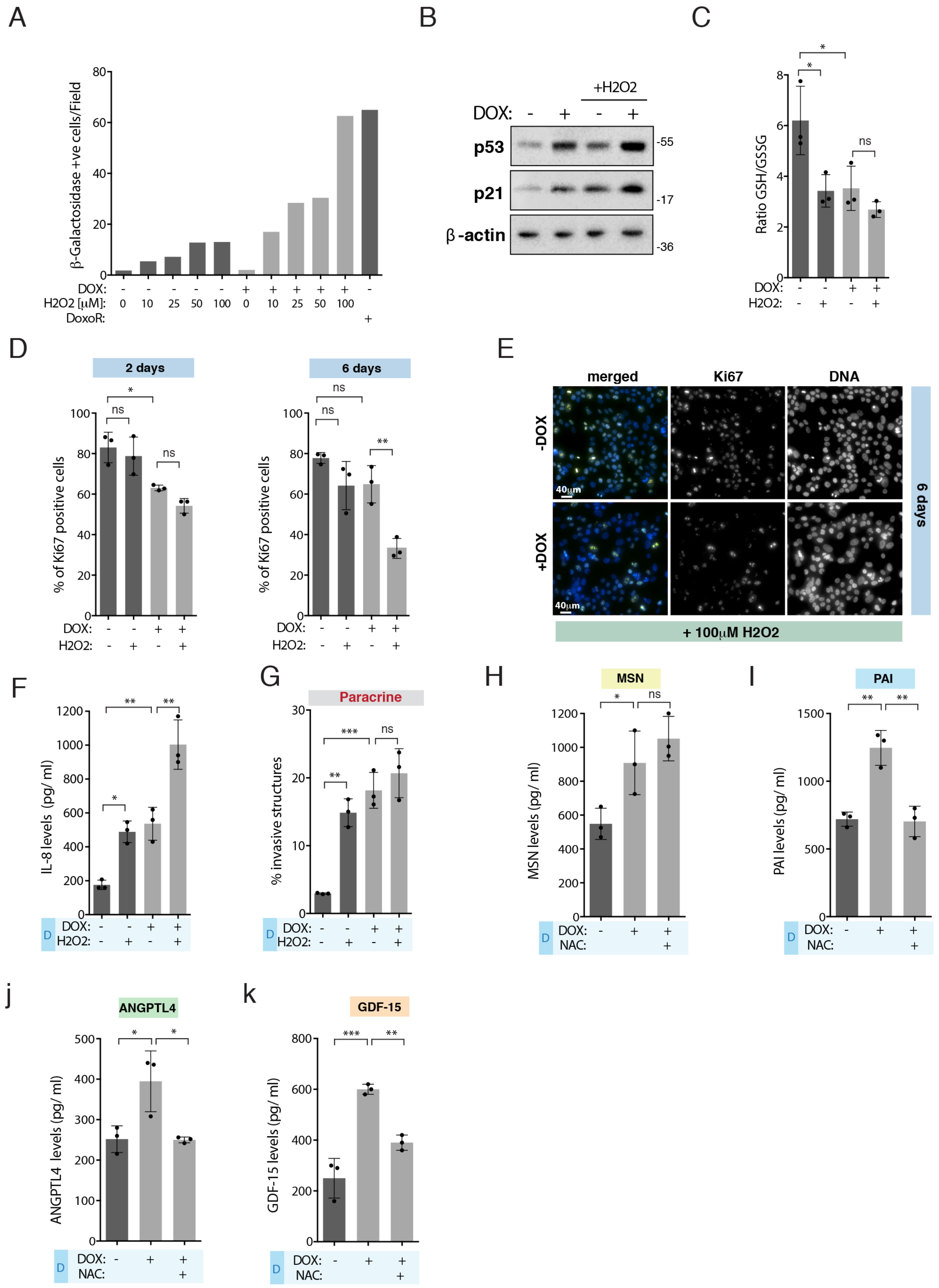

A
B
C
D
E
F
G
H
I
j
k
β-Galactosidase +ve cells/Field
DOX:
H2O2 [μM]:
DoxoR:
+H2O2
p53
p21
β-actin
-55
-17
-36
Ratio GSH/GSSG
2 days
6 days
% of Ki67 positive cells
merged
Ki67
DNA
-DOX
+DOX
6 days
+ 100μM H2O2
40μm
IL-8 levels (pg/ ml)
Paracrine
% invasive structures
MSN
MSN levels (pg/ ml)
PAI
PAI levels (pg/ ml)
NAC:
ANGPTL4
ANGPTL4 levels (pg/ ml)
GDF-15
GDF-15 levels (pg/ ml)

Figure S6. Increased ROS levels in cells with extra centrosomes promote secretion and paracrine invasion. Related to Figure 6 and Table S1. (A) Quantification of β-galactosidase positive MCF10A.PLK4 cells after 6 days, before and after 48 hrs treatment with DOX and treated with different doses of H2O2 for 48 hrs. (B) Levels of p53 and p21 in cells with (+DOX) and without (-DOX) extra centrosomes treated with H2O2 (100 μM). (C) Ratio of GSH/GSSG in cells after induction of extra centrosomes (48 hrs) and H2O2 (100 μM) treatment. (D) Quantification of Ki67 positive cells 2 and 6 days after induction of centrosome amplification and H2O2 (100 μM) treatment. (E) Cells stained for Ki67 (green) and DNA (blue). Scale bar 40μm. (F) Levels of secreted IL-8 in cells with (+DOX) and without (-DOX) extra centrosomes treated with H2O2 (100 μM, 48 hrs). (G) Quantification of invasive structures in acini incubated with CM collected from cells with (+DOX) and without (-DOX) extra centrosomes treated with H2O2 (100 μM). (H) Levels of secreted MSN after NAC treatment (5 mM, 48 hrs). (I) Levels of secreted PAI after NAC treatment (5 mM, 48 hrs). (J) Levels of secreted ANGPTL4 after NAC treatment (5 mM, 48 hrs). (K) Levels of secreted GDF-15 after NAC treatment (5 mM, 48 hrs). For all graphics error bars represent mean +/- SD from three independent experiments. *$P < 0.05$, **$P < 0.01$, ***$P < 0.001$, ns not significant.

Figure S7

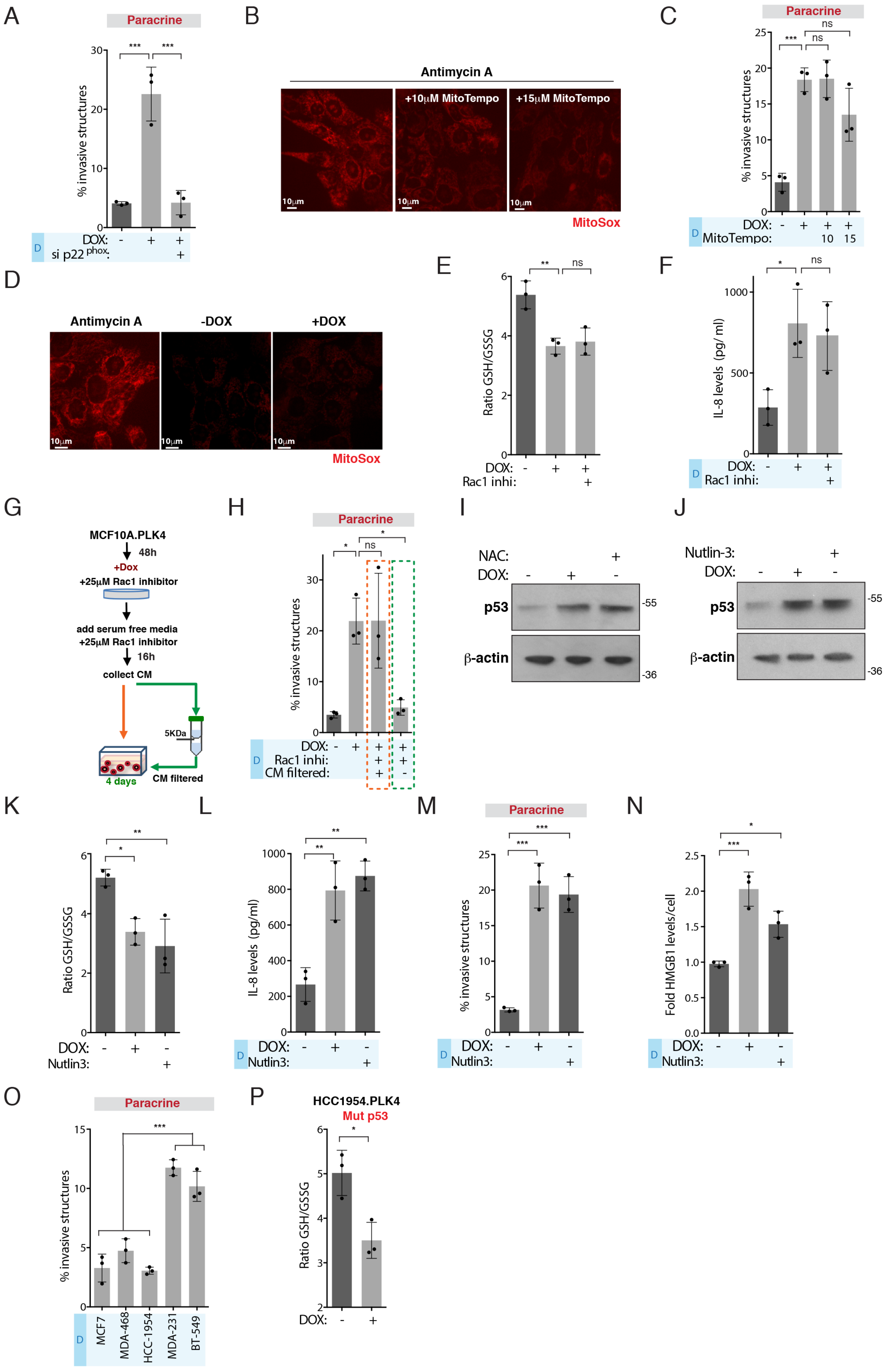

A
Paracrine
% invasive structures
DOX: - + +
si p22 phox: +
B
Antimycin A
+10μM MitoTempo
+15μM MitoTempo
MitoSox
C
Paracrine
% invasive structures
DOX: - + + +
MitoTempo: 10 15
D
Antimycin A
-DOX
+DOX
MitoSox
E
Ratio GSH/GSSG
DOX: - + +
Rac1 inhi: +
F
IL-8 levels (pg/ ml)
DOX: - + +
Rac1 inhi: +
G
MCF10A.PLK4
48h
+Dox
+25μM Rac1 inhibitor
add serum free media
+25μM Rac1 inhibitor
16h
collect CM
5KDa
4 days
CM filtered
H
Paracrine
% invasive structures
DOX: - + + +
Rac1 inhi: + +
CM filtered: + -
I
NAC: +
DOX: - + -
p53
β-actin
J
Nutlin-3: +
DOX: - + -
p53
β-actin
K
Ratio GSH/GSSG
DOX: - + -
Nutlin3: +
L
IL-8 levels (pg/ml)
DOX: - + -
Nutlin3: +
M
Paracrine
% invasive structures
DOX: - + -
Nutlin3: +
N
Fold HMGB1 levels/cell
DOX: - + -
Nutlin3: +
O
Paracrine
% invasive structures
MCF7
MDA-468
HCC-1954
MDA-231
BT-549
P
HCC1954.PLK4
Mut p53
Ratio GSH/GSSG
DOX: - +

Figure S7. p53-mediated ROS production promotes secretion and paracrine invasion in cells with extra centrosomes. Related to Figure 7 and Table S1. (A) Quantification of invasive structures in acini incubated with CM collected from cells depleted of p22^{phox}. (B) Cells treated with Antimycin A (35 μM) to increase mitochondrial ROS and stained with fluorogenic dye Mitosox (red). MitoTempo was used to inhibit mitochondrial ROS (10 and 15 μM, 48 hrs). Scale bar 10μm. (C) Quantification of invasive structures in acini incubated with CM collected from cells with extra centrosomes treated with MitoTempo (10 and 15 μM, 48 hrs). (D) Cells were stained with MitoSox (red). Scale bar 10μm. (E) Ratio of GSH/GSSG in cells after induction of extra centrosomes (48 hrs) and treated with RAC1 inhibitor (NSC23766, 25 μM, 48 hrs). (F) Levels of secreted IL-8 in cells with (+DOX) and without (-DOX) extra centrosomes treated with RAC1 inhibitor (25 μM, 48 hrs). (G) Schematic representation of the experimental setting to remove RAC1 inhibitor from the CM before adding on 3D cultures. (H) Quantification of invasive structures in acini incubated with CM collected from cells treated with RAC1 inhibitor (25 μM) before and after removal of the drug (CM filtered). (I) p53 levels in cells with extra centrosomes (+DOX) and treated with NAC (5 mM, 48 hrs). (J) Western blot analysis of p53 levels in cells treated with Nutlin-3 (5 mM, 48 hrs). (K) Ratio of GSH/GSSG in cells treated with Nutlin-3 (5 mM, 48 hrs). (L) Levels of secreted IL-8 in cells treated with Nutlin-3 (5 mM, 48 hrs). (M) Quantification of invasive structures in acini incubated with CM collected from cells treated Nutlin-3 (5 mM, 48 hrs). (N) Levels of secreted HMGB1 in cells treated with Nutlin-3 (5 mM, 48 hrs). (O) Quantification of invasive structures in acini incubated with CM collected from breast cancer cell lines with low and high centrosome amplification. (P) Ratio of GSH/GSSG in HCC1954.PLK4 cells after induction of extra centrosomes (+DOX) for 48 hrs. For all graphics error bars represent mean +/- SD from three independent experiments. *$P < 0.05$, **$P < 0.01$, ***$P < 0.001$, ns not significant.

Table S1: Quantification of the percentage of extra centrosomes in the different conditions and cell lines. Related to Figures 1, 3, 5, 6, 7, S1, S3, S5, S6 and S7.

Cell line	Treatment(s)	% Centrosome amplification
**MCF10A.PLK4**	n/a*	12
**MCF10A.PLK4**	DOX	87
**MCF10A.PLK4-608**	DOX	11
**MCF10A.PLK4**	after DOX + siRNA ctr	42
**MCF10A.PLK4**	after DOX + siRNA SAS-6	18
**HaCat.PLK4**	n/a*	21
**HaCaT.PLK4**	DOX	73
**MCF10A**	RO-3306	58
**MCF10A.PLK4**	DOX + siRNA IL-8	66
**MCF10A.PLK4**	DOX + siRNA ANGPTL4	78
**MCF10A.PLK4**	DOX + siRNA GDF15	65
**MCF10A.PLK4**	DOX + siRNA PAI	71
**MCF10A.PLK4**	DOX + siRNA MSN	61
**MCF10A.PLK4**	DOX + siRNA p53	64
**MCF10A.PLK4**	DOX + siRNA p21	65
**MCF10A.PLK4**	DOX 6 days	39
**MCF10A.PLK4**	DOX + NAC	87
**MCF10A.PLK4**	DOX + Apocynin	93
**MCF10A**	Nutlin-3	8
**MCF10A.PLK4**	DOX + Rac inhibitor	82
**MCF10A.PLK4**	H2O2	8
**MCF10A.PLK4**	DOX + H2O2	46
**MCF7. PLk4**	n/a*	18
**MCF7. PLk4**	DOX	57
**HCC1954.PLK4**	n/a*	16
**HCC1954. PLK4**	DOX	82
**BF.PLK4**	n/a*	1
**BF.PLK4**	DOX	44
**RPE.PLK4**	n/a*	15
**RPE.PLK4**	DOX	80

* n/a: not applicable, no treatment performed

Supplementary Table 6: References for all siRNAs used. Related to Figures 3 and S3.

Gene symbol	Reference	Company
**CXCL8**	M-004756-00	Dharmacon
**CCL20**	M-007832-01	Dharmacon
**AREG**	M-017435-00	Dharmacon
**LGALS3**	M-010606-02	Dharmacon
**PLAU**	M-006000-02	Dharmacon
**ANGPTL4**	M-007807-02	Dharmacon
**MSLN**	M-006346-02	Dharmacon
**THBS1**	M-019743-01	Dharmacon
**EPCAM**	M-004568-03	Dharmacon
**DCN**	M-021491-00	Dharmacon
**GRN**	M-009285-02	Dharmacon
**SYNE2**	D-019259-01	Dharmacon
**CBR3**	L-008597-00	Dharmacon
**LDLR**	L-011073-00	Dharmacon
**PPA2**	L-012348-00	Dharmacon
**C1QBP**	L-011225-01	Dharmacon
**ACTN4**	L-011988-00	Dharmacon
**CLIC4**	L-013553-00	Dharmacon
**STIP1**	L-019802-00	Dharmacon
**PPP1CC**	L-006827-00	Dharmacon
**LCP1**	L-011716-00	Dharmacon
**PDAP1**	L-017675-00	Dharmacon
**GSTP1**	L-011179-00	Dharmacon
**TPP1**	L-005810-00	Dharmacon
**GCSH**	L-017907-00	Dharmacon
**S100A7**	L-011769-02	Dharmacon
**CTSC**	L-005835-00	Dharmacon
**NAMPT**	L-004581-00	Dharmacon
**RBM3**	L-018969-01	Dharmacon
**PROCR**	L-017326-00	Dharmacon
**IQGAP1**	L-004694-00	Dharmacon
**GDF15**	L-019875-00	Dharmacon
**ENO2**	L-009777-00	Dharmacon
**AZGP1**	L-012567-01	Dharmacon
**SERPINE1**	L-019376-01	Dharmacon
**DDT**	L-012201-01	Dharmacon
**LGMN**	L-005924-00	Dharmacon
**AXL**	L-003104-00	Dharmacon
**CEP192**	L-032250-01	Dharmacon
**P21**	L-003471-00	Dharmacon
**SAS6**	L-019156-01	Dharmacon
**P53**	L-003329-00	Dharmacon
**CXCR2**	L-005647-00	Dharmacon
**CYBA**	L-011020-02	Dharmacon
**CONTROL**	1027310	Qiagen

Supplementary Table 7: Primer sequences used in the RT-qPCR. Related to Figures S3, S4 and S5.

Gene symbol	Primer Forward 5'-3'	Primer Reverse 5'-3'
**GAPDH**	TTAAAAGCAGCCCTGGTGAC	CTCTGCTCCTCCTGTTCGAC
**CXCL8**	GTTTTTGAAGAGGGCTGAG	TTTGCTTGAAGTTTCACTGG
**CCL20**	TATATTGTGCGTCTCCTCAG	GCTATGTCCAATTCCATTCC
**AREG**	AAAGAAAGAAAAAGGGAGGC	CATTTGCATGTTACTGCTTC
**LGALS3**	AGATTTCCAAAGAGGGAATG	AAGTGCAAACAATGACTCTC
**PLAU**	GAAAACCTCATCCTACACAAG	ATTCTCTTTTCCAAAGCCAG
**ANGPTL4**	AGGCAGAGTGGACTATTTG	CCTCCATCTGAGGTCATC
**MSLN**	GACGTCCTAAAGCATAAACTG	TCATCTTGAGGAAGAGGTAG
**THBS1**	GTGACTGAAGAGAACAAAGAG	CAGCTATCAACAGTCCATTC
**EPCAM**	GTCTGAGAAGGCTGAGATAAAG	CTTCAAAGATGTCTTCGTCC
**DCN**	TTCACGCATTGATTCTTGTC	GCTGATTCTTGGACAGATAAAG
**GRN**	GACCTGATCCAGAGTAAGTG	CATGTCACATTTCACATCCC
**SYNE2**	GAGAAGATAGAAGAAGCACTC	TCTTATAGGTTTTCTGCTGC
**CBR3**	AGTGAGGTGCTAGTTTCCAAGG	CTGCACATTCTTGTGGACCC
**LDLR**	GAGGACAAAGTATTTTGGACAG	GTAGGTTTTCAGCCAACAAG
**PPA2**	CTGGAAGCTACTCTTAATTGG	GCCTTGTTTTTGAATTCTCC
**C1QBP**	AACATTAACAACAGCATCCC	TCATCCTCTGGATAATGACAG
**ACTN4**	AGTATGACAAGCTGAGGAAG	CTGAAAAGGCATGGTAGAAG
**CLIC4**	CCCAGAATCAAATCATGCTG	TCAATTTCATCAGGGAGAGG
**STIP1**	AACGAGTGTTTTCAGAAAGG	TATAACCCTTGATGAAGGTCG
**PPP1CC**	GAGGTTTATCACCAGATCTTC	CAGCCTAAGACATGTTTATCG
**LCP1**	AAGCTCTGATTGCTCTTTTG	GAAGTTGCCAATTTTGTTGC
**PDAP1**	AAGATGACTACCAGCAAAAG	CTGCTTCTCAATCTCTTCTC
**GSTP1**	GCAAATACATCTCCCTCATC	GTCTCAAAAGGCTTCAGTTG
**TPP1**	AATAACCTGACCCTAGAGAATG	CCACATAGTGATGAAACTCAG
**GCSH**	TTGTTATGAAGATGGTTGGC	TCTAAGTCTTCTATCCACCAC
**S100A7**	TTAGTGCCTGTGACAAAAAG	GTAGTCTGTGGCTATGTCTC
**CTSC**	AACAAACTGGCCATGAAC	ATAAAGACTCCAGAAGGGAC
**NAMPT**	CTAATGGCCTTGGGATTAAC	TCCAGTGTAACAAAATTCCC
**RBM3**	AGTGGCAGGTATTATGACAG	TCTGCCATTATAGTCTCTGG
**PROCR**	CATATGAAGTCTTTGGAGGC	CATATGGAAGTCTTTGGAGGC
**IQGAP1**	ATGCCTTTGACATCATTGAC	TCTAGGTTTCTGGTAGGACTG
**GDF15**	CGAAGACTCCAGATTCCG	ACTTCTGGCGTGAGTATC
**ENO2**	ATGTCCATAGAGAAGATCTGG	GACACCTTTGCCTAAGTAAC
**AZGP1**	ACAGAAATCACAGTCAATGG	TCCAAGTCTACTCAAGACAG
**SERPINE1**	ATCCACAGCTGTCATAGTC	CACTTGGCCCATGAAAAG
**DDT**	CCCACTTCTTTGAGTTTCTC	ATCTCTCTGGAAGAAGCAG
**LGMN**	ACTATGATGAGAAGAGGTCC	GGTGGAGATTGTTTTGTTTC
**AXL**	CATGAAACATGGAGACCTAC	ATCTCTTGGTACTCAGATACTC
